# CF_3_SO_2_X (X = Na, Cl) as reagents for trifluoromethylation, trifluoromethylsulfenyl-, -sulfinyl- and -sulfonylation. Part 1: Use of CF_3_SO_2_Na

**DOI:** 10.3762/bjoc.13.272

**Published:** 2017-12-19

**Authors:** Hélène Guyon, Hélène Chachignon, Dominique Cahard

**Affiliations:** 1 UMR 6014 CNRS COBRA, Normandie Université, 1 rue Tesnière, 76821 Mont Saint Aignan, France

**Keywords:** fluorine, sulfur, trifluoromethylation, trifluoromethylsulfenylation, trifluoromethylsulfonylation

## Abstract

Sodium trifluoromethanesulfinate, CF_3_SO_2_Na, and trifluoromethanesulfonyl chloride, CF_3_SO_2_Cl, are two popular reagents that are widely used for the direct trifluoromethylation of a large range of substrates. Further, these two reagents are employed for the direct trifluoromethylsulfenylation and trifluoromethylsulfinylation, the introduction of the SCF_3_ and the S(O)CF_3_ group, respectively. In addition to the aforementioned reactions, the versatility of these two reagents is presented in other reactions such as sulfonylation and chlorination. This first part is dedicated to sodium trifluoromethanesulfinate.

## Introduction

In organofluorine chemistry, the CF_3_ group occupies a place of choice as privileged structural motif in the development of multifaceted catalysts and ligands for organic synthesis as well as in the design of pharmaceuticals, agrochemicals and specialty materials [1–4]. The trifluoromethyl group is most of the time linked to a carbon atom but can also be encountered with chalcogens (S, Se, O) and nitrogen. The CF_3_S motif, which was till recently considered as an emerging substituent, is henceforth a tamed substituent as a result of abundant literature describing methods to prepare CF_3_S-featuring molecules [5]. Its oxidised congeners CF_3_S(O) and CF_3_SO_2_ are also well-developed as structural units in biologically active compounds, catalysts for synthesis, and functional materials. The chemistry of CF_3_Se derivatives is less developed but basically shares the same synthetic approaches with CF_3_S analogues [6–7]. The CF_3_O group is also of high interest but difficulties still exist to easily introduce this motif directly onto organic molecules [8]. The reasons behind such massive interest for the CF_3_ group are due to the specific physical and chemical properties of the compounds that contain it. The CF_3_ group has a large van der Waals volume somewhere between those of the iPr and the *t*-Bu groups [9]. Its electronegativity is comparable to that of oxygen (4.0 versus 3.5 on the Pauling scale) and its hydrophobicity is large (0 for H, 0.51 for Me, and 1.07 for CF_3_) [10]. In this respect, judicious installation of CF_3_ group(s) in catalysts or ligands is an effective tool to tune their reactivity and selectivity in synthesis. As a pharmacophore, CF_3_ substantially improves the catabolic stability, lipophilicity, and transport rate. In association with chalcogens (OCF_3_, SCF_3_, SeCF_3_), the CF_3_ group imparts enhanced lipophilicity of aromatic compounds in comparison with aryl–CF_3_ analogues (Hansch’s hydrophobic parameter π(SCF_3_) = 1.44; π(OCF_3_) = 1.04 versus π(CF_3_) = 0.88). Trifluoromethyl sources are manifold displaying nucleophilic, electrophilic or radical reactivities [11]. The most popular nucleophilic trifluoromethylating reagent is certainly the trifluoromethyltrimethylsilane, CF_3_SiMe_3_, known as the Ruppert–Prakash reagent discovered in 1984 by Ruppert and applied for trifluoromethylation in 1989 by Prakash and Olah. More recently, renewed investigation on the use of fluoroform, CF_3_H, as an ideal source of trifluoromethide offered new horizons for atom-economical, low-cost trifluoromethylation reactions. With regard to electrophilic CF_3_ donors, *S*-(trifluoromethyl)sulfonium salts developed by Yagupolskii and Umemoto and hypervalent iodine(III)-CF_3_ reagents developed by Togni are widely employed in a variety of trifluoromethylation reactions [12]. Traditional radical CF_3_ sources include the gaseous trifluoroiodomethane CF_3_I and trifluorobromomethane CF_3_Br. More conveniently, the liquid trifluoromethanesulfonyl chloride CF_3_SO_2_Cl and the solid sodium trifluoromethanesulfinate CF_3_SO_2_Na that are commercially available at reasonable prices, are easy-to-handle sources of trifluoromethyl radicals. Remarkably, CF_3_SO_2_Na and CF_3_SO_2_Cl are multi-purpose reagents since they not only act as CF_3_ donors by extrusion of SO_2_, but also, under certain reaction conditions, the sulfur atom is retained for trifluoromethylsulfenylation (also named trifluoromethylthiolation), trifluoromethylsulfinylation, or trifluoromethylsulfonylation reactions. Typically, CF_3_SO_2_Na reacts under oxidative conditions whereas CF_3_SO_2_Cl requires reductive conditions. The advent of a new dynamism and the versatility of these two reagents have been recently demonstrated through several creative research articles; hence, this review aims to collect the recent progresses in the diverse uses of CF_3_SO_2_Na and CF_3_SO_2_Cl reagents. A special emphasis is placed on mechanistic studies. The review is divided in Part 1 and Part 2 that are published back-to-back. The literature is comprehensively covered through July 2017.

## Review

Sodium trifluoromethanesulfinate (alternate names: sodium triflinate, trifluoromethanesulfinic acid sodium salt, Langlois reagent), CAS No. 2926-29-6, MW 156.06, is a stable white solid (mp 350 °C) soluble in water and slightly soluble in acetonitrile, methanol and acetone [13]. Although this reagent was prepared in 1955 by Haszeldine [14] and in 1976 by Roesky [15], it is only in 1991 that Langlois reported the trifluoromethylation of aromatic compounds under oxidative conditions [16]. Since then, the use of CF_3_SO_2_Na has grown considerably for the creation of C_sp3_–CF_3_, C_sp2_–CF_3_ and C_sp_–CF_3_ bonds [17–19]. This reagent is also conveniently used in trifluoromethylsulfinylation and trifluoromethylsulfonylation reactions. More recently, from 2015, CF_3_SO_2_Na has found a new application as source of SCF_3_ for direct trifluoromethylsulfenylation.

### 1 Trifluoromethylation

#### C_sp3_–CF_3_ bond-forming reactions

**Scheme 1:**
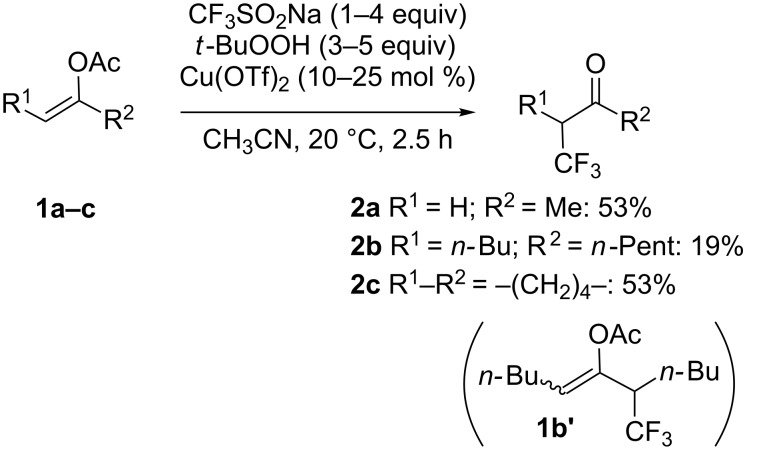
Trifluoromethylation of enol acetates by Langlois.

**Scheme 2:**
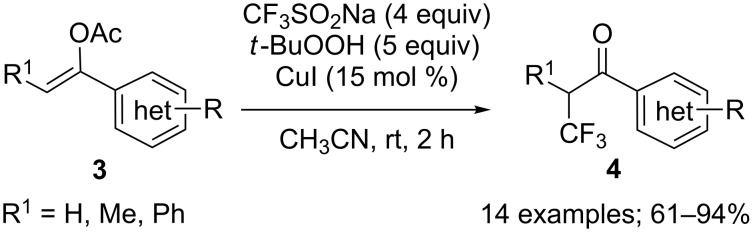
Trifluoromethylation of (het)aryl enol acetates.

**Scheme 3:**
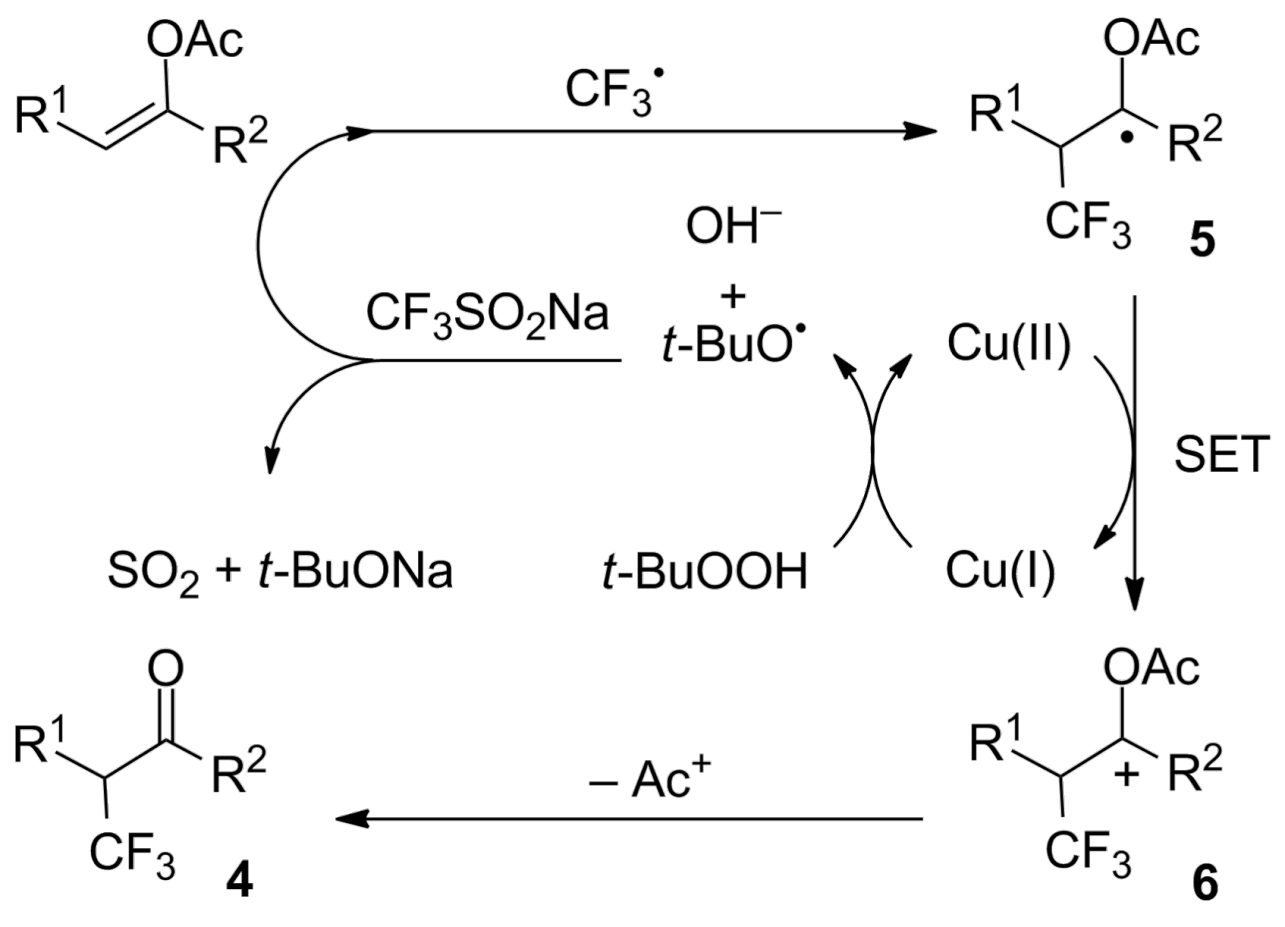
Mechanism for the trifluoromethylation of enol acetates.

**Synthesis of α-trifluoromethyl ketones from alkenes:** After their original reports on the trifluoromethylation of aromatics (see later in the text, Scheme 34) [16] and disulfides (Scheme 69) [20], Langlois and co-workers demonstrated that enol acetates **1a**–**c** were converted into the corresponding α-trifluoromethyl ketones upon treatment with CF_3_SO_2_Na with *tert*-butyl hydroperoxide (TBHP) and a catalytic amount of copper(II) triflate (Scheme 1) [21]. The scope was rather narrow and yields were moderate to poor. In particular, enol acetate **1b**, prepared from symmetrical undecan-6-one, gave a mixture of the desired α-CF_3_ ketone and two isomeric enol acetates **1b'**. In 2014, Li, Duan and co-workers applied the conditions described by Langlois to a series of enol acetates **3** derived from aryl and heteroaryl ketones featuring a single enolizable position (Scheme 2) [22]. Notably, it was found that Cu(II) and Cu(I) salts gave similar yields. A radical trifluoromethylation was suggested for this transformation. The CF_3_^•^, which was generated by reaction of *tert*-butyl hydroperoxide with CF_3_SO_2_Na in the presence of copper(I), reacted at the more electron-rich carbon atom of the C=C double bond to give the radical species **5** that was oxidised by copper(II) into the corresponding cationic intermediate **6** via a single electron transfer (SET). Finally, the acetyl cation was eliminated to provide the α-CF_3_ carbonyl compound **4** (Scheme 3).

**Scheme 4:**
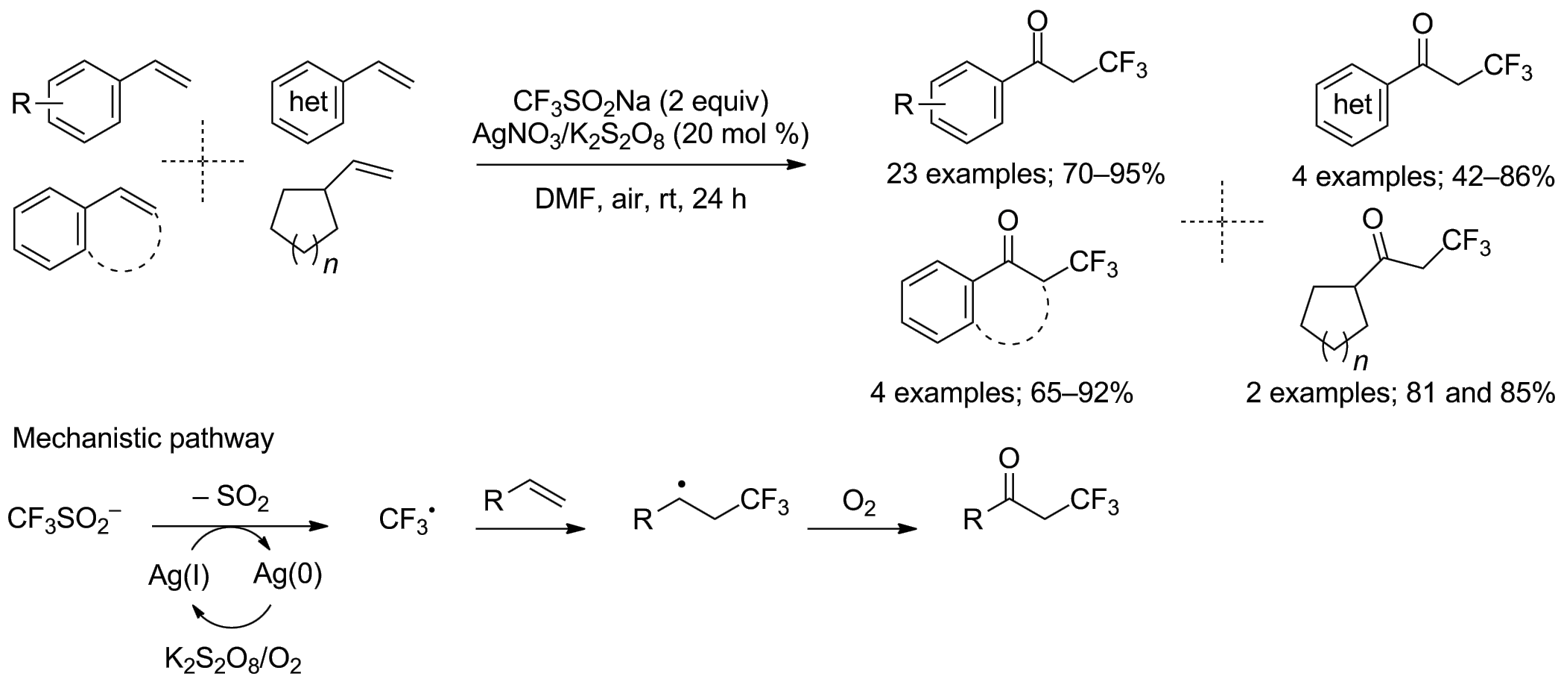
Oxidative trifluoromethylation of unactivated olefins and mechanistic pathway.

Unactivated olefins are widely available substrates prone to be transformed into α-trifluoromethyl carbonyl compounds under oxidative trifluoromethylation as first reported by Maiti and co-workers in 2013. The reaction is operationally simple, conducted under air at room temperature and presents a predictable reactivity pattern as well as a wide functional group tolerance. The substrate scope was evaluated on 33 styrenes, β-substituted styrenes, heteroaromatic olefins and vinyl cycloalkanes (Scheme 4). The trifluoromethyl radical was generated from CF_3_SO_2_Na by means of an oxidative system comprising catalytic amounts of silver(I) nitrate and potassium persulfate K_2_S_2_O_8_. Both atmospheric oxygen and K_2_S_2_O_8_ can be the source of the oxygen atom of the ketone moiety. A series of experiments that include the formation of TEMPO–CF_3_ (TEMPO: 2,2,6,6-tetramethylpiperidine 1-oxyl), the detection of Ag(0) by X-ray photoelectron spectroscopy, the retardation of the reaction in absence of air and an ^18^O-labeling reaction led the authors to propose the mechanism described in Scheme 4 [23].

**Scheme 5:**
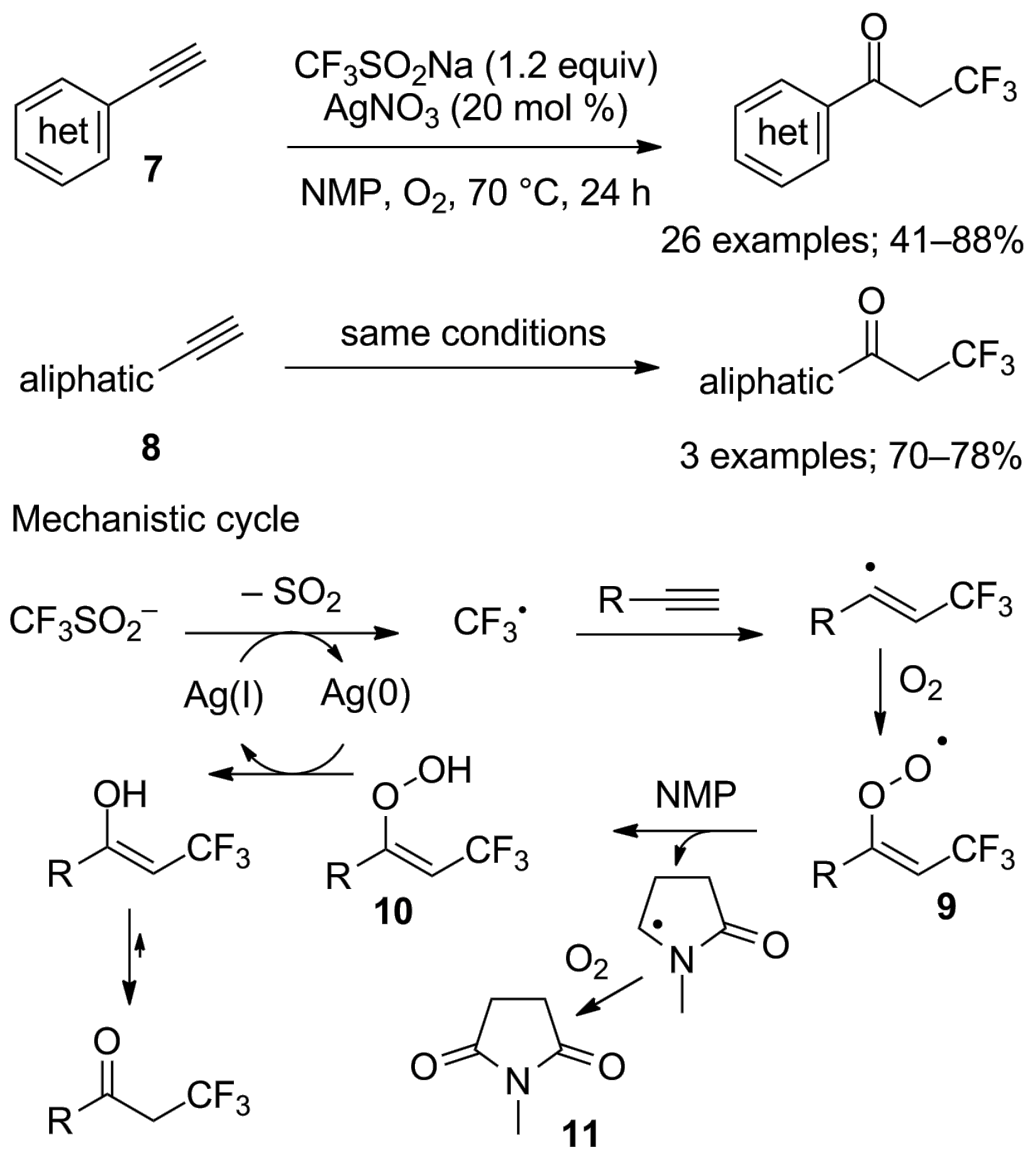
Oxidative trifluoromethylation of acetylenic substrates.

Because some limitations appeared with heterocycles such as quinolone, indole, pyrimidine, thiophene, etc. the Maiti group reported an alternative approach toward α-trifluoromethyl ketones starting from (hetero)arylacetylenes **7** and also aliphatic terminal alkynes **8** (Scheme 5) [24]. The trifluoromethyl radical was generated from CF_3_SO_2_Na as indicated earlier, oxygen from air was the source of the oxygen atom, and *N*-methylpyrrolidine (NMP) acted as solvent and source of the hydrogen atom to convert the peroxo intermediate **9** to its hydroperoxo form **10**. As proof of the mechanism, *N*-methylsuccinimide (**11**) was identified in all these reactions.

**Scheme 6:**
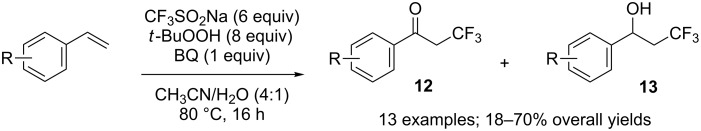
Metal free trifluoromethylation of styrenes.

Simultaneously to Maiti’s work, Luo and co-workers reported a metal-free protocol for the trifluoromethylation of styrenes with CF_3_SO_2_Na, *tert*-butyl hydroperoxide and benzoquinone (BQ) as oxidant. The reactions were run at 80 °C for 16 h to give mixtures of α-trifluoromethyl ketones **12** and the corresponding alcohols **13** (Scheme 6) [25]. The scope was limited to simple substituted styrenes and yields were only moderate although super-stoichiometric amounts of reagents were used. The ratio **12**/**13** ranged from 31:69 to 66:34 but a subsequent reduction or oxidation allowed to access either the alcohol or the ketone exclusively.

**Scheme 7:**
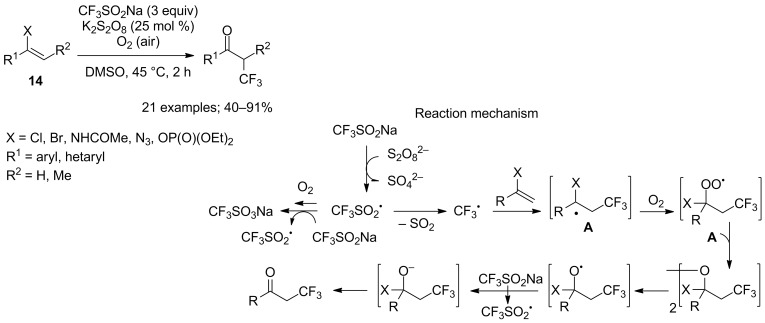
Synthesis of α-trifluoromethylated ketones by oxytrifluoromethylation of heteroatom-functionalised alkenes.

The use of transition metal catalysts and/or a large excess of organic oxidants can be obstacles to production. In a quest for ideal conditions, Lei and co-workers exposed heteroatom-functionalised alkenes **14** to aerobic C_vinyl_–heteroatom bond oxygenation under metal-free conditions, where oxygen from air worked in concert with a catalytic amount of potassium persulfate to activate CF_3_SO_2_Na (Scheme 7) [26]. The heteroatom must be a good leaving group or part of it (X = Br, Cl, NHCOMe, N_3_, OP(O)(OEt)_2_). A mechanistic investigation demonstrated the role of oxygen: 93% isotopic purity of the ketone product was obtained when using ^18^O_2_; no reaction occurred under N_2_ instead of O_2_; K_2_S_2_O_8_ rather than O_2_ served as initiator; the radical CF_3_SO_2_^•^ could either extrude SO_2_ to CF_3_^•^ or react with O_2_ to re-initiate the radical chain process (Scheme 7).

**Scheme 8:**
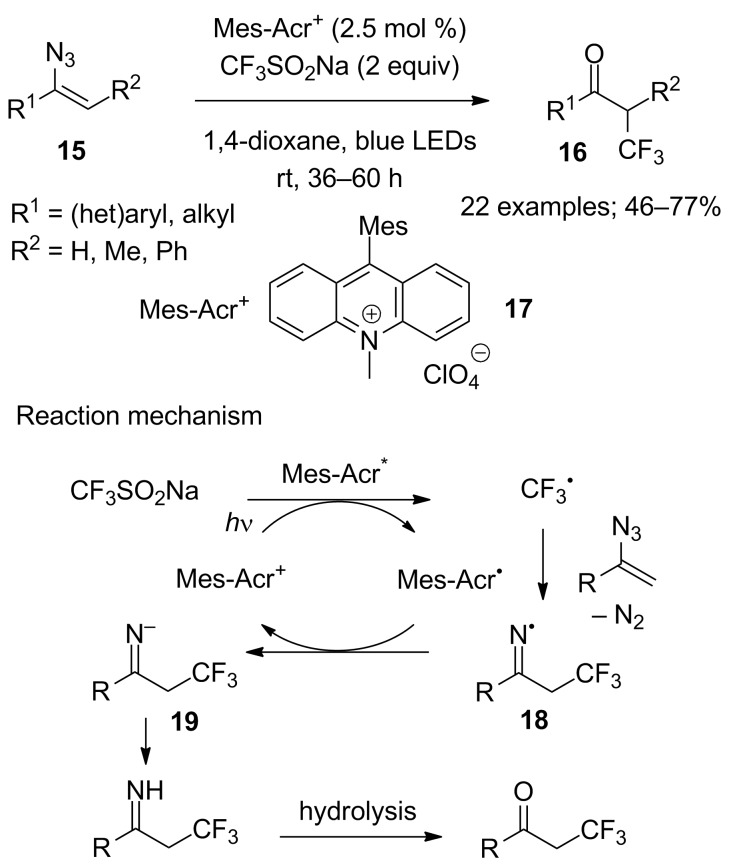
Catalysed photoredox trifluoromethylation of vinyl azides.

Vinyl azides were used by Liu and co-workers as precursors of α-trifluoromethylated ketones by reaction of CF_3_SO_2_Na under photoredox catalysis. The substrate scope was broad and the reaction proceeded with high functional group tolerance; indeed, aryl-, alkyl-, hetero-functionalised terminal as well as non-terminal vinyl azides **15** were compatible with the reaction conditions. In the presence of the organic photocatalyst *N*-methyl-9-mesitylacridinium (**17**), CF_3_SO_2_Na was converted into CF_3_^•^ upon visible-light irradiation. The CF_3_^•^ radical reacted with the vinyl azide to give the iminyl radical **18** that was reduced by Mes-Acr^•^ (Mes-Acr: 9-mesityl-10-methylacridinium) into the iminyl anion **19**. After protonation and hydrolysis of the imine function, the α-trifluoromethylated ketones **16** were obtained in moderate to good yields (Scheme 8) [27].

**Scheme 9:**
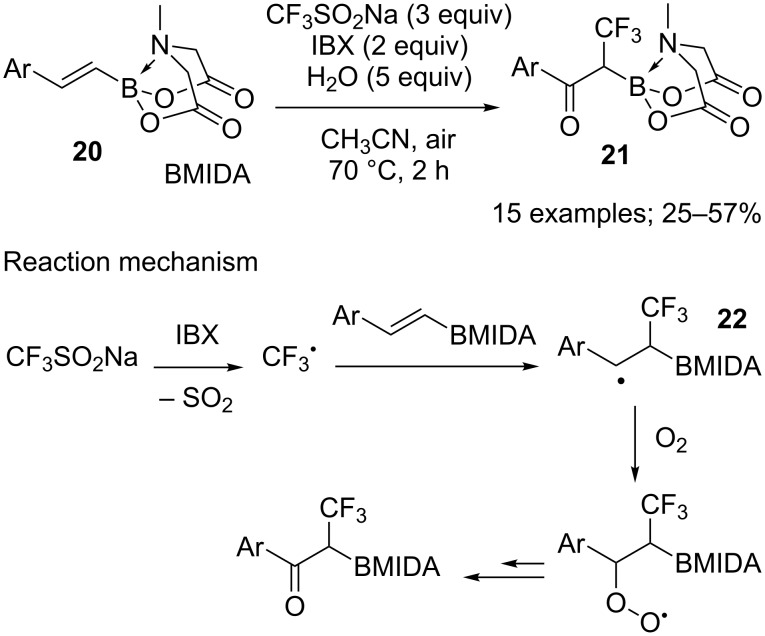
Oxidative difunctionalisation of alkenyl MIDA boronates.

Alkenyl MIDA (*N*-methylimidodiacetic) boronates **20**, as functionalised alkenes, were transformed into α-trifluoromethyl-α-boryl ketones **21** by oxidative trifluoromethylation with CF_3_SO_2_Na. In that case, 2-iodoxybenzoic acid (IBX) was used as the oxidant to generate the trifluoromethylated radical **22** and atmospheric oxygen was the oxygen source to form the ketone (Scheme 9) [28].

**Scheme 10:**
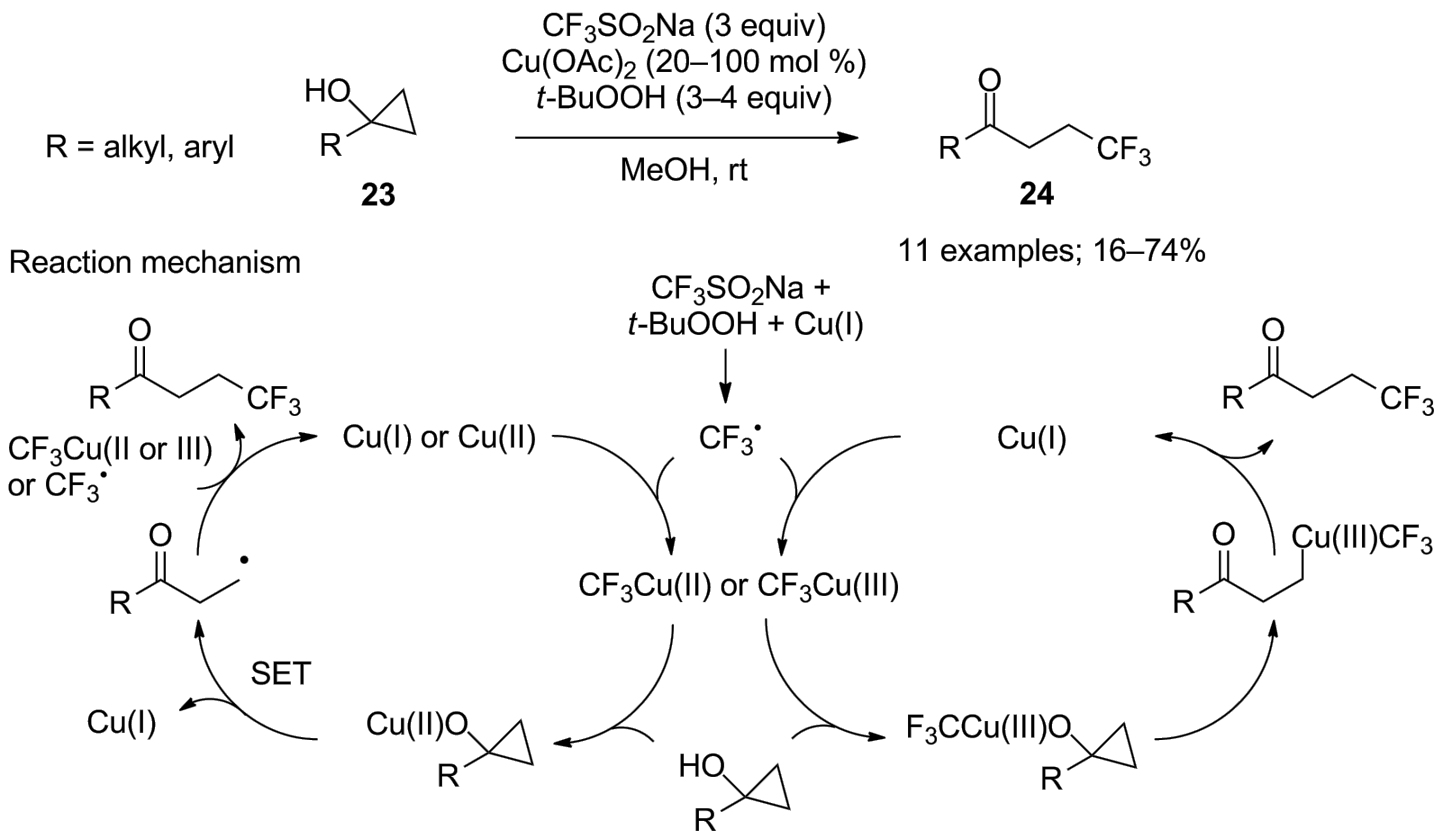
Synthesis of β-trifluoromethyl ketones from cyclopropanols.

**Synthesis of β-trifluoromethyl ketones from cyclopropanols:** Moving away the CF_3_ group by one carbon atom further from the carbonyl function would provide the corresponding β-trifluoromethyl ketones. For this aim, distally trifluoromethyl ketones were synthesised by the ring-opening of cyclopropanol derivatives by means of per- or polyfluorinated sulfonates, including CF_3_SO_2_Na. Kananovich and co-workers demonstrated that sodium triflinate, under Langlois’ conditions, served as a precursor of trifluoromethyl copper species that were involved in the ring-opening trifluoromethylation of tertiary cyclopropanols **23** (Scheme 10) [29]. The substrate scope was broad allowing access to various β-trifluoromethyl ketones **24** featuring aryl, alkyl, functionalised alkyl, alkenyl, acetal, silylated alcohol, pyran, and piperidine functionalities (R group in **24**). Mechanistic studies by ^19^F NMR allowed to identify CF_3_ complexes of copper(I) and copper(III) and the predominance of an electrophilic pathway (versus SET pathway) was suggested by regioselective trifluoromethylation of a case substrate. Although Cu(II) acetate was used, the authors mentioned that Cu(I) can be formed in situ by reduction with MeOH or the cyclopropanol. Concomitant mechanisms are depicted in Scheme 10.

**Scheme 11:**
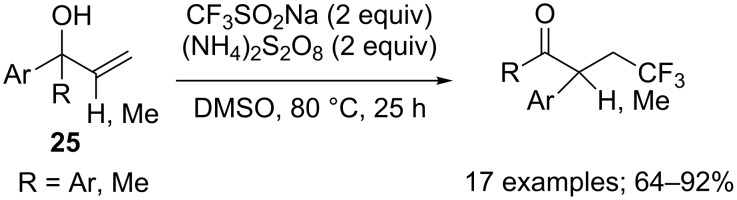
Aryltrifluoromethylation of allylic alcohols.

β-Trifluoromethyl ketones could also be obtained from allylic alcohols **25** by a cascade trifluoromethylation/1,2-aryl migration. Yang, Xia and co-workers employed sodium triflinate under metal-free conditions with ammonium persulfate as the oxidant that was necessary to generate the CF_3_ radical (Scheme 11) [30].

**Scheme 12:**
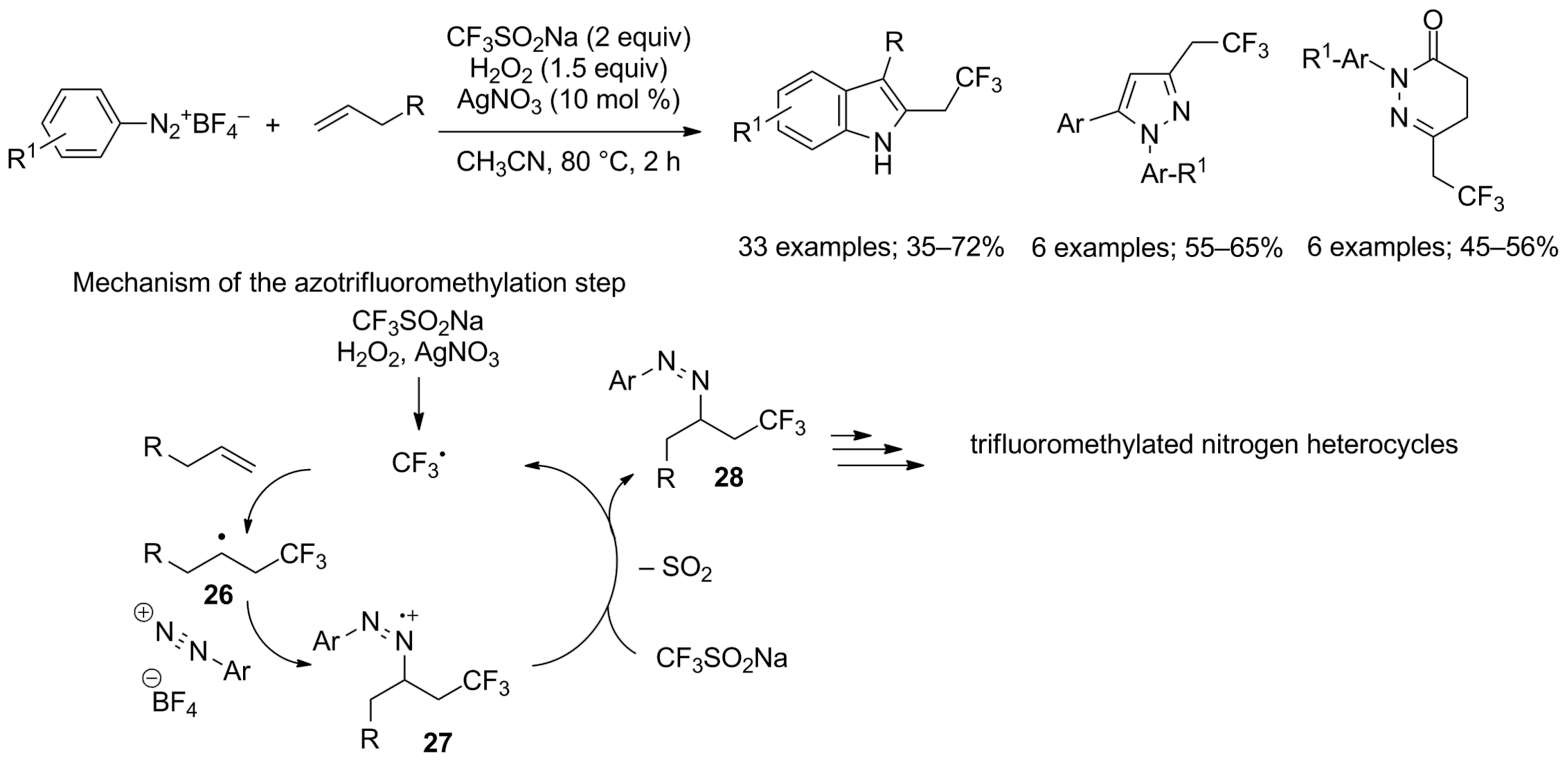
Cascade multicomponent synthesis of nitrogen heterocycles via azotrifluoromethylation of alkenes.

**Amino- and azotrifluoromethylation of alkenes:** Alkene trifluoromethylation was applied to the construction of indole, pyrazole and pyridazinone moieties via a multicomponent cascade reaction developed by Antonchick and Matcha in 2014 [31]. The method was based on the reaction between simple alkenes, sodium triflinate and diazonium salts. The CF_3_ radical was produced from CF_3_SO_2_Na by oxidation with H_2_O_2_ in the presence of silver nitrate. Then, CF_3_^•^ was added to the terminal position of the alkene to give radical **26** that was trapped by the arenediazonium salt to form the radical cation **27**, which was reduced into **28** prior to be converted into nitrogen heterocycles via [1,3]-hydride shift and cyclisation steps (Scheme 12). The reaction was regioselective and had a broad scope. This application of alkene trifluoromethylation provided a convenient entry to trifluoromethylated nitrogen heterocycles.

**Scheme 13:**
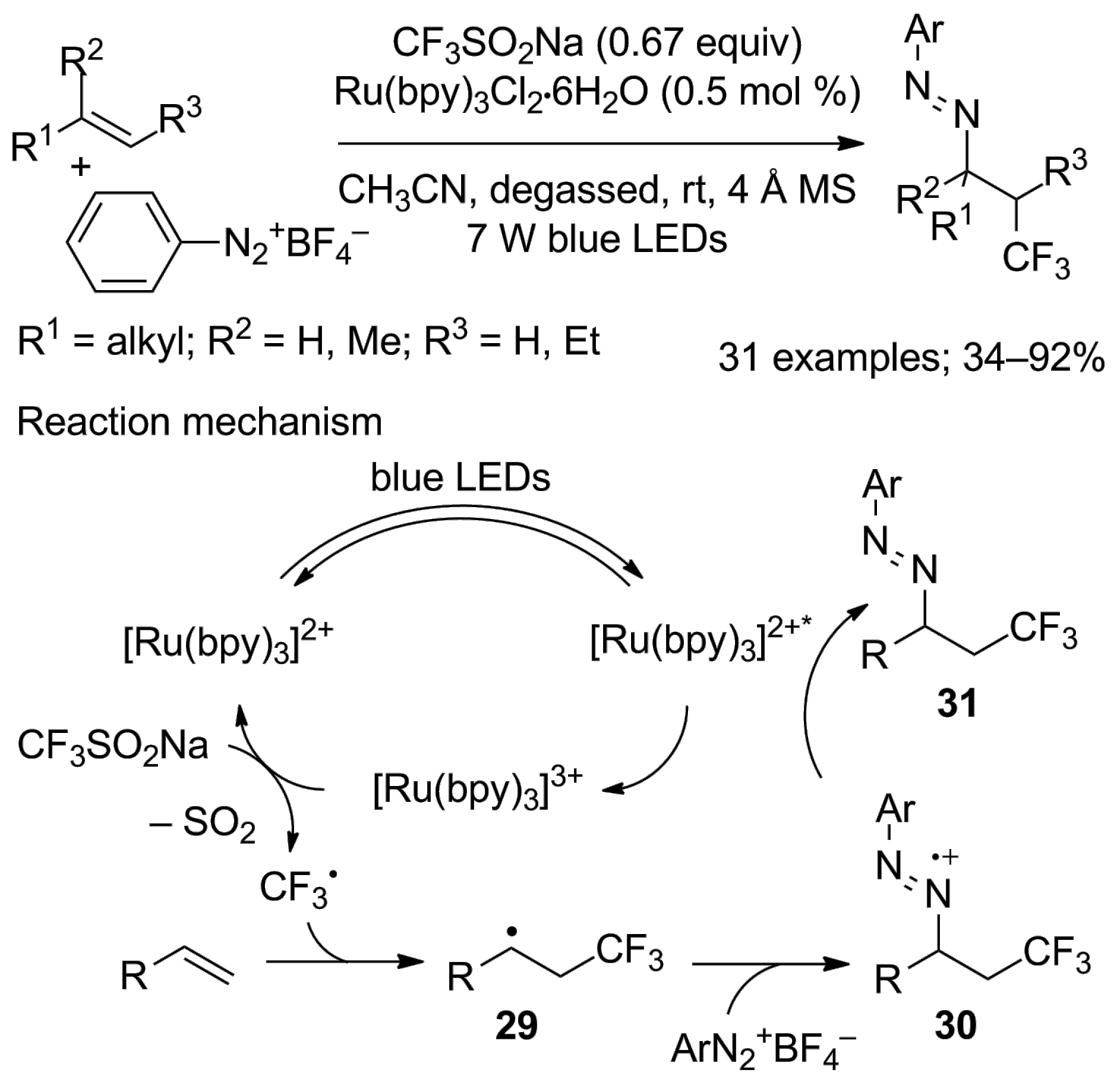
Photocatalytic azotrifluoromethylation of alkenes with aryldiazonium salts and CF_3_SO_2_Na.

Subsequently, a photoredox-catalysed azotrifluoromethylation of unactivated alkenes was developed by Chen, Xiao and co-workers [32]. The scope was broad for a variety of unactivated alkenes, functionalised or not, and aryldiazonium salts (Scheme 13). Interestingly, the method did not require stoichiometric amounts of oxidant and further transformation of the azotrifluoromethyl products allowed a Fisher indole synthesis. From a mechanistic point of view, the excited photocatalyst was oxidised by the aryldiazonium salt to produce [Ru(bpy)_3_]^3+^ (bpy: 2,2’-bipyridine) as the oxidant to generate the CF_3_ radical from CF_3_SO_2_Na with extrusion of SO_2_. Then, CF_3_^•^ underwent a radical addition to the alkene to form the radical **29**, which was trapped by the aryldiazonium salt to give the radical cation **30**. Finally, **30** was reduced by [Ru(bpy)_3_]^2+*^ to end up with the product **31** (Scheme 13).

**Scheme 14:**
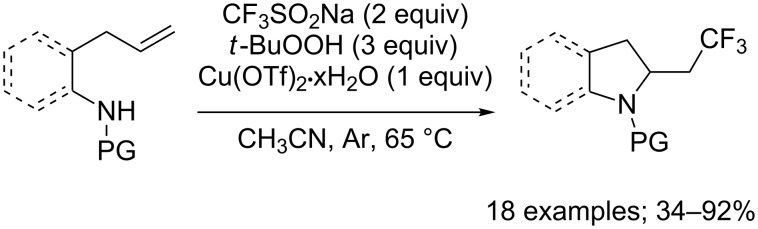
Copper-promoted intramolecular aminotrifluoromethylation of alkenes with CF_3_SO_2_Na.

The aminotrifluoromethylation of alkenes in an intramolecular version was reported by Zhang and co-workers in 2017 (Scheme 14) [33]. Langlois’ conditions with *tert*-butyl hydroperoxide and a catalytic amount of copper(II) triflate were used to prepare a series of CF_3_-containing indoline, pyrrolidine, lactam and lactone.

**Scheme 15:**
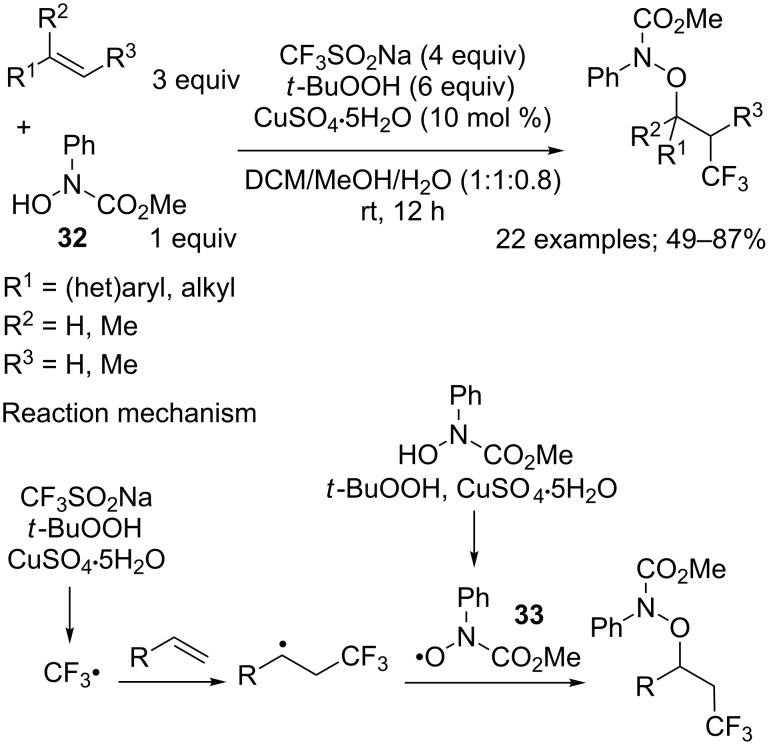
Oxytrifluoromethylation of alkenes with CF_3_SO_2_Na and hydroxamic acid.

**Oxytrifluoromethylation of alkenes:** The difunctionalisation of alkenes including a trifluoromethylation step was extended to carbon–oxygen bond formation via oxytrifluoromethylation. We have already described some examples of such a reaction leading to α-trifluoromethyl ketones (vide supra) [26]. Now synthetic routes are presented leading to vicinal trifluoromethyl alcohols. In 2013 Qing and Jiang described the oxytrifluoromethylation of alkenes with hydroxamic acids **32** and CF_3_SO_2_Na under Langlois’ conditions with the couple *t*-BuOOH/copper salt (Scheme 15) [34]. A competitive formation of two radicals, CF_3_^•^ and the amidoxyl radical [ArN(CO_2_Me)O^•^] **33**, would lead to two regioisomeric oxytrifluoromethylated products. Fortunately, this issue was solved by the primary formation of the CF_3_ radical and thus a regioselective addition. After optimization of the reaction conditions with styrene as model alkene, the method was applied to a wide range of alkenes featuring various functional groups. Further reduction of the N–O bond by Mo(CO)_6_ gave the corresponding alcohols.

**Scheme 16:**
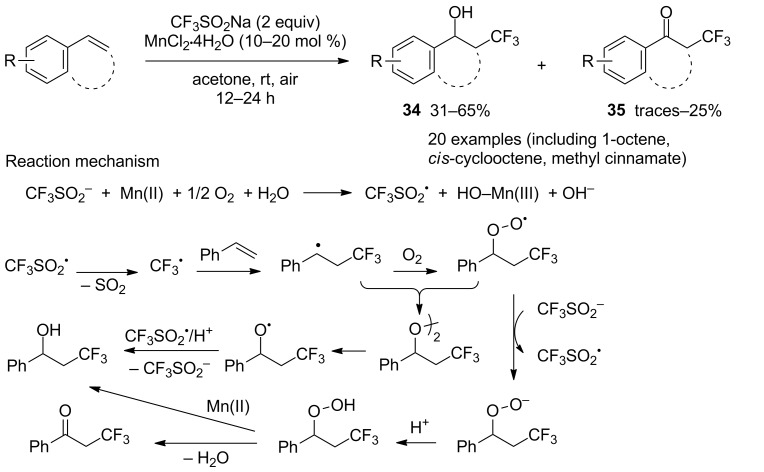
Manganese-catalysed oxytrifluoromethylation of styrene derivatives.

A protocol free of peroxide initiator was developed by Yang, Vicic and co-workers using a manganese salt and O_2_ from air [35]. Styrene derivatives were transformed preferentially into hydroxytrifluoromethylated compounds **34** versus the corresponding ketones **35** in moderate to good selectivities (Scheme 16). In the case of 1,2-disubstituted alkenes, mixtures of *syn-* and *anti-*isomers were obtained. A radical pathway was supported by several observations: (i) addition of TEMPO suppressed the reaction; (ii) an induction period was observed followed by acceleration with consumption of styrene; (iii) vinyl triflone was detected indicating the formation of CF_3_SO_2_^•^; (iv) formation of CF_3_SO_3_^–^ via oxidation of CF_3_SO_2_^•^ (Scheme 16).

**Scheme 17:**
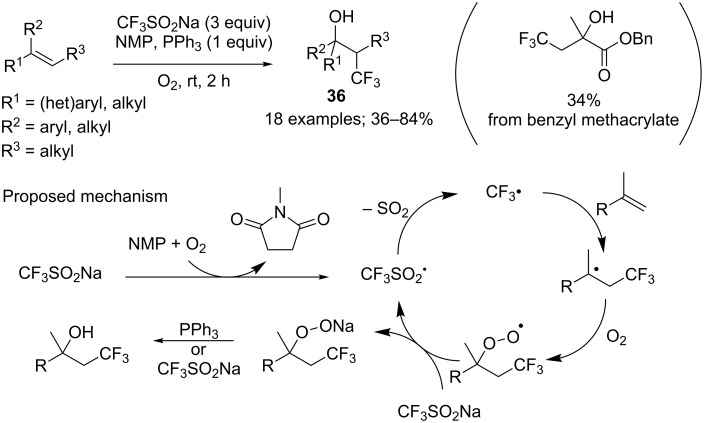
Oxytrifluoromethylation of alkenes with NMP/O_2_ and CF_3_SO_2_Na.

A metal-free approach with in situ generation of the peroxide from the combination of NMP and O_2_ as the radical initiator was proposed by Lei and co-workers [36]. This method was based on a previous work by Maiti (see Scheme 5) [24] but did not require a metal to generate the CF_3_ radical. Tertiary β-trifluoromethyl alcohols **36** were obtained in good yields from a variety of di- and trisubstituted alkenes (Scheme 17). Labelling and IR experiments were conducted to investigate the reaction mechanism as well as kinetic studies that revealed the reaction rate dependence on O_2_ diffusion.

**Scheme 18:**
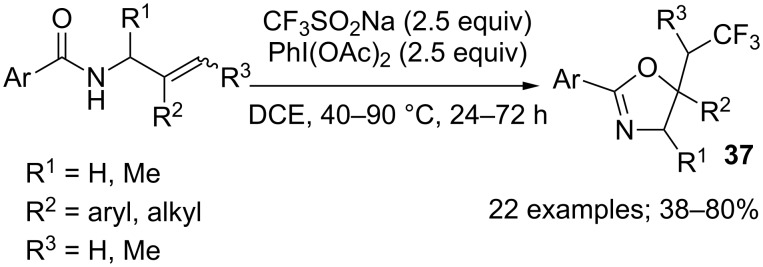
Intramolecular oxytrifluoromethylation of alkenes.

A case of intramolecular oxytrifluoromethylation of alkenes leading to oxazolines **37** was described by Fu and co-workers in 2014 [37]. In this work iodobenzene diacetate (PIDA) was used as the oxidant to generate the CF_3_ radical from CF_3_SO_2_Na (Scheme 18).

**Scheme 19:**
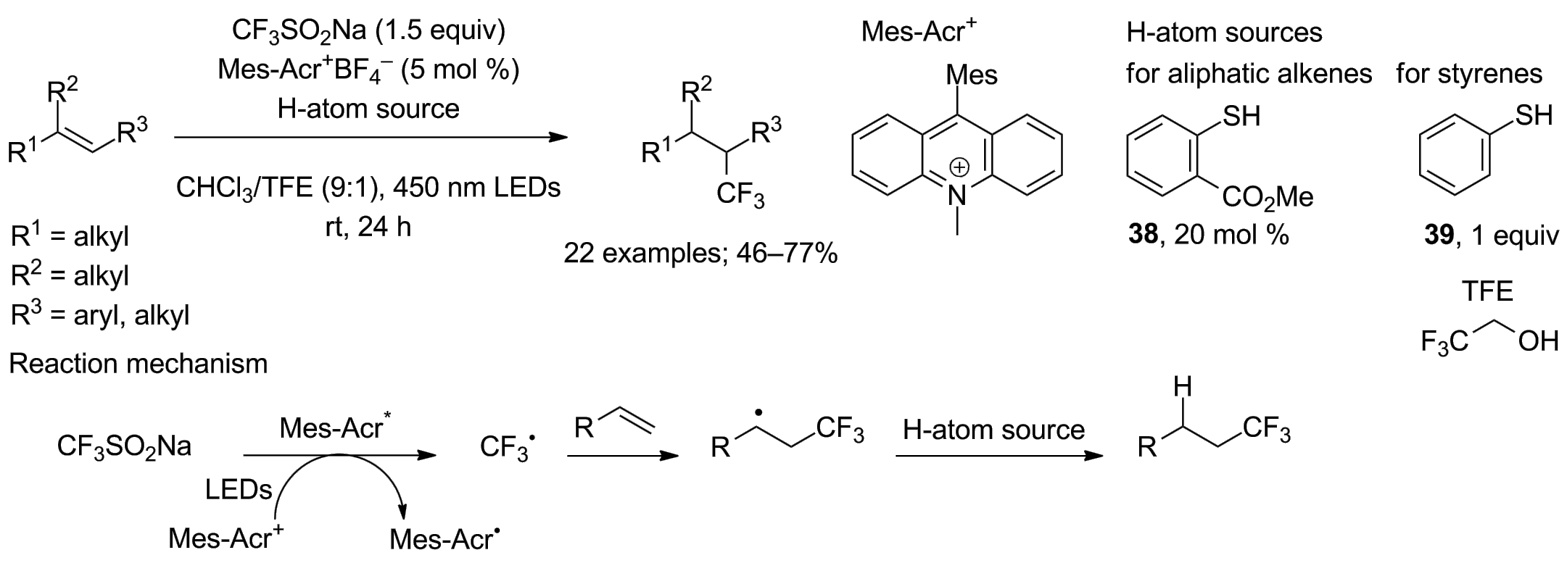
Hydrotrifluoromethylation of styrenyl alkenes and unactivated aliphatic alkenes.

**Hydrotrifluoromethylation of alkenes:** Direct alkene hydrotrifluoromethylation by means of CF_3_SO_2_K under electrochemical oxidation was first reported by Tommasino and co-workers in 2002 on three alkenes but yields were below 20% due to the formation of oxidised byproducts [38]. Nicewicz and co-workers in 2013 found suitable reaction conditions for the alkene hydrotrifluoromethylation using CF_3_SO_2_Na [39]. The single electron oxidation of CF_3_SO_2_Na was performed by visible-light activated *N*-methyl-9-mesitylacridinium as a photoredox catalyst. Two hydrogen atom donors, 20 mol % of methyl thiosalicylate **38** for aliphatic alkenes (or 1 equiv of thiophenol **39** for styrenyl alkenes) and 2,2,2-trifluoroethanol (TFE), worked in concert for the hydrogen atom transfer with complete suppression of the oxidised trifluoromethylated byproducts. The method was regioselective for mono-, di-, and trisubstituted aliphatic alkenes and styrenyl alkenes with a broad substrate scope (Scheme 19). As an exception, 1,2-disubstituted alkenes and chalcone gave low regioselectivities with mixed Markovnikov and anti-Markovnikov products.

**Scheme 20:**
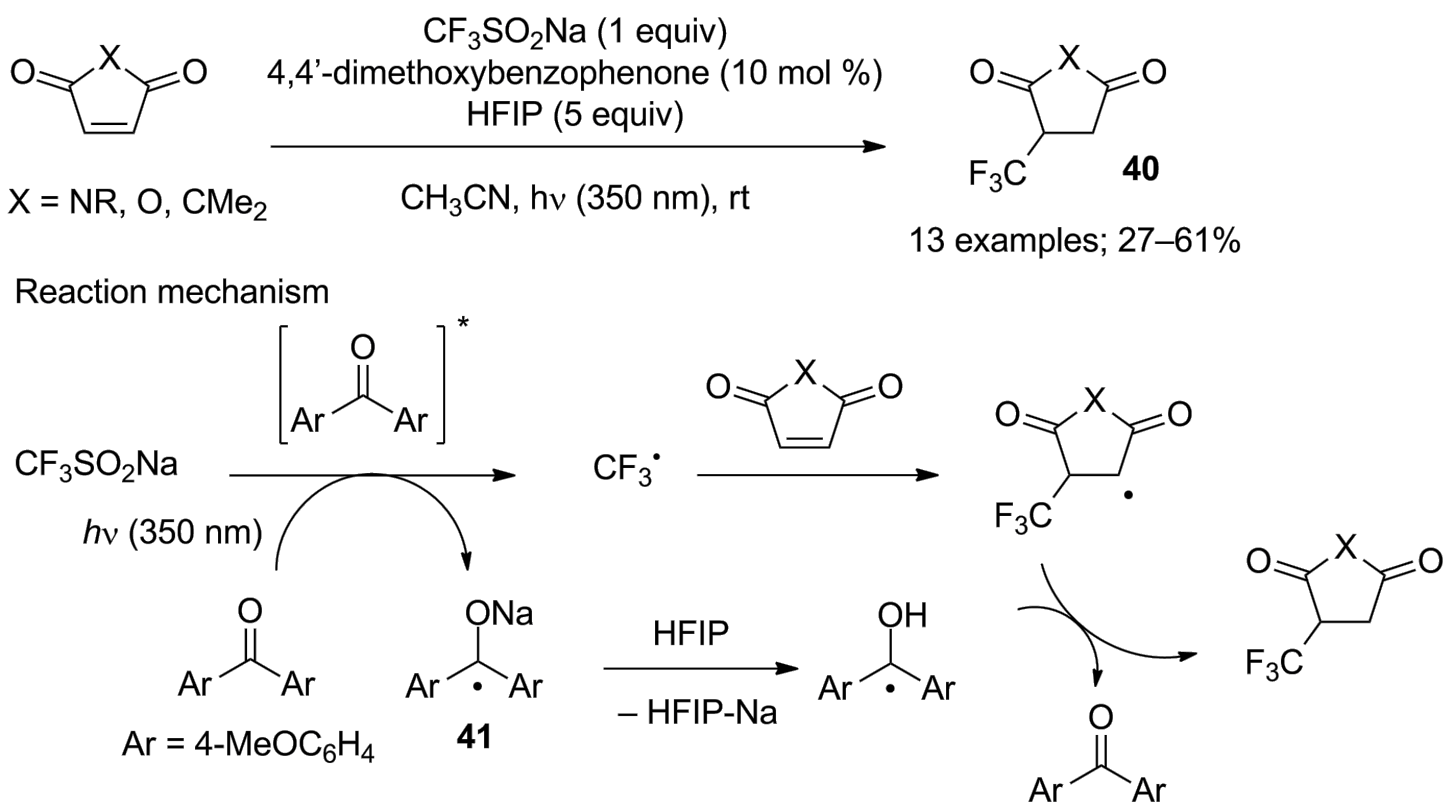
Hydrotrifluoromethylation of electron-deficient alkenes.

The CF_3_ radical is electrophilic in nature and, as such, not prone to readily react with electron-deficient alkenes. Nevertheless, Lefebvre, Hoffmann and Rueping reported that *N*-substituted maleimides, maleic anhydride and dimethyl maleate were hydrotrifluoromethylated with CF_3_SO_2_Na in the presence of 4,4’-dimethoxybenzophenone as photosensitiser under near-UV irradiation (350 nm) and hexafluoroisopropanol (HFIP) as a proton donor (Scheme 20) [40]. The reactions were performed in batch and under continuous flow conditions with rate enhancement for the latter setup. It was proposed that the CF_3_ radical added onto the substrate while the ketyl radical **41** was protonated by HFIP. Then, hydrogen transfer gave the hydrotrifluoromethylated product **40** and the sensitiser was regenerated (Scheme 20). In the same paper, the authors also realised the same chemical transformation under visible light irradiation at 450 nm by means of the iridium photocatalyst Ir[dF(CF_3_)ppy]_2_(dtbbpy)PF_6_ ([4,4’-bis(*tert*-butyl)-2,2’-bipyridine]bis[3,5-difluoro-2-[5-(trifluoromethyl)-2-pyridinyl]phenyl]iridium(III) hexafluorophosphate), which delivered comparable and even higher yields of the products in longer reaction times but with lower catalyst loading (1 mol %).

**Scheme 21:**
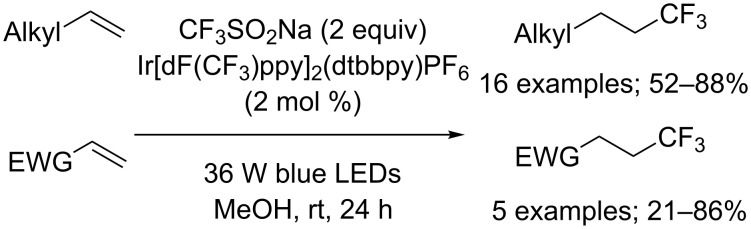
Hydrotrifluoromethylation of alkenes by iridium photoredox catalysis.

Both unactivated terminal alkenes and electron-deficient alkenes (Michael acceptors) were successfully hydrotrifluoromethylated under irradiation with 36 W blue LEDs in the presence of an iridium photoredox catalyst as reported by Zhu, Zhang and co-workers [41]. Of the photocatalysts tested, Ir[dF(CF_3_)ppy]_2_(dtbbpy)PF_6_ had appropriate redox potentials and gave the best results. A wide range of terminal alkenes featuring several functional groups reacted with exclusive anti-Markovnikov selectivity. Notably, styrene failed to react under these conditions. A selection of α,β-unsaturated electron-withdrawing motifs that included a sulfone, esters, an amide, and a ketone were investigated for the first time and the β-addition products were obtained regioselectively in moderate to good yields (Scheme 21). It was suggested that the methylene radical formed by addition of the CF_3_ radical onto the alkene was reduced by the sulfinate anion and the corresponding carbanion was protonated by methanol.

**Scheme 22:**
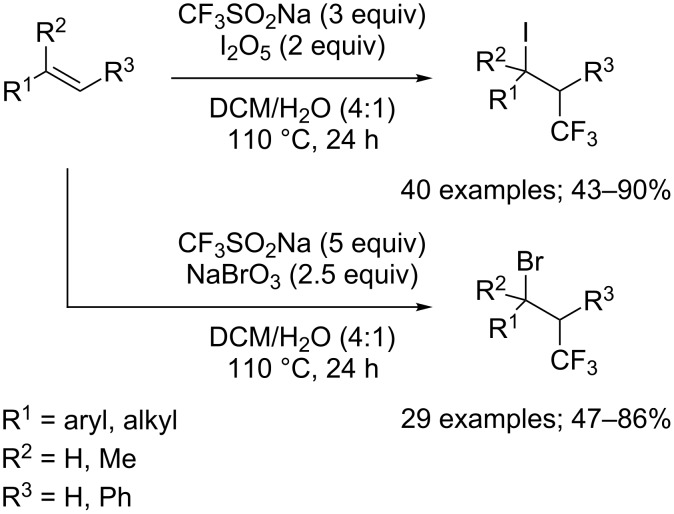
Iodo- and bromotrifluoromethylation of alkenes by CF_3_SO_2_Na/I_2_O_5_ or CF_3_SO_2_Na / NaBrO_3_.

**Halo- and pseudohalotrifluoromethylation of alkenes:** The direct iodotrifluoromethylation was previously achieved by means of gaseous CF_3_I until Liu and co-workers reported the convenient use of CF_3_SO_2_Na and iodide pentoxide, I_2_O_5_, in combination for the iodotrifluoromethylation of alkenes and alkynes (see later in the text) [42]. After an optimisation with 4-chlorostyrene, the reaction was developed with a wide range of terminal and internal alkenes bearing diverse functional groups such as halogens, nitro, sulfonate, sulfamide, carboxylate, amide, ether, carbonyl, and hydroxy that were all well-tolerated (Scheme 22). A mixed solvent system of dichloromethane and water was used in a sealed tube at 110 °C. Mechanistic studies by electron spin resonance were carried out in which both the CF_3_^•^ and the β-CF_3_ alkyl radical intermediate were observed by using 2-methyl-2-nitrosopropane as a radical spin trap. A single-electron oxidative free-radical process was clearly ascertained. For the iodination step, the authors proposed that the β-CF_3_ alkyl radical was intercepted by I_2_, which was formed by a multistep redox process from I_2_O_5_. In continuation of this work, the same research group described the bromotrifluoromethylation of alkenes under similar reaction conditions but using sodium bromate, NaBrO_3_, as a bromine source (Scheme 22) [43].

**Scheme 23:**
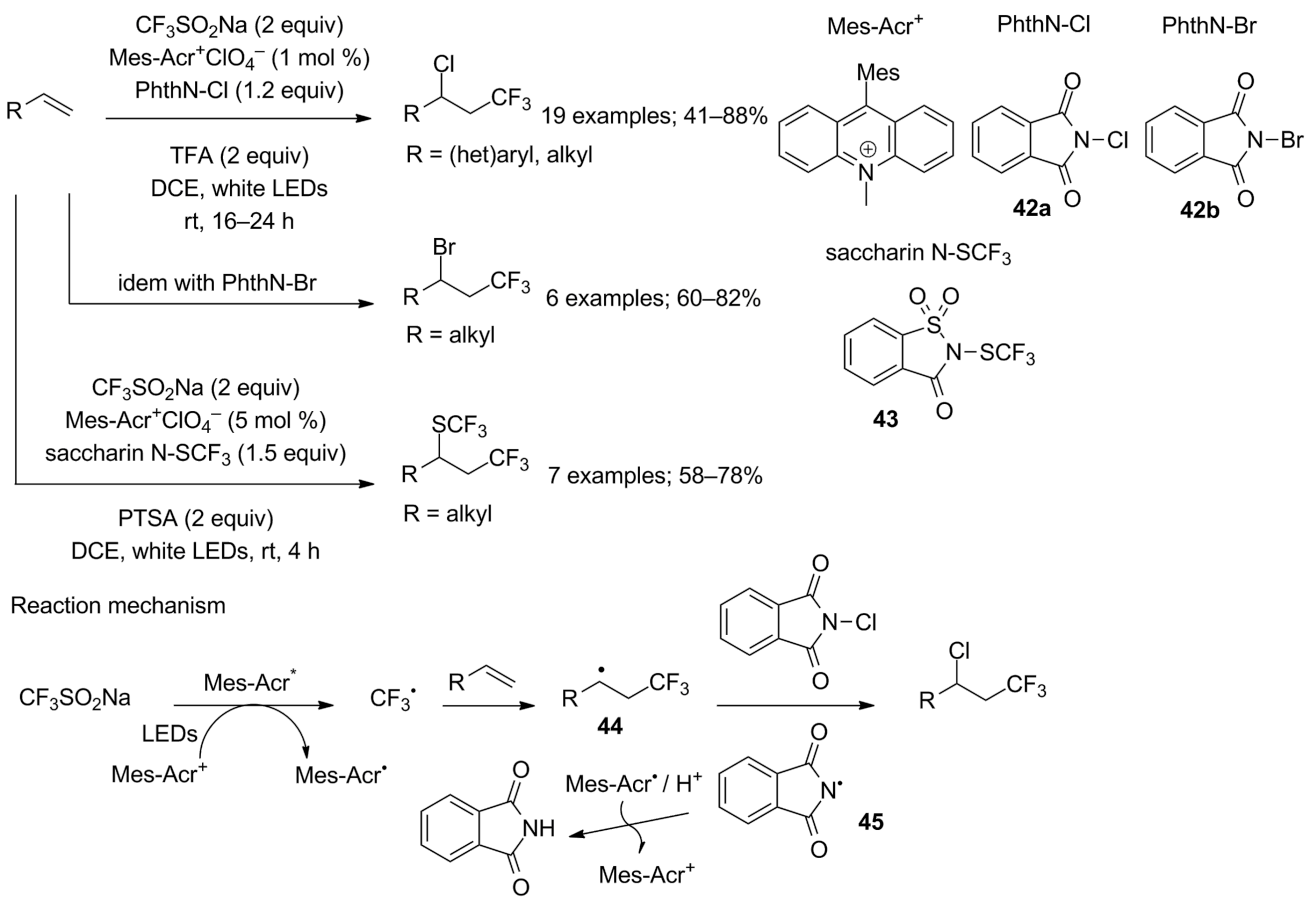
*N*-methyl-9-mesityl acridinium and visible-light-induced chloro-, bromo- and SCF_3_ trifluoromethylation of alkenes.

The photoredox-catalysed chloro-, bromo- and also (trifluoromethylthio)trifluoromethylation of unactivated alkenes was studied by Liu and co-workers in 2017 (Scheme 23) [44]. The Langlois reagent was combined with *N*-halophthalimides **42a**,**b** or *N*-trifluoromethylthiosaccharin **43** in the presence of *N*-methyl-9-mesitylacridinium under visible light irradiation at room temperature. Terminal, internal, and *gem*-substituted alkenes bearing imide, ester, amide, ketone, aldehyde and electron-rich aryl functional groups were suitable substrates. Notably, diethyl 2,2-diallylmalonate as a diene gave the cyclised product resulting of a radical cascade. It has to be noticed that the reactions were conducted in the presence of 2 equivalents of trifluoroacetic or *p*-toluenesulfonic acid; yet, there was no mention of hydrotrifluoromethylated side-products. The mechanism was similar to previous examples to generate the β-CF_3_ alkyl radical intermediate **44**, which was trapped by halogen atom transfer from the halogenating agent. The nitrogen-centered radical **45** oxidised Mes-Acr* by a single-electron-transfer process to restart the catalytic cycle (Scheme 23).

**Carbotrifluoromethylation of alkenes:** The strategies for carbotrifluoromethylation of alkenes with CF_3_SO_2_Na are very much based on methods described earlier in the text: (i) reactions mediated by *tert*-butyl hydroperoxide and a catalytic amount of copper; (ii) metal-catalysed or metal-free reactions with K_2_S_2_O_8_, I_2_O_5_ or a hypervalent iodine reagent, and (iii) photochemical activation. Most of the works concerned cascade intramolecular reactions in which a C–C bond is formed after the initial trifluoromethylation.

**Scheme 24:**
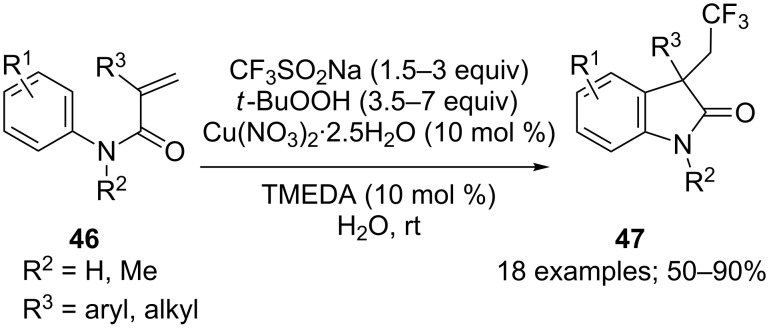
Carbotrifluoromethylation of *N*-arylacrylamides with CF_3_SO_2_Na / TBHP by Lipshutz.

Therefore, Lipshutz and co-workers reported a copper-catalysed intramolecular carbotrifluoromethylation of *N*-arylacrylamides **46** with CF_3_SO_2_Na to produce oxindoles **47** [45]. Addition of the CF_3_ radical to such an electron-deficient alkene should be unfavourable. However, the subsequent annulation step drove the cascade process toward oxindole synthesis. The reaction utilised Langlois’ conditions with *tert*-butyl hydroperoxide and a catalytic amount of Cu(II), but with 10 mol % of tetramethylethylenediamine (TMEDA). Organic solvents were replaced by pure water and the aqueous medium can be recycled up to five times. The substrate scope was large when tertiary amides were used. A secondary arylamide failed to give the expected product. With a substituent at the *meta*-position of the aniline ring, a mixture of regioisomers was obtained. Various alkenes with substituents (R^3^) were investigated and the oxindoles were obtained in moderate to high yields (Scheme 24).

**Scheme 25:**
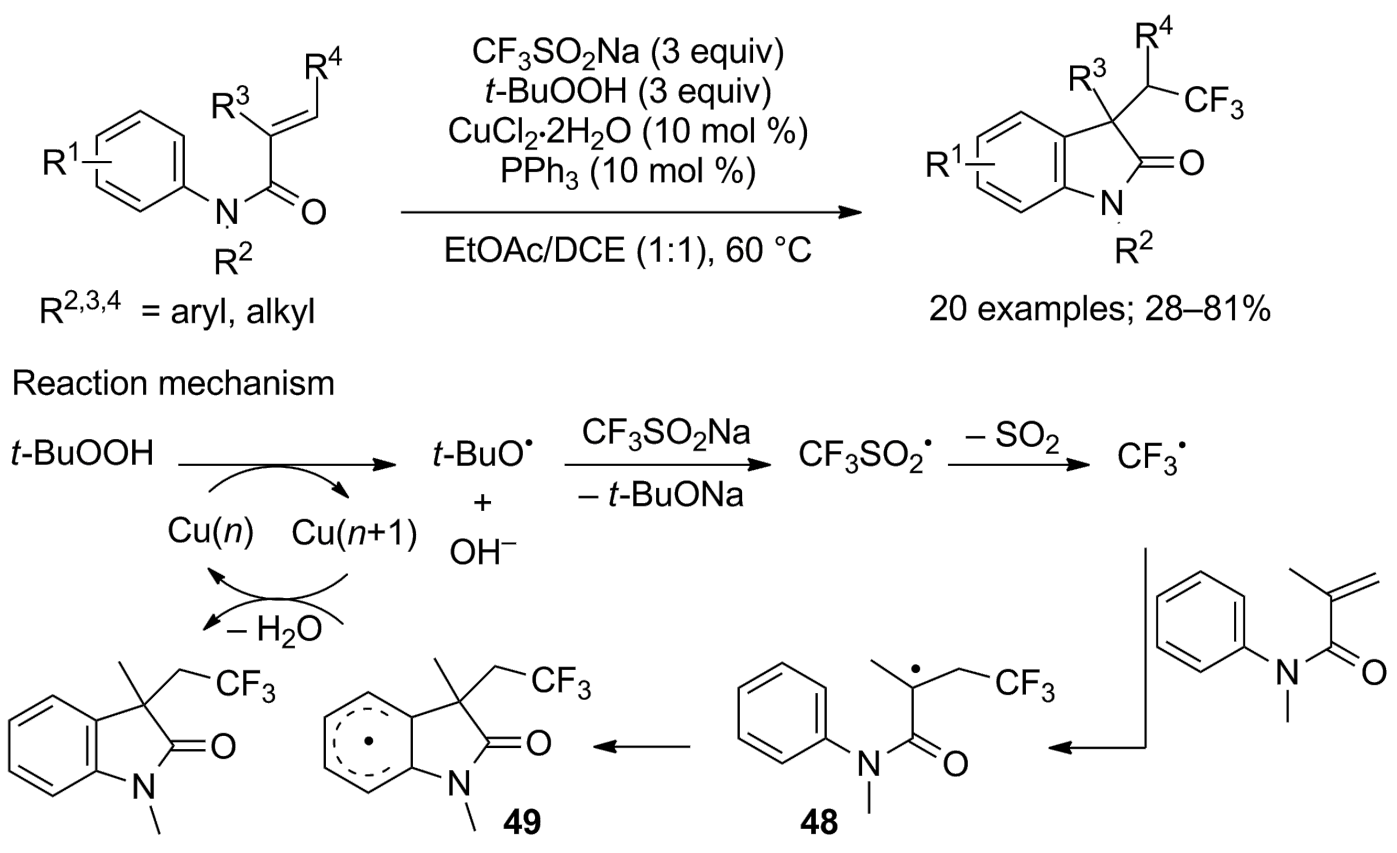
Carbotrifluoromethylation of *N*-arylacrylamides with CF_3_SO_2_Na/TBHP reported by Lei.

Simultaneously, Lei and co-workers published the same reaction under slightly different conditions [46]. They used a combination CF_3_SO_2_Na/TBHP in the presence of catalytic amounts of copper chloride and triphenylphosphine. Trisubstituted alkenes (R^3^ and R^4^ ≠ H) were employed as substrates and diastereoisomers were obtained. The *tert*-butoxyl radical was generated from TBHP and Cu(*n*) via a SET process, which then, it reacted with CF_3_SO_2_Na to liberate CF_3_^•^. The subsequent addition of CF_3_^•^ to the β-position of the C=C bond of the acrylamide gave the intermediate **48**, which underwent an intramolecular radical annulation to produce the aryl radical **49**. Finally, oxidation of **49** by Cu(*n +* 1) and aromatisation afforded the oxindole and regenerated the copper catalyst (Scheme 25).

**Scheme 26:**
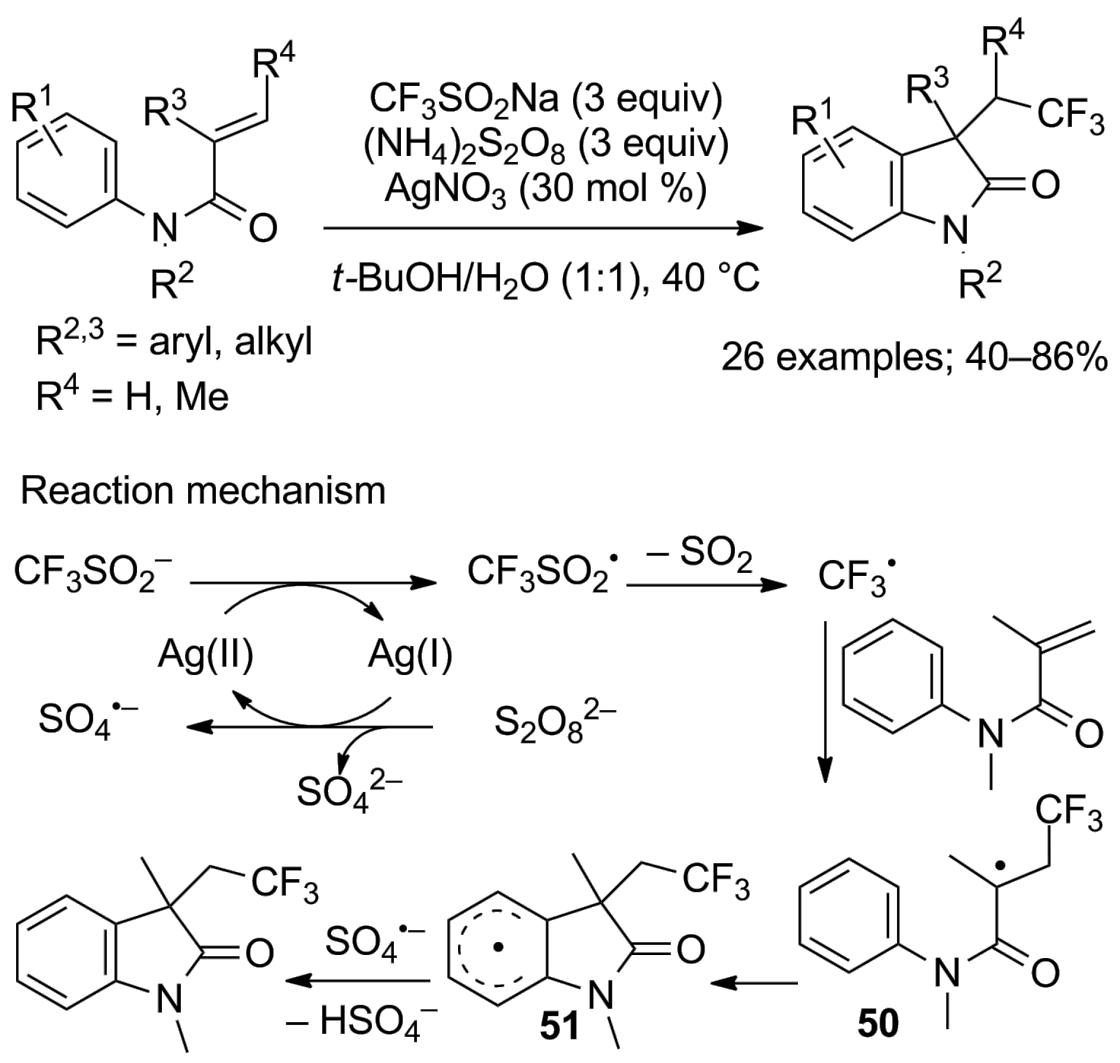
Carbotrifluoromethylation of *N*-arylacrylamides with CF_3_SO_2_Na/(NH_4_)_2_S_2_O_8_.

The same indoles bearing a 2,2,2-trifluoroethyl side-chain were also obtained in reactions performed with CF_3_SO_2_Na and (NH_4_)_2_S_2_O_8_ as the oxidant in the presence of a catalytic amount of AgNO_3_ as reported by Tan and co-workers (Scheme 26) [47]. In the absence of AgNO_3_ the reaction did not work. Notably, *N*-alkyl and *N*-aryl protected substrates worked well, whereas *N*-acyl and *N*–H derivatives failed to deliver the desired products. Mechanistically, Ag(I) was initially oxidised to Ag(II) by the persulfate anion; then, CF_3_SO_2_^–^ was oxidised to CF_3_SO_2_^•^ that generated CF_3_^•^ by release of SO_2_. Addition of the CF_3_ radical to the alkene led to the radical intermediate **50**, which underwent intramolecular cyclisation into **51**. The sulfate radical anion then oxidised intermediate **51** into the final oxindole (Scheme 26).

**Scheme 27:**
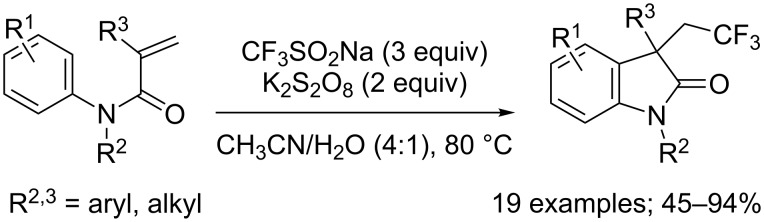
Metal-free carbotrifluoromethylation of *N*-arylacrylamides with CF_3_SO_2_Na/K_2_S_2_O_8_ reported by Wang.

In an independent work Wang and co-workers demonstrated that silver nitrate was not necessary for the reaction to proceed in acetonitrile and water at 80 °C (Scheme 27) [48]. Again, *N*-acyl and *N*–H derivatives failed to deliver the desired products and *meta*-substituted phenyl rings produced mixtures of regioisomers. Under these metal-free conditions, it was proposed that the CF_3_ radical was formed uniquely by reaction of CF_3_SO_2_Na with K_2_S_2_O_8_.

**Scheme 28:**
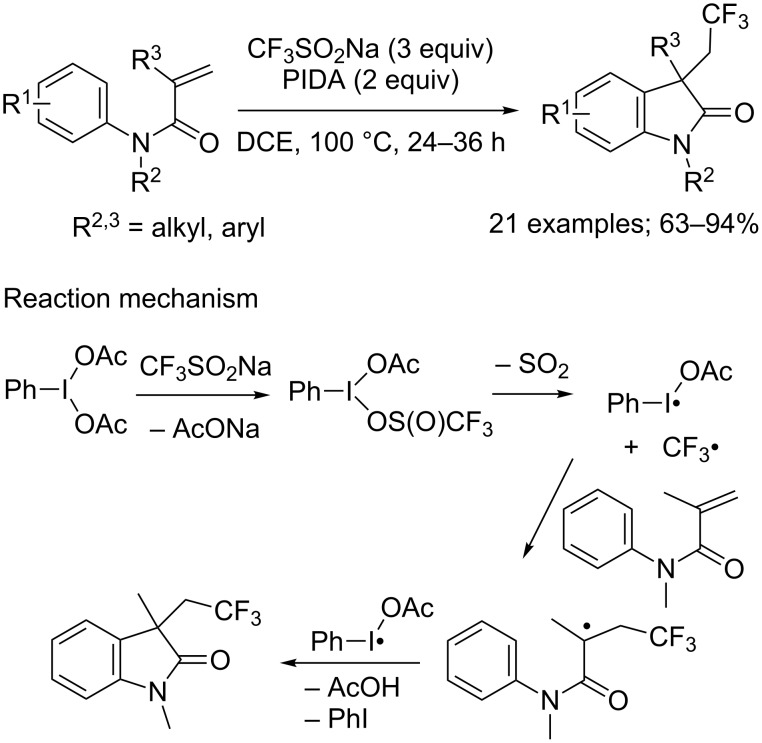
Metal-free carbotrifluoromethylation of *N*-arylacrylamides with CF_3_SO_2_Na/PIDA reported by Fu.

*N*-Arylacrylamides could also react with CF_3_SO_2_Na under metal-free conditions by replacing *tert*-butyl hydroperoxide or the persulfate by hypervalent iodine oxidants such as iodobenzene diacetate (PIDA, Scheme 28) [49], or iodobenzene bis(trifluoroacetate) (PIFA) [50]. Fu and co-workers proposed the reaction mechanism depicted in Scheme 28. PIDA reacted with CF_3_SO_2_Na under heating conditions to produce two radicals: CF_3_^•^ along with PhI^•^OAc. Addition of the CF_3_ radical to the alkene followed by intramolecular cyclisation mediated by PhI^•^OAc gave the desired oxindole with release of PhI and AcOH (Scheme 28) [49].

**Scheme 29:**
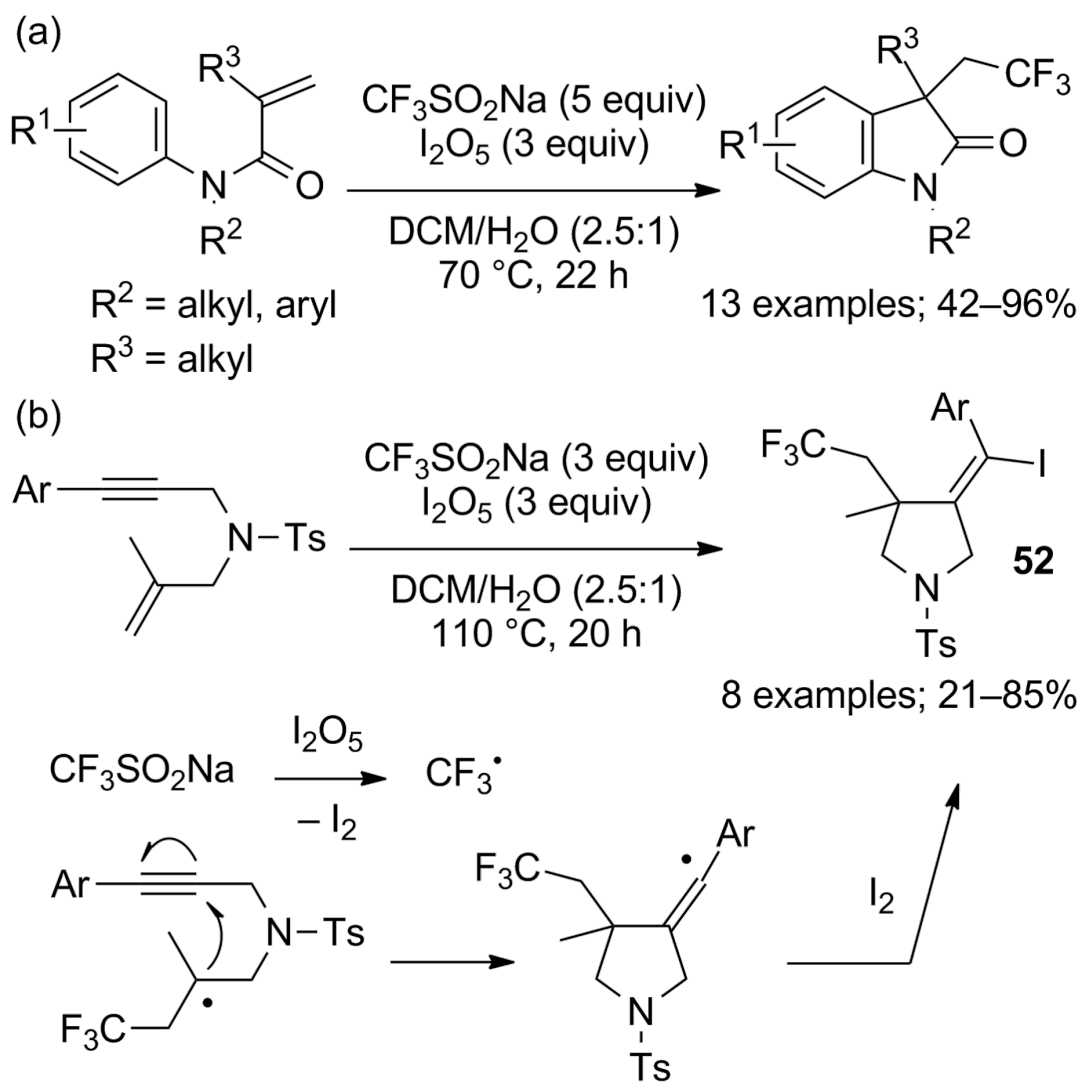
Metal-free cascade trifluoromethylation/cyclisation of *N*-arylmethacrylamides (a) and enynes (b) with CF_3_SO_2_Na/I_2_O_5_.

For another metal-free trifluoromethylation/cyclisation of *N*-arylacrylamides by means of a different oxidant, Liu and co-workers reported the use of CF_3_SO_2_Na in combination with iodide pentoxide in a similar way to their iodotrifluoromethylation of alkenes (see earlier in the text) [42] (Scheme 29a) [51]. Interestingly, this cascade reaction was also applied to enynes for the synthesis of pyrrolidines **52** (Scheme 29b) [51]. A single-electron oxidative free-radical process was ascertained for the generation of CF_3_^•^. From enynes, the iodination step was realised by I_2_, which was formed by a multistep redox process from I_2_O_5_.

**Scheme 30:**
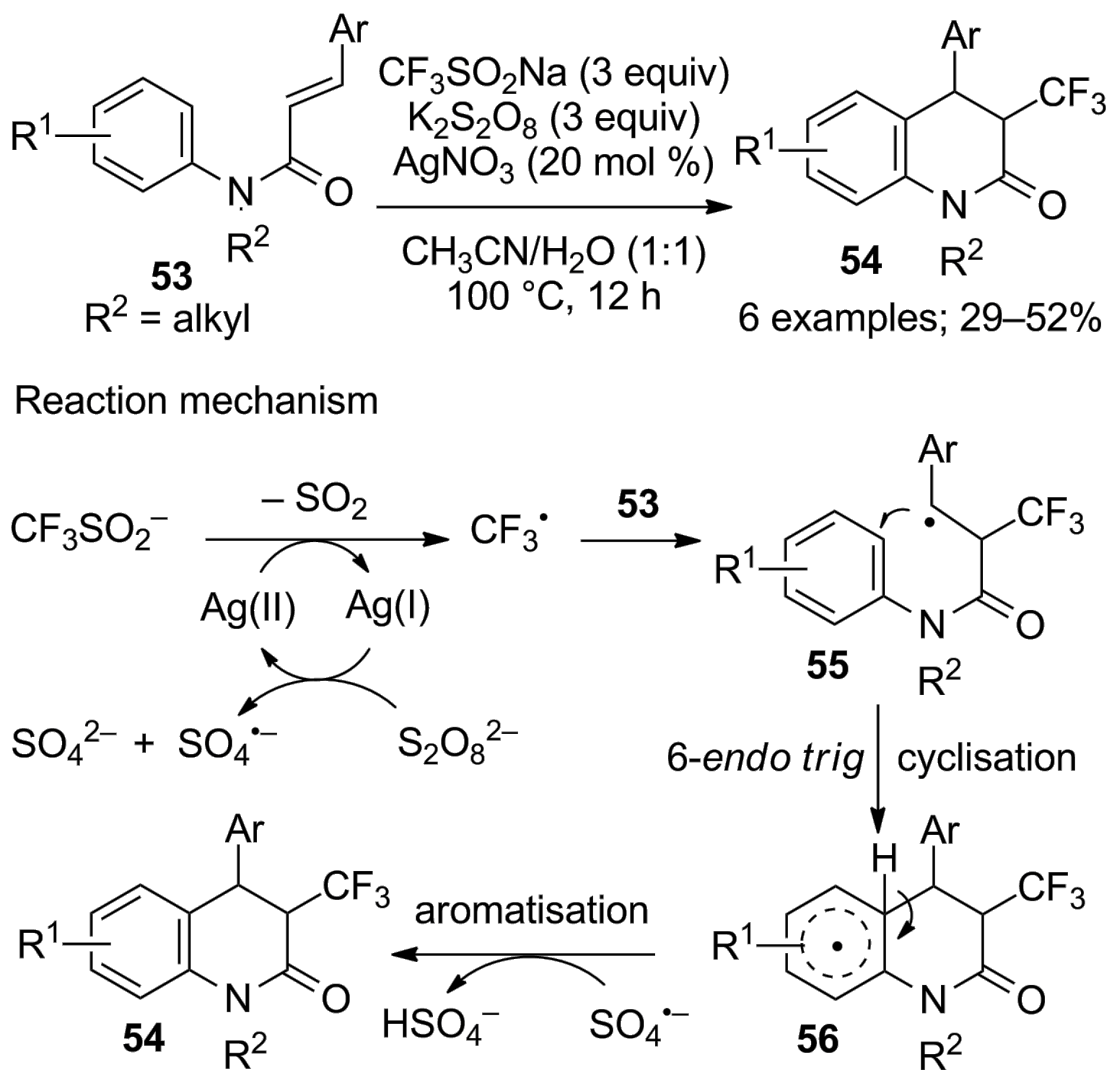
Trifluoromethylation/cyclisation of *N*-arylcinnamamides: Synthesis of 3,4-disubstituted dihydroquinolin-2(1*H*)-ones.

The intramolecular carbotrifluoromethylations of alkenes from acrylamides and methacrylamides, so far described, provided oxindoles via a 5-*exo trig* cyclization. Starting from cinnamamides **53**, Mai, Xiao and co-workers reported a 6-*endo trig* cyclisation leading to 3,4-disubstituted dihydroquinolin-2(1*H*)-ones **54** (Scheme 30) [52]. Ag(I) was oxidised by the persulfate anion (S_2_O_8_^2–^) to generate the Ag(II) cation and the sulfate radical anion; then, the Ag(II) oxidised CF_3_SO_2_Na into CF_3_^•^ with extrusion of SO_2_. The CF_3_ radical reacted with the C=C double bond of the cinnamamide leading to the intermediate **55** that underwent 6-*endo trig* cyclisation to **56** that finally aromatised to the desired product **54 ***trans*-selectively (Scheme 30).

**Scheme 31:**
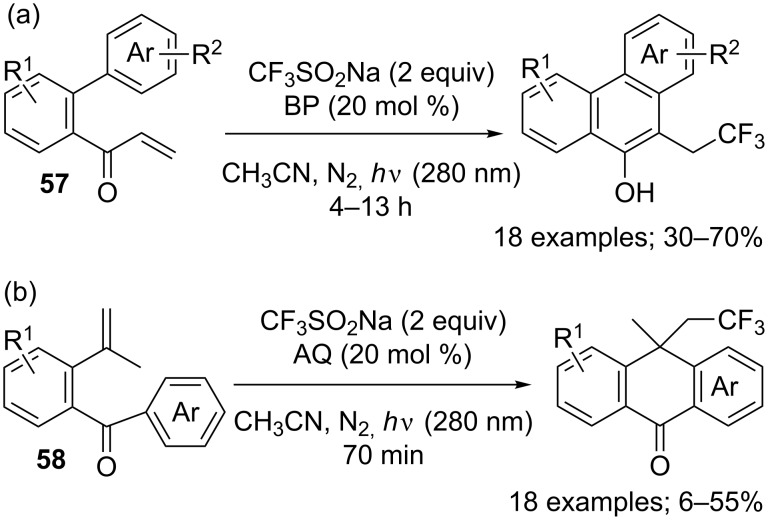
Trifluoromethylation/cyclisation of aromatic-containing unsaturated ketones.

In 2016, Xia and co-workers described a metal-free, UV-light-mediated difunctionalisation of alkenes with CF_3_SO_2_Na for the synthesis of phenanthrene and anthrone derivatives [53]. The substrates were either α,β-unsaturated ketones **57** (Scheme 31a) or γ,δ-unsaturated ketones **58** (Scheme 31b). Benzophenone (BP) or anthracene-9,10-dione (AQ) were used as sensitizers under irradiation using a UV lamp at 280 nm. A radical pathway that involves CF_3_^•^ was established after a negative reaction in the presence of TEMPO (TEMPO–CF_3_ was detected by GC–MS).

**Scheme 32:**
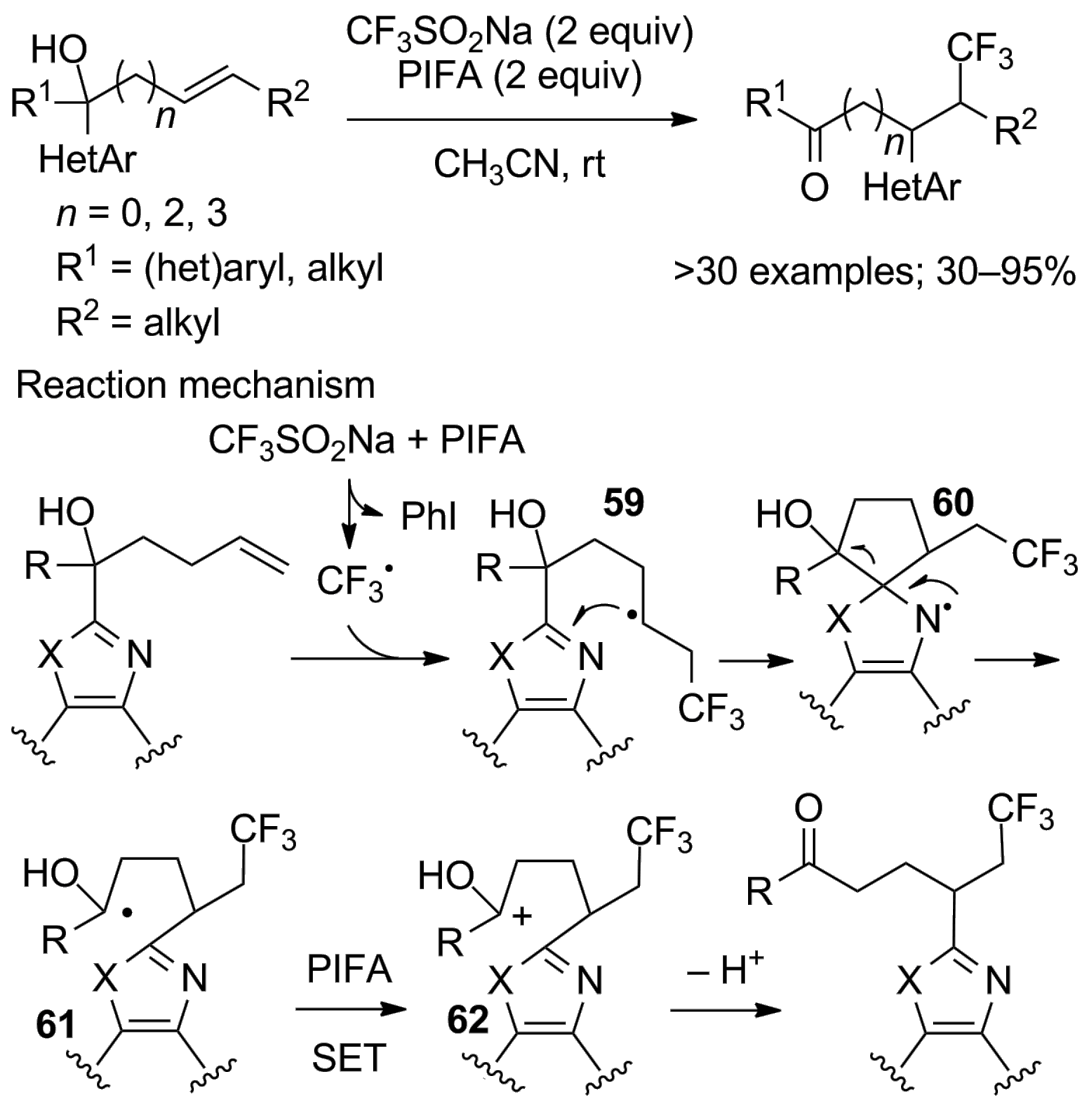
Chemo- and regioselective cascade trifluoromethylation/heteroaryl *ipso*-migration of unactivated alkenes.

An example of difunctionalisation of unactivated alkenes with CF_3_SO_2_Na and an heteroaryl group in which the heteroarylation was realised by a distal heteroaryl *ipso*-migration was provided in 2017 by Zhu and co-workers (Scheme 32) [54]. A variety of nitrogen containing heteroaryl groups showcased the migratory aptitude selectively in the presence of an aryl or an alkyl group. The number of methylene units between the alkene and the tertiary alcohol function was studied: *n* = 0, 2, and 3 were suitable for generating thermodynamically favoured 3, 5, and 6-membered cyclic transition states; the reaction failed with *n* = 1, 4. Experimental and computational studies allowed the authors to propose the mechanism depicted in Scheme 32. First, the CF_3_ radical was generated from CF_3_SO_2_Na and PIFA. Then, addition of CF_3_^•^ to the alkene gave the alkyl radical **59** that added to the *ipso* position of the heteroaryl group to form radical **60**. Next, homolysis of the C–C σ-bond in **60** provided the more stable hydroxyalkyl radical **61**. This radical was oxidised by PIFA to yield the cationic intermediate **62**, which finally lost a proton to furnish the reaction product.

**Scheme 33:**
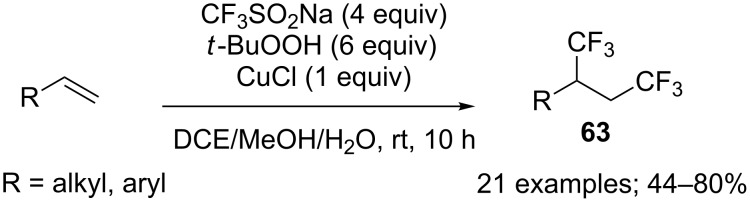
Copper-mediated 1,2-bis(trifluoromethylation) of alkenes.

**1,2-Bis-trifluoromethylation of alkenes:** Alkenes were efficiently and chemoselectively bis-trifluoromethylated under Langlois’ conditions with CF_3_SO_2_Na. Indeed, Qing and co-workers prepared 1,2-bis(trifluoromethylated) compounds **63** with in situ generated CF_3_ radicals (Scheme 33). In order to avoid the formation of dimerised side products, it was demonstrated that an increase of the CF_3_ radical concentration, obtained by increasing the amount of copper catalyst, was beneficial to the chemoselectivity. Both styrene derivatives and terminal unactivated alkenes were suitable substrates in this transformation but not internal alkenes [55].

#### C_sp2_–CF_3_ bond-forming reactions

**Scheme 34:**
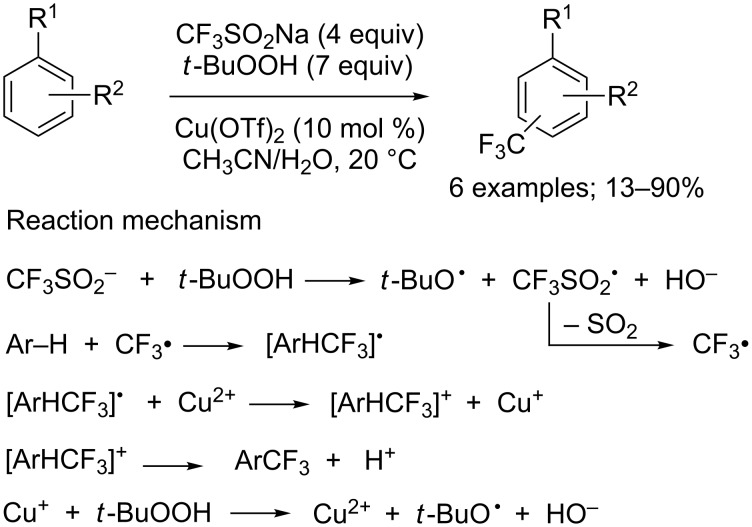
Trifluoromethylation of aromatics with CF_3_SO_2_Na reported by Langlois.

**Direct trifluoromethylation of arenes and heteroarenes:** In 1991, Langlois and co-workers reported the first trifluoromethylation of aromatic compounds with sodium trifluoromethanesulfinate under oxidative conditions (Scheme 34) [16]. The scope was quite narrow with electron-rich aromatics and mixtures of regioisomers were often obtained. For instance, from aniline, two isomers were obtained in 13% overall yield, and from 1,3-dimethoxybenzene, four products (regioisomers + bis-CF_3_ compounds) were obtained in 90% overall yield. For this transformation, a radical process was proposed: the trifluoromethyl radical CF_3_**^•^** was generated by reaction of *tert*-butyl hydroperoxide with CF_3_SO_2_Na in the presence of a copper(II) catalyst (Scheme 34).

Substrates with sensitive functional groups may not be tolerated under such reaction conditions and a large excess amount of peroxide was necessary to reach high yields. That is how, in 1998, Smertenko and co-workers described a milder electrochemical trifluoromethylation of a series of aromatic compounds using CF_3_SO_2_Na in acetonitrile [56]. Furthermore, the electrochemical oxidation of the trifluoromethylsulfinate anion (from CF_3_SO_2_K) generated the trifluoromethyl radical for the reaction of electron-rich aromatics and alkenes [38].

**Scheme 35:**
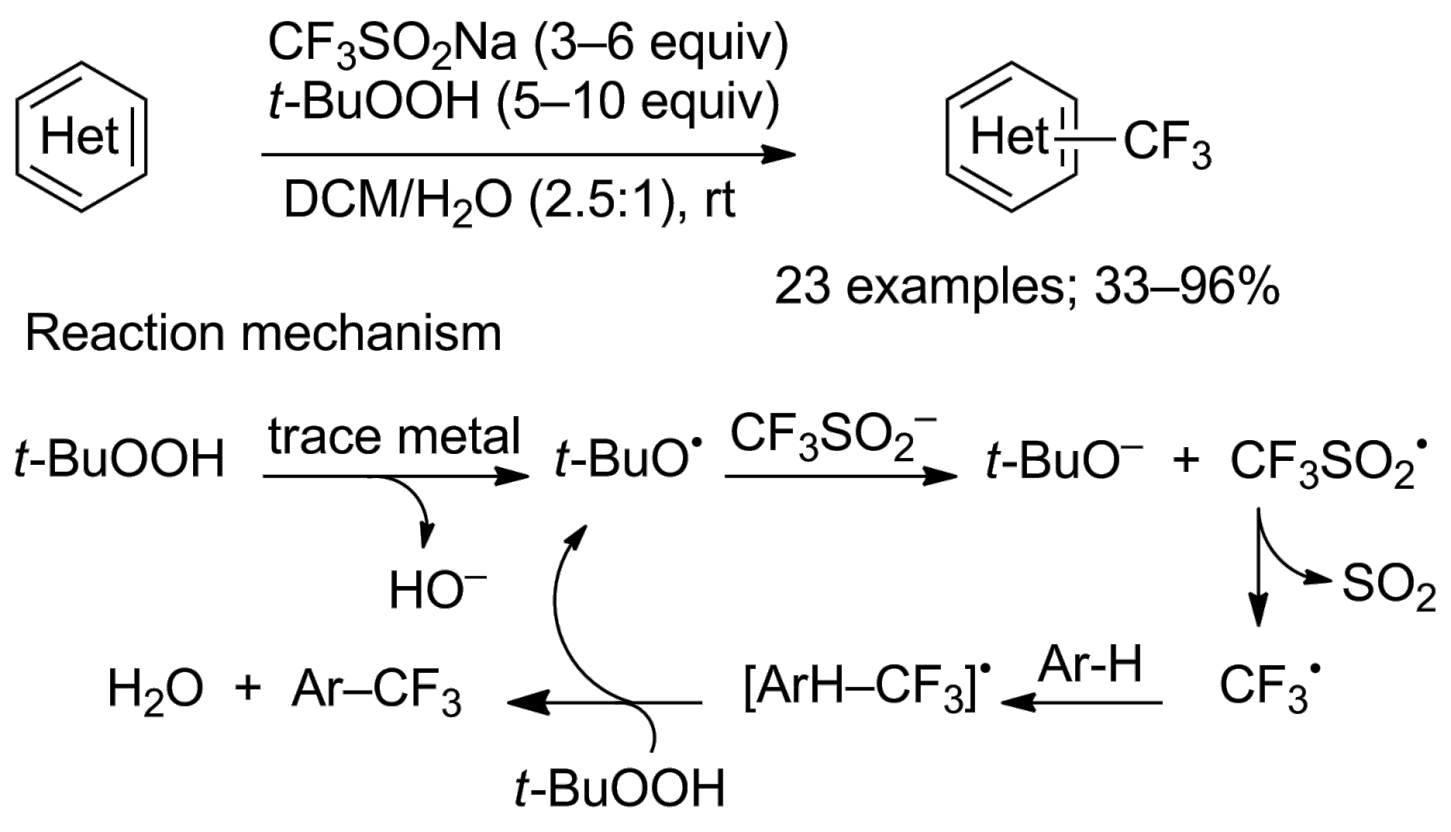
Baran’s oxidative C–H trifluoromethylation of heterocycles.

It is twenty years after Langlois’ pioneering work that the direct trifluoromethylation of heteroaromatic compounds was re-investigated by Baran and co-workers in 2011 [57]. This group reported a C−H trifluoromethylation protocol that was operationally simple, scalable, achieved at room temperature, working with a variety of electron-deficient and -rich heteroaromatic systems tolerating various functional groups such as unprotected alcohols, amines, ketones, esters, halides and nitriles. Importantly, the trifluoromethylation proceeded at the innate reactive positions of the heterocycles; however, it was noticed that the regioselectivity can be tuned simply by solvent choice. Langlois and others employed catalytic metal salts for reaction initiation but Baran’s group demonstrated that metal additives were not required for a productive reaction, only trace metals found in Langlois’ reagent could be responsible for reaction initiation. The scope was evaluated on pyridines, pyrroles, indoles, pyrimidines, pyrazines, phthalazines, quinoxalines, deazapurine, thiadiazoles, uracils, xanthenes and pyrazolino-pyrimidines (Scheme 35). The combination of previous studies with new observations allowed to propose a putative mechanism (Scheme 35) as well as unproductive pathways (formation of CF_3_H from CF_3_^•^ by abstraction of a hydrogen atom or reaction of CF_3_^•^ with isobutene generated from *t*-BuOOH).

This oxidative trifluoromethylation method was exploited for the synthesis of modified nucleosides, in particular 8-CF_3_-2’-deoxyguanosine and 8-CF_3_-inosine in 39 and 73% yields, respectively [58].

**Scheme 36:**
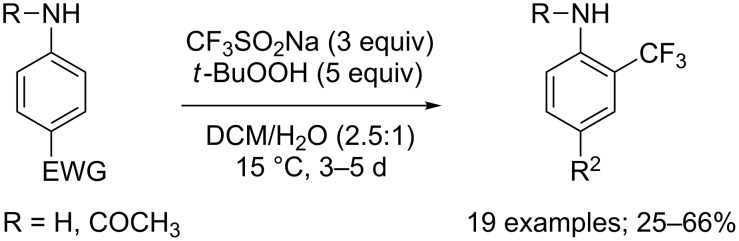
Trifluoromethylation of acetanilides and anilines.

The same copper-free method was applied for the trifluoromethylation of a variety of electron-deficient 4-substituted acetanilides or anilines (Scheme 36). In these reaction conditions, Cao’s group reported that acetanilides or anilines featuring electron-donating substituents at the *para*-position of the acetamino group afforded mixtures of isomeric C−H trifluoromethylation products in moderate yields. However, with substrates bearing electron-withdrawing groups, *ortho*-CF_3_ acetanilides or anilines were obtained as sole products [59].

**Scheme 37:**
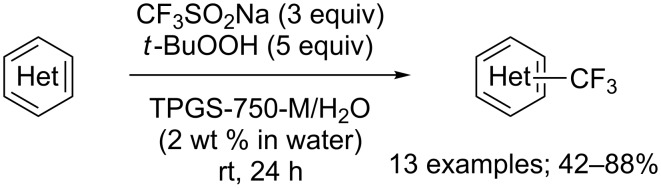
Trifluoromethylation of heterocycles in water.

To meet high expectations of environmentally low impact chemical reactions, Lipshutz and co-workers carried out the trifluoromethylation of heterocycles using aqueous micellar conditions based on the surfactant TPGS−750−M in water at room temperature. The trifluoromethyl radical was generated from CF_3_SO_2_Na and *t*-BuOOH. In comparaison to Baran’s results, in all cases, the yields were improved. Advantageously, the aqueous medium can be recycled (Scheme 37) [60].

**Scheme 38:**
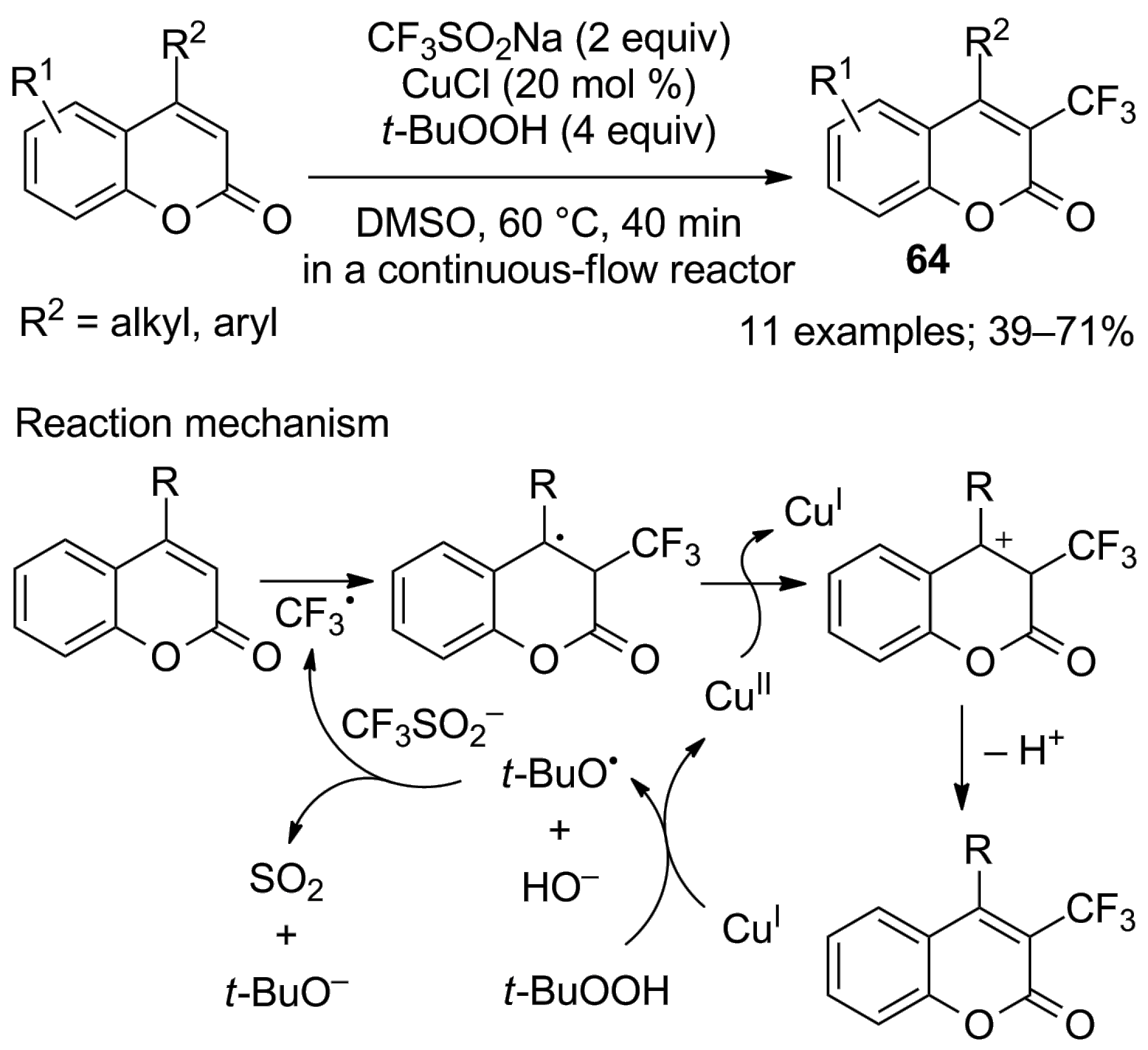
Trifluoromethylation of coumarins in a continuous-flow reactor.

Even though it is a proven fact after 2011 and Baran’s work that no-added metal trifluoromethylation with CF_3_SO_2_Na are highly efficient, the original Langlois’ conditions were nevertheless applied to a series of heteroarenes. Li and co-workers reported the synthesis of 3-trifluoromethylcoumarins **64** by Cu(I)-catalysed trifluoromethylation with CF_3_SO_2_Na and *t*-BuOOH in a continuous-flow reactor [61]. After optimisation of the reaction conditions in batch, the optimal reaction conditions were established in a continuous-flow reactor at a flow rate of 100 µL min^−1^ at 60 °C for 40 min. The substrate scope was evaluated on 11 coumarins and showed that both electron-rich and electron-deficient functional groups were tolerated (Scheme 38).

**Scheme 39:**
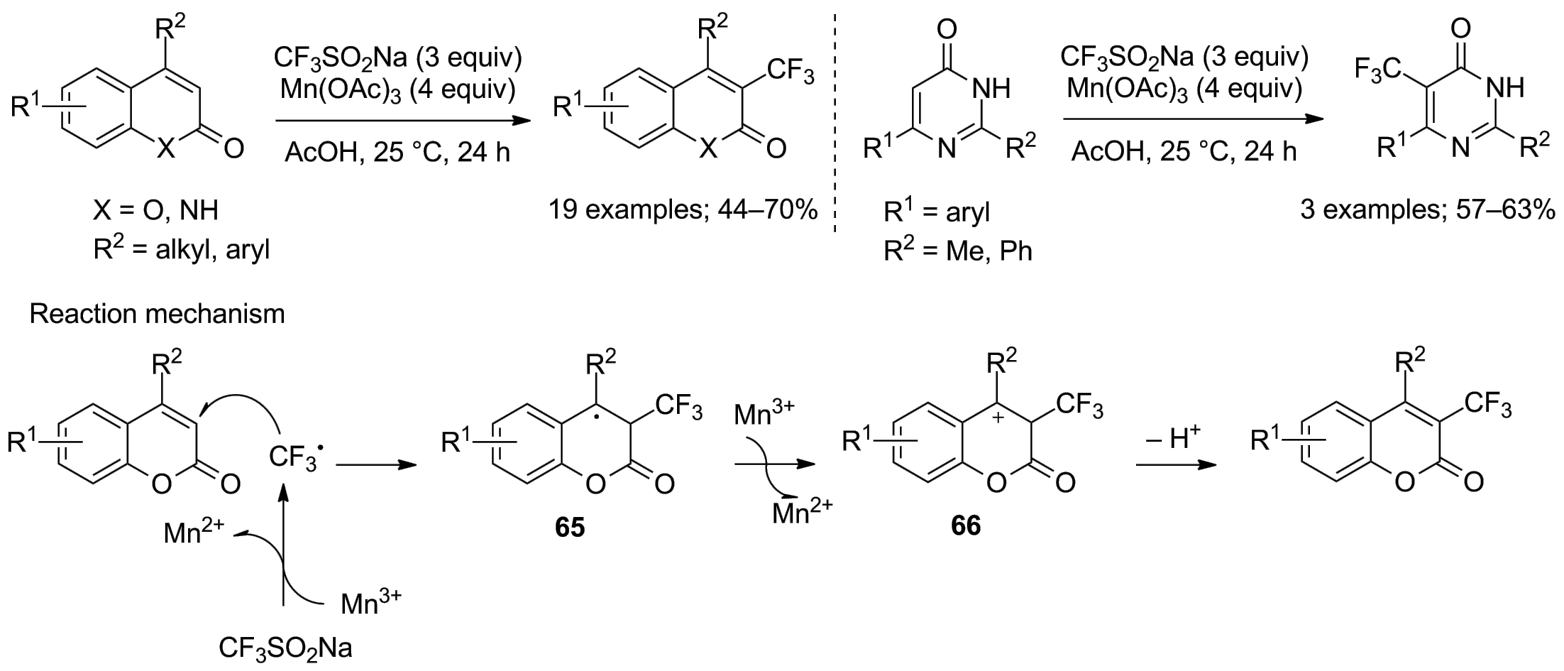
Oxidative trifluoromethylation of coumarins, quinolines and pyrimidinones.

Zou and co-workers applied the conditions described by Langlois or Baran (CF_3_SO_2_Na/*t*-BuOOH/cat. Cu(II) or CF_3_SO_2_Na/*t*-BuOOH, respectively) for the trifluoromethylation of coumarins but no reaction was observed. By testing other oxidants, they found that Mn(OAc)_3_ was a good oxidant for this reaction and allowed to carry out the trifluoromethylation exclusively at the α-position of the carbonyl group in the pyranone ring. The substrate scope was large and included 17 coumarins, 2 quinolines and 3 pyrimidinones. With coumarins bearing electron-donating groups on the phenyl ring, the 3-trifluoromethylated compounds were obtained in 50–56% yields. However, coumarins bearing electron-withdrawing groups gave yields up to 70%. As for the mechanism, the trifluoromethyl radical was generated from CF_3_SO_2_Na and Mn(OAc)_3_, then CF_3_^•^ added regioselectively onto the coumarin to give intermediate radical **65**, which was oxidised by Mn(OAc)_3_ to form the carbocation **66** and, after deprotonation, the trifluoromethyl compounds (Scheme 39) [62].

**Scheme 40:**
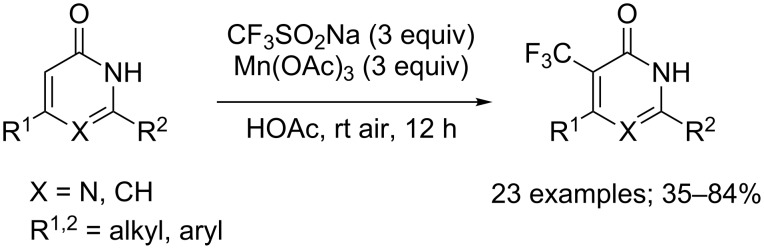
Oxidative trifluoromethylation of pyrimidinones and pyridinones.

The same group also reported a straightforward method for the trifluoromethylation of pyrimidinones and pyridinones under the same reaction conditions. 5-Trifluoromethylpyrimidinones and 3-trifluoromethylpyridinones were selectively obtained in moderate to good yields (Scheme 40). It was observed that the substituent R^1^ provided no stabilisation for the radical intermediate, so the less bulky substituents at 6-position of pyrimidinones or 4-position of pyridinones facilitated the trifluoromethyl radical attack [63].

**Scheme 41:**
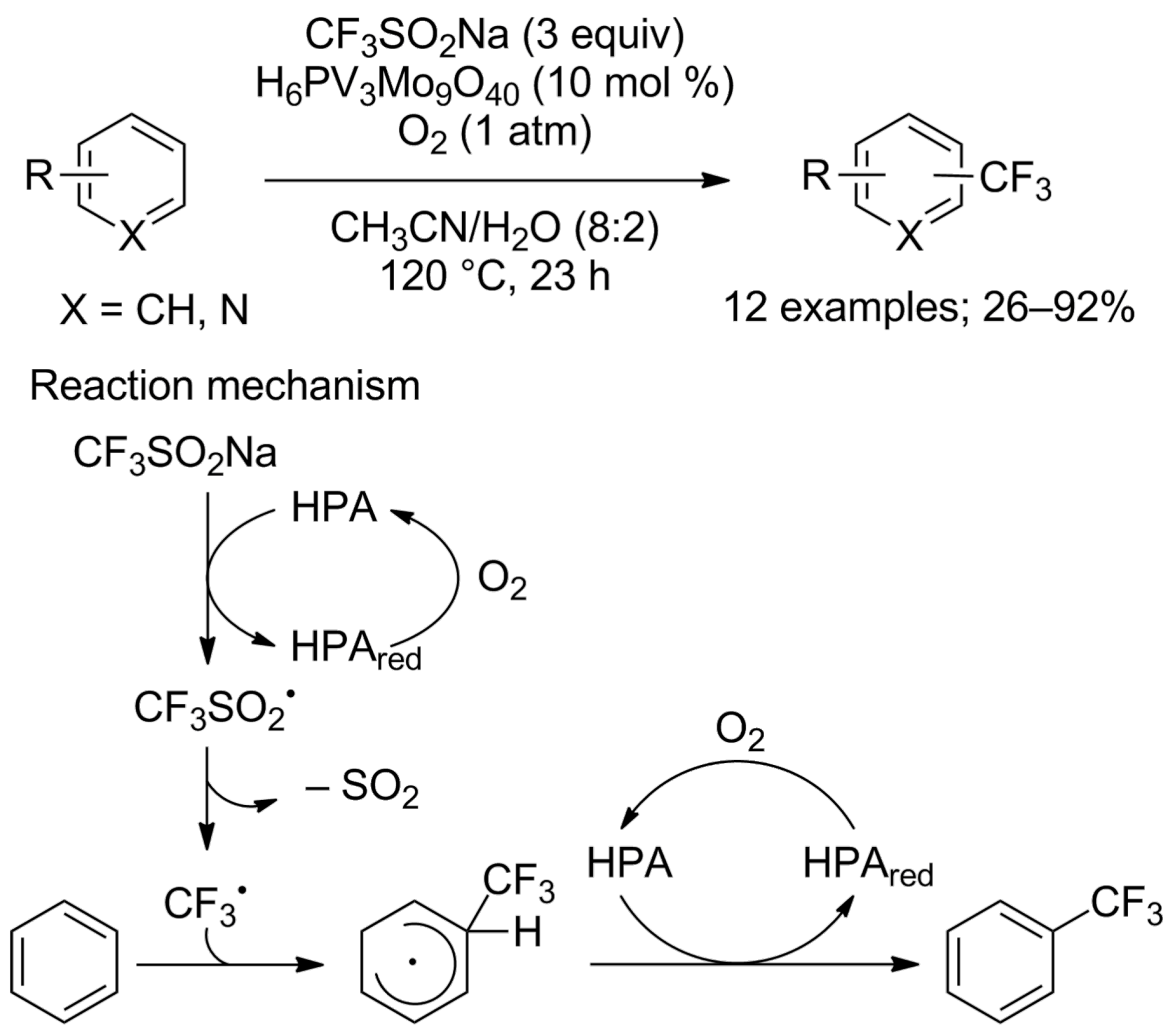
Phosphovanadomolybdic acid-catalysed direct C−H trifluoromethylation.

Catalytic amounts of phosphovanadomolybdic acid, a heteropolyacid catalyst (HPA), was used by Mizuno, Yamaguchi and co-workers for the oxidative C−H trifluoromethylation of arenes and heteroarenes in the presence of CF_3_SO_2_Na and O_2_ as the terminal oxidant. This method allowed the trifluoromethylation of arenes bearing electron-donating as well as electron-withdrawing groups in moderate to good yields (Scheme 41) [64]. It has to be noted that bis-CF_3_ products as well as regioisomers were also characterised or detected in small amounts in most cases. A radical mechanism was proposed as described in Scheme 41.

**Scheme 42:**
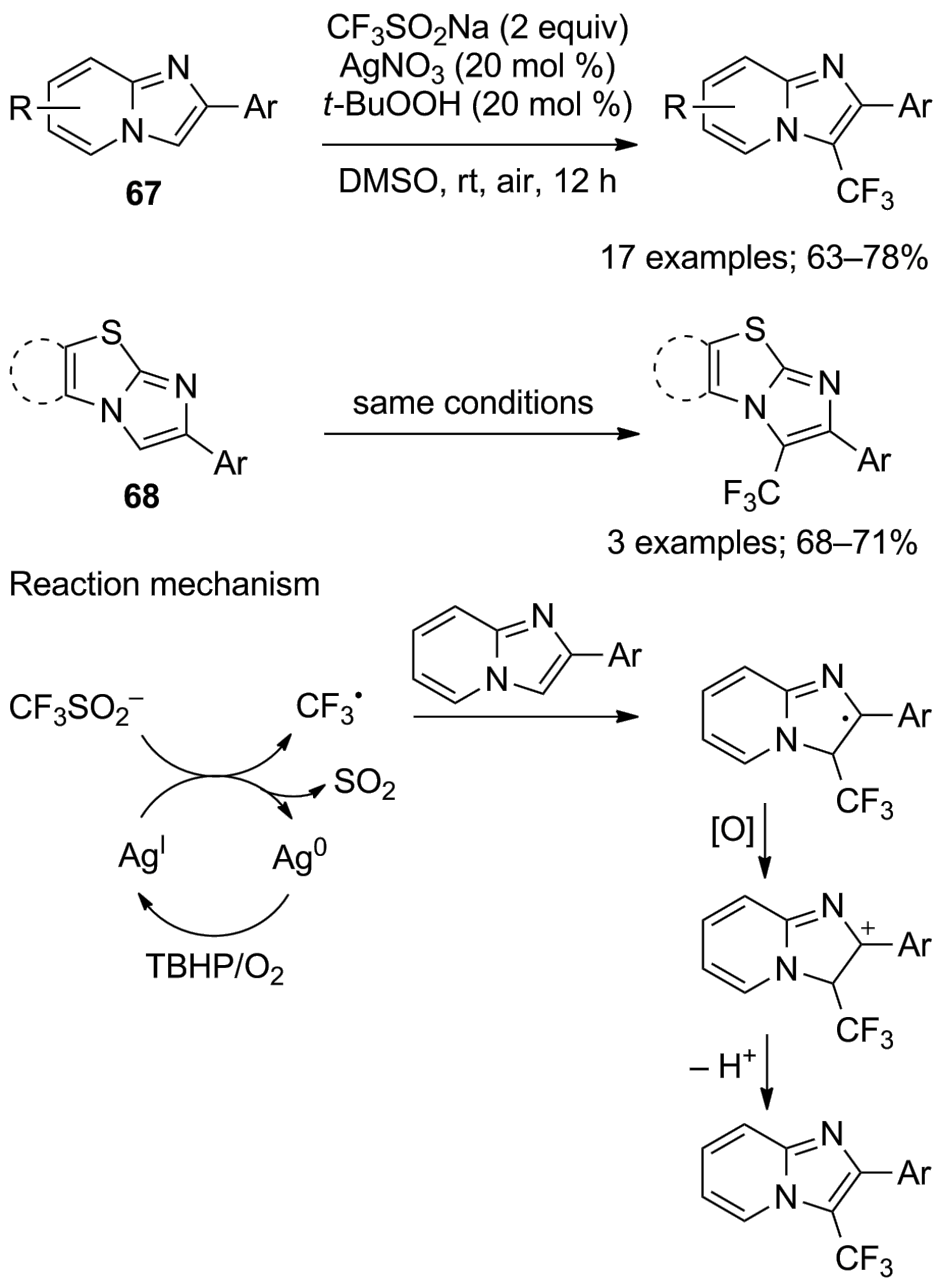
Oxidative trifluoromethylation of imidazopyridines and imidazoheterocycles.

Imidazopyridines have demonstrated many interesting features toward biological activities and the incorporation of a trifluoromethyl group into such architectures was expected to alter their properties. Therefore, Hajra and co-workers reported a direct and regioselective method for the trifluoromethylation of imidazopyridines **67** and other imidazoheterocycles **68** [65]. The combination CF_3_SO_2_Na/*t*-BuOOH/cat. AgNO_3_ at room temperature under air was applied to 17 imidazopyridines and 3 imidazoheterocycles (Scheme 42). Good yields were obtained when the phenyl moiety was substituted by electron-donating groups. As a result of absence of reactivity in presence of the radical scavenger TEMPO, a radical pathway was proposed. Under argon atmosphere, only trace amounts of the CF_3_ product were obtained clearly indicating the crucial role of aerial oxygen in the catalytic cycle (see mechanism in Scheme 42).

**Scheme 43:**
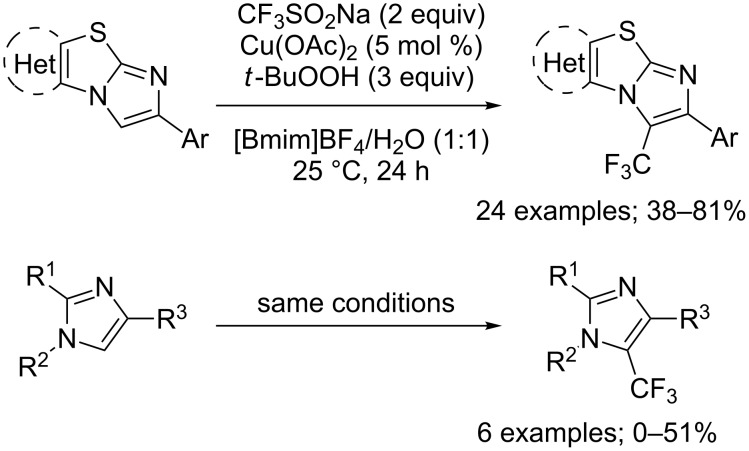
Oxidative trifluoromethylation of imidazoheterocycles and imidazoles in ionic liquid/water.

Simultaneously, Tang and co-workers reported a greener strategy for the trifluoromethylation of imidazoheterocycles with CF_3_SO_2_Na in a recyclable mixed medium of 1-butyl-3-methylimidazolium tetrafluoroborate ([Bmim]BF_4_) and water [66]. The substrate scope was investigated on 11 imidazothiazoles, 13 imidazo[1,2-*a*]pyridines and 6 imidazoles (Scheme 43). The reaction was simple, achieved at room temperature and had a good tolerance for various functional groups. However, for the trifluoromethylation of imidazoles, it was essential to have a phenyl as a substituent in order to get good yields. A radical mechanism was proposed but the role of oxygen was not discussed.

**Scheme 44:**
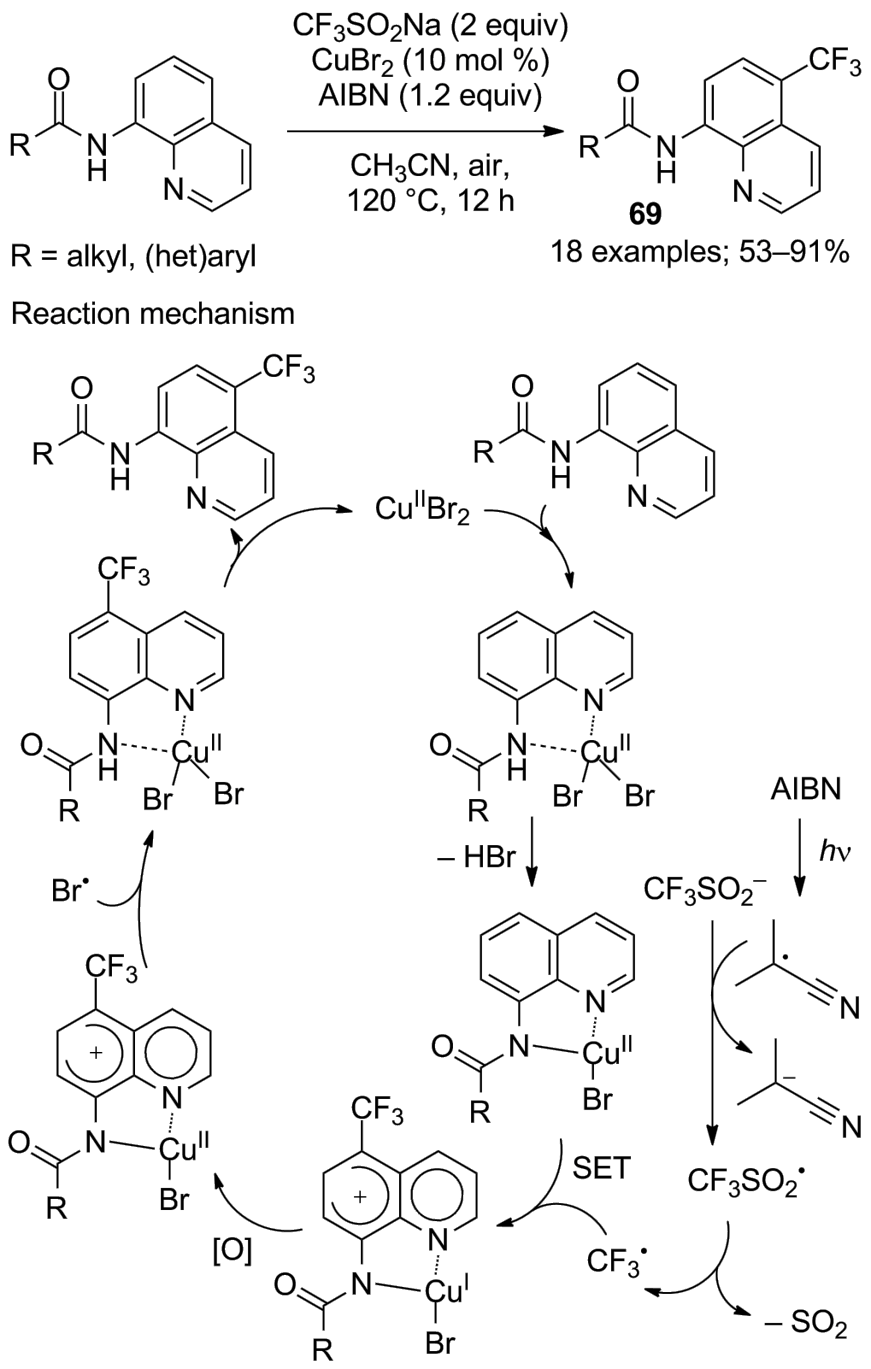
Oxidative trifluoromethylation of 8-aminoquinolines.

Aminoquinoline derivatives are found in naturally occurring and synthetic bioactive compounds, most notable for their antimalarial activity. So, it was not surprising that trifluoromethyl analogues were prepared in particular by means of CF_3_SO_2_Na. Indeed, 5-trifluoromethyl-8-aminoquinoline derivatives **69** were regioselectively synthesised under various reaction conditions. Cai and co-workers reported the trifluoromethylation of 8-aminoquinolines selectively at position 5 by using CF_3_SO_2_Na, CuBr_2_ in a catalytic amount and azobisisobutyronitrile (AIBN) as an oxidant (Scheme 44) [67]. This process had a broad tolerance toward a wide range of functional groups. Aliphatic amides, aromatic amides and carboxamides with heterocyclic substituents were compatible with the reaction conditions. A series of control experiments that included the inhibition of the reaction in the presence of TEMPO, deuteration and isotope effect experiments were carried out and led the authors to propose the single-electron transfer mechanism presented in Scheme 44.

**Scheme 45:**
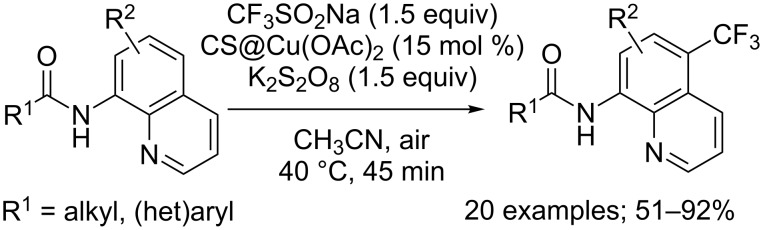
Oxidative trifluoromethylation of various 8-aminoquinolines using the supported catalyst CS@Cu(OAc)_2_.

Simultaneously, Shen, Zhang and co-workers reported a milder regioselective trifluoromethylation of 8-aminoquinolines using the supported catalyst CS@Cu(OAc)_2_ (CS = chitosan), potassium persulfate as the oxidant and CF_3_SO_2_Na as the CF_3_^•^ source (Scheme 45) [68]. After optimisation of the reaction conditions, the authors studied the effect of structural variations in the substrate (R^1^ = aryl, heteroaryl, alkyl; R^2^ = H, 6-OMe, 2-Me). Aniline amides gave no conversion nor did quinoline having an ester group at the 8 position instead of the 8-amino group. The chitosan-based copper catalyst was efficiently reused in five cycles of this heterogeneous trifluoromethylation.

**Scheme 46:**
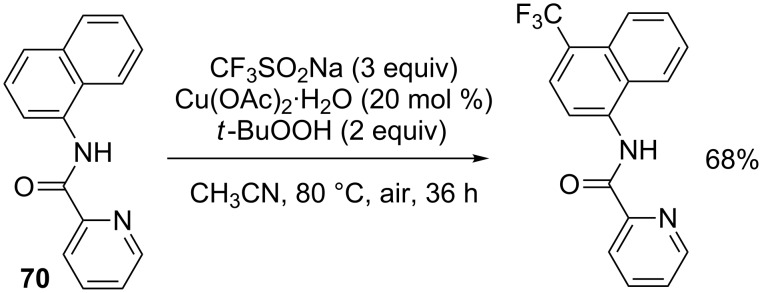
Oxidative trifluoromethylation of the naphthylamide **70**.

In 2017, Lu, Weng and co-workers reported a protocol for the *para*-selective trifluoromethylation of naphthylamide **70**, instead of the previously studied quinolines, with CF_3_SO_2_Na, *tert*-butyl hydroperoxide and Cu(OAc)_2_·H_2_O as oxidant (Scheme 46) [69].

**Scheme 47:**
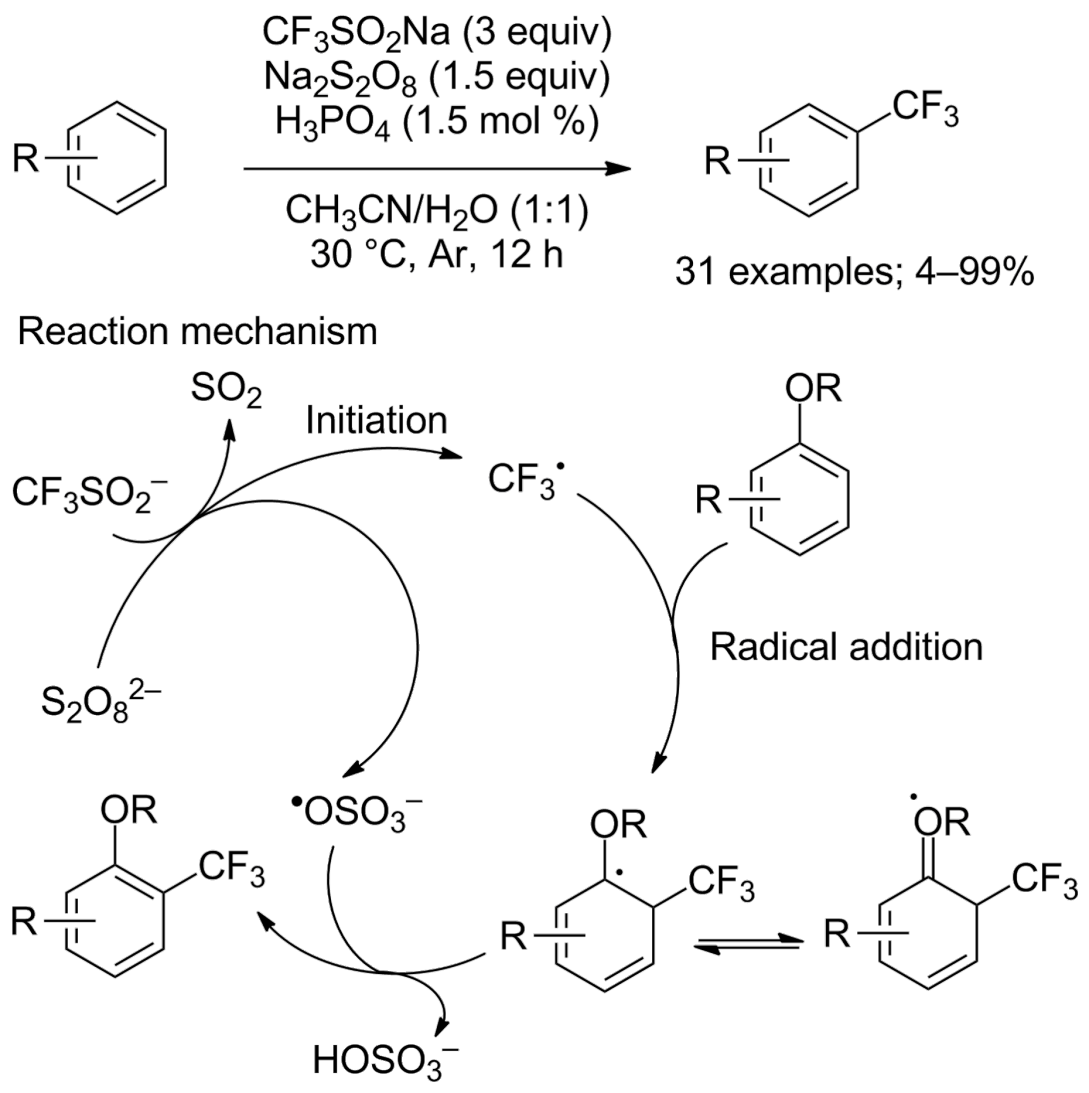
Oxidative trifluoromethylation of various arenes in the presence of CF_3_SO_2_Na and sodium persulfate.

*tert*-Butyl hydroperoxide could be replaced by sodium persulfate as demonstrated in 2016 by Gong and co-workers who reported a direct C−H trifluoromethylation of arenes with CF_3_SO_2_Na in a mixture of water and acetonitrile (Scheme 47) [70]. Various trifluoromethylated arenes were obtained in moderate to excellent yields. However, to achieve high yields in this trifluoromethylation, one (or more) alkoxy group(s) must be present on the arenes in order to stabilise the free-radical intermediate (see mechanism in Scheme 47). Control experiments such as the trapping of the trifluoromethyl radical with the scavenger TEMPO or with benzoquinone were performed and a radical process was proposed. This mild and safe transformation had a good tolerance for various functional groups.

**Scheme 48:**
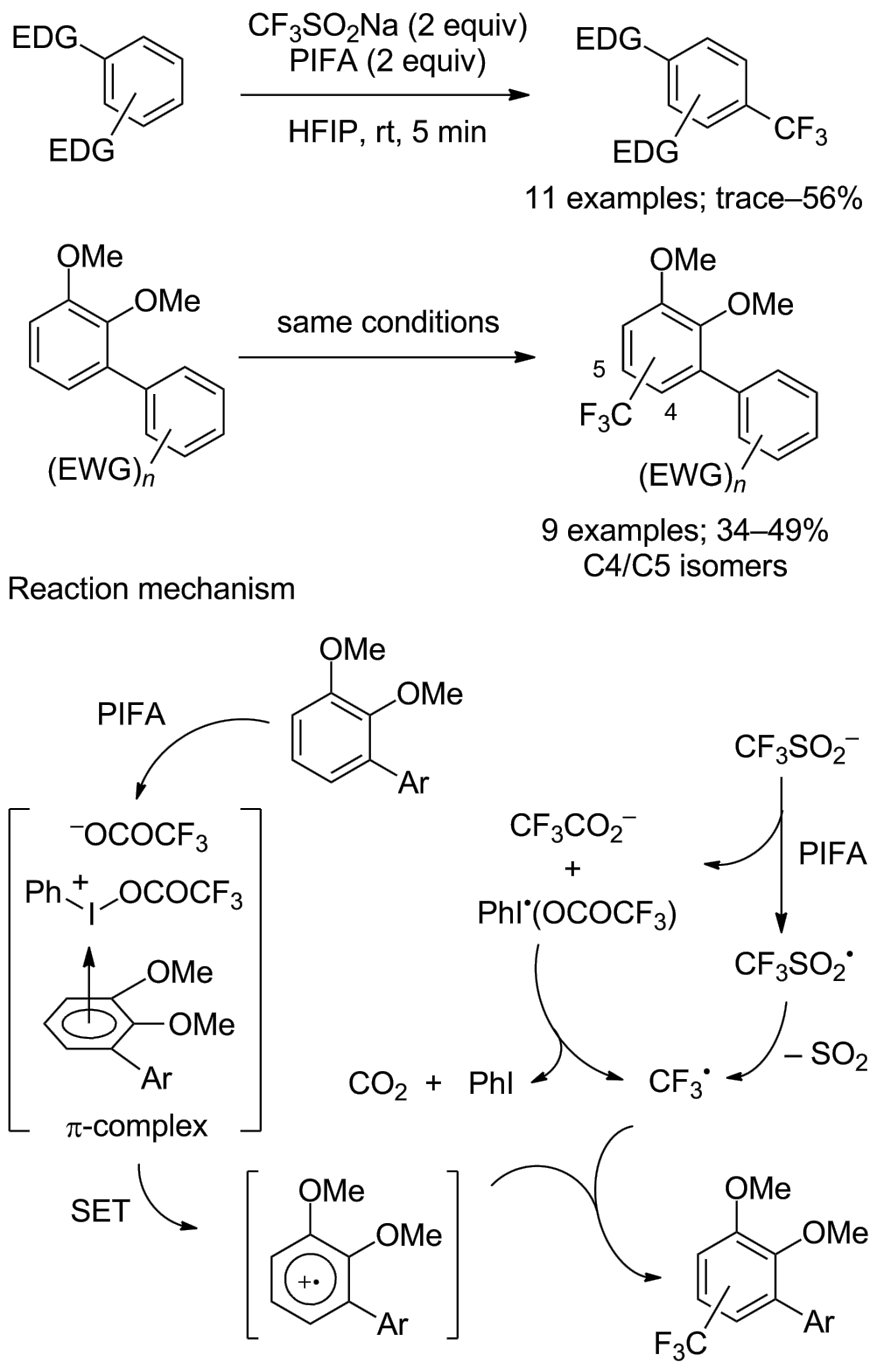
Trifluoromethylation of electron-rich arenes and unsymmetrical biaryls with CF_3_SO_2_Na in the presence of PIFA.

In 2013, Shibata and co-workers reported a transition-metal-free oxidative trifluoromethylation of arenes with CF_3_SO_2_Na and phenyliodine bis(trifluoroacetate) (PIFA) instead of *tert*-butyl hydroperoxide as the oxidant in hexafluoroisopropanol (HFIP) at room temperature [71]. In order to obtain good results for this transformation, it should be noted that the electron-donating nature of the aromatic substituents was a crucial point. In the case of unsymmetrical biaryl substrates, mixtures of regioisomers at C4 and C5 were obtained (Scheme 48). In the reaction mechanism, PIFA played a dual role in the activation of the arene via a π-complex and in the generation of the CF_3_ radical from CF_3_SO_2_Na.

**Figure 1:**
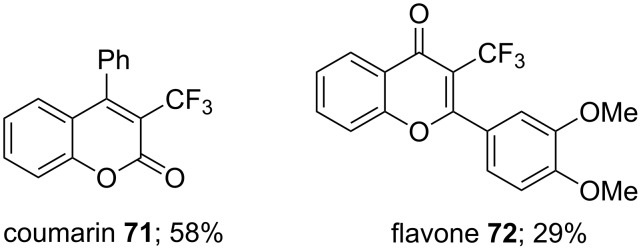
Trifluoromethylated coumarin and flavone.

More recently, Maruoka and co-workers reported the synthesis of trifluoromethylated coumarin **71** and flavone **72** with CF_3_SO_2_Na (2 equiv), the hypervalent iodine F_5_-PIFA (pentafluorophenyliodine bis(trifluoroacetate)) (2 equiv) and 2,3-dichloro-5,6-dicyano-1,4-benzoquinone (DDQ, 0.6 equiv). The trifluoromethylated compounds were obtained in moderate yields (Figure 1) [50].

**Scheme 49:**
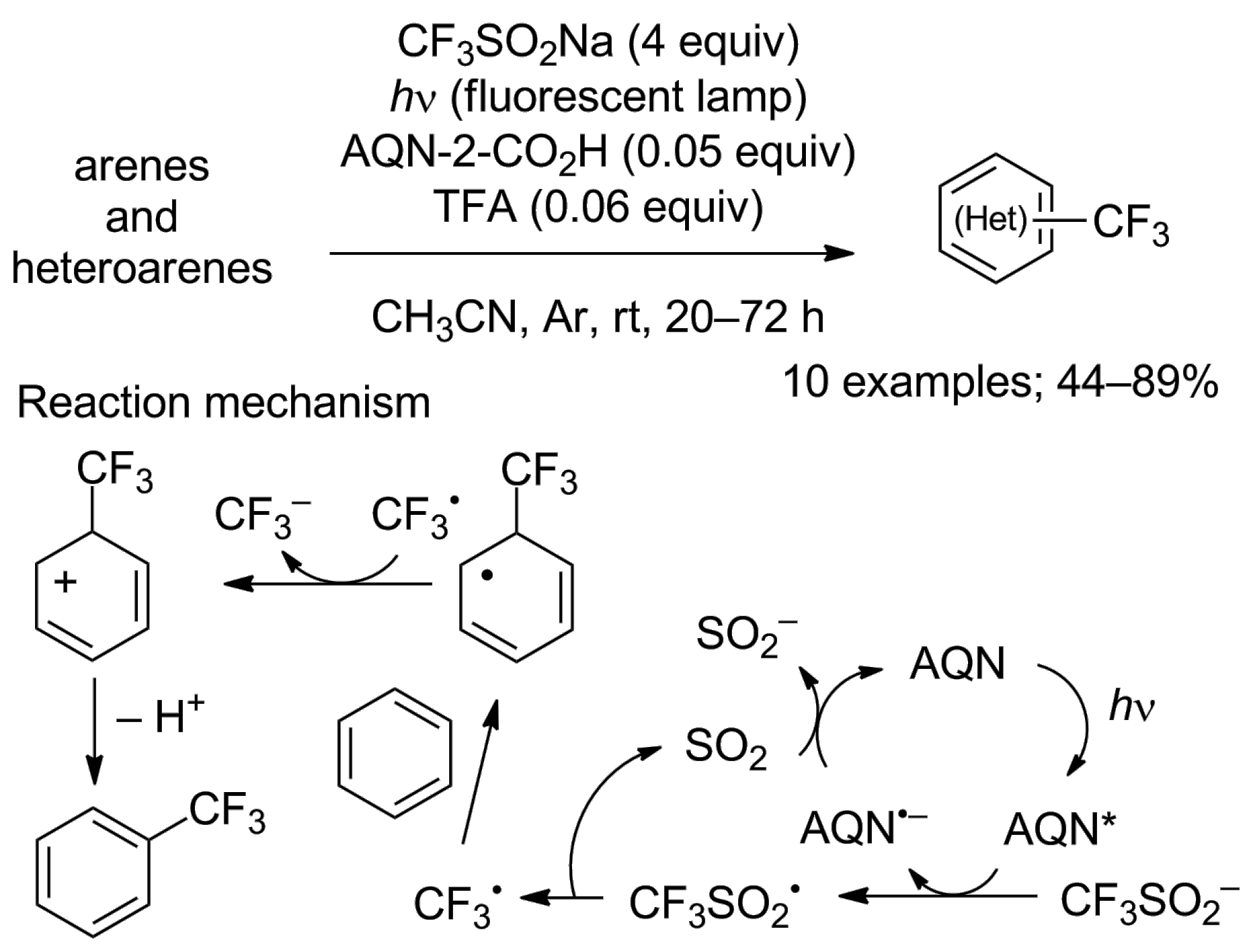
Metal-free trifluoromethylation catalysed by a photoredox organocatalyst.

To avoid the use of transition-metal catalysts and/or an excess amount of oxidants, the Itoh group reported a simple metal-free, direct trifluoromethylation of arenes and heteroarenes using a photoredox-based process under visible-light irradiation. This method used CF_3_SO_2_Na as source of the trifluoromethyl group and a catalytic amount of anthraquinone-2-carboxylic acid (AQN-2-CO_2_H). The scope was achieved on arenes and heteroarenes (10 examples). Once more, electron-rich aromatic compounds were converted into the corresponding trifluoromethylated products in good yields (Scheme 49). The oxidation–reduction potentials were determined by cyclic voltammetry and a catalytic cycle was proposed in which the CF_3_ radical was generated from CF_3_SO_2_Na via the organocatalyst AQN-2-CO_2_H and visible light (Scheme 49) [72].

**Scheme 50:**
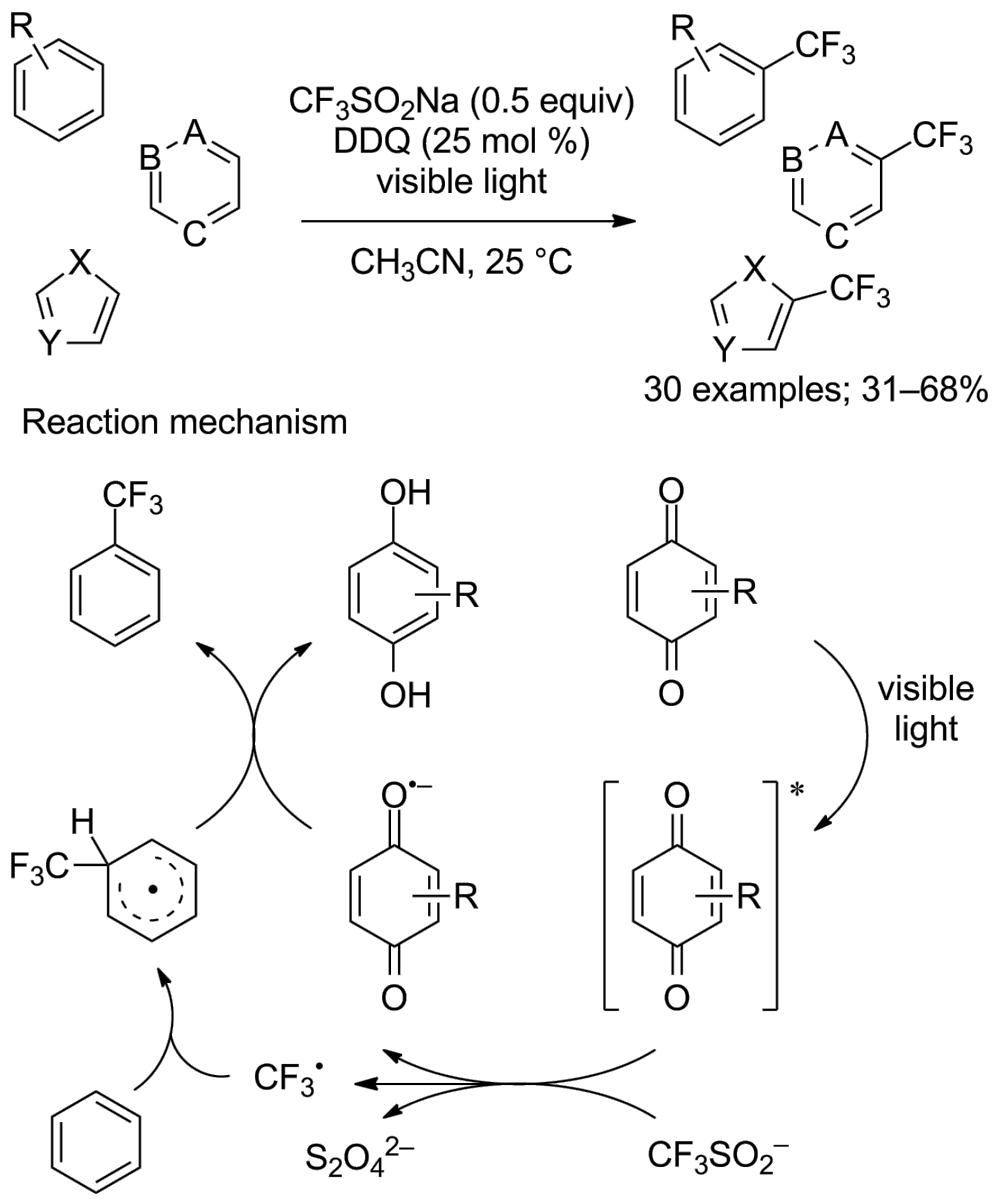
Quinone-mediated trifluoromethylation of arenes and heteroarenes.

Rueping’s group described three examples of prototypical (hetero)aromatic substrates that were trifluoromethylated with CF_3_SO_2_Na in the presence of 4,4’-dimethoxybenzophenone as photosensitiser under near-UV irradiation (350 nm) and HFIP as a proton donor [40]. More recently, Yuan and co-workers reported an efficient and operationally simple method for the direct trifluoromethylation of a wide variety of arenes and heteroarenes under visible-light irradiation [73]. The substrate scope was evaluated on 30 arenes and heteroarenes using 2,3-dichloro-5,6-dicyanobenzoquinone (DDQ) as photocatalyst and CF_3_SO_2_Na as the CF_3_ radical source. The reaction conditions tolerated a broad range of functional groups and the yields ranged from 31 to 68% (Scheme 50). During the process, the quinone was converted into hydroquinone and a regeneration process was established by passing through a cartridge of MnO_2_. A mechanism for this transformation was proposed as a result of electron paramagnetic resonance spectroscopy experiments (Scheme 50).

**Scheme 51:**
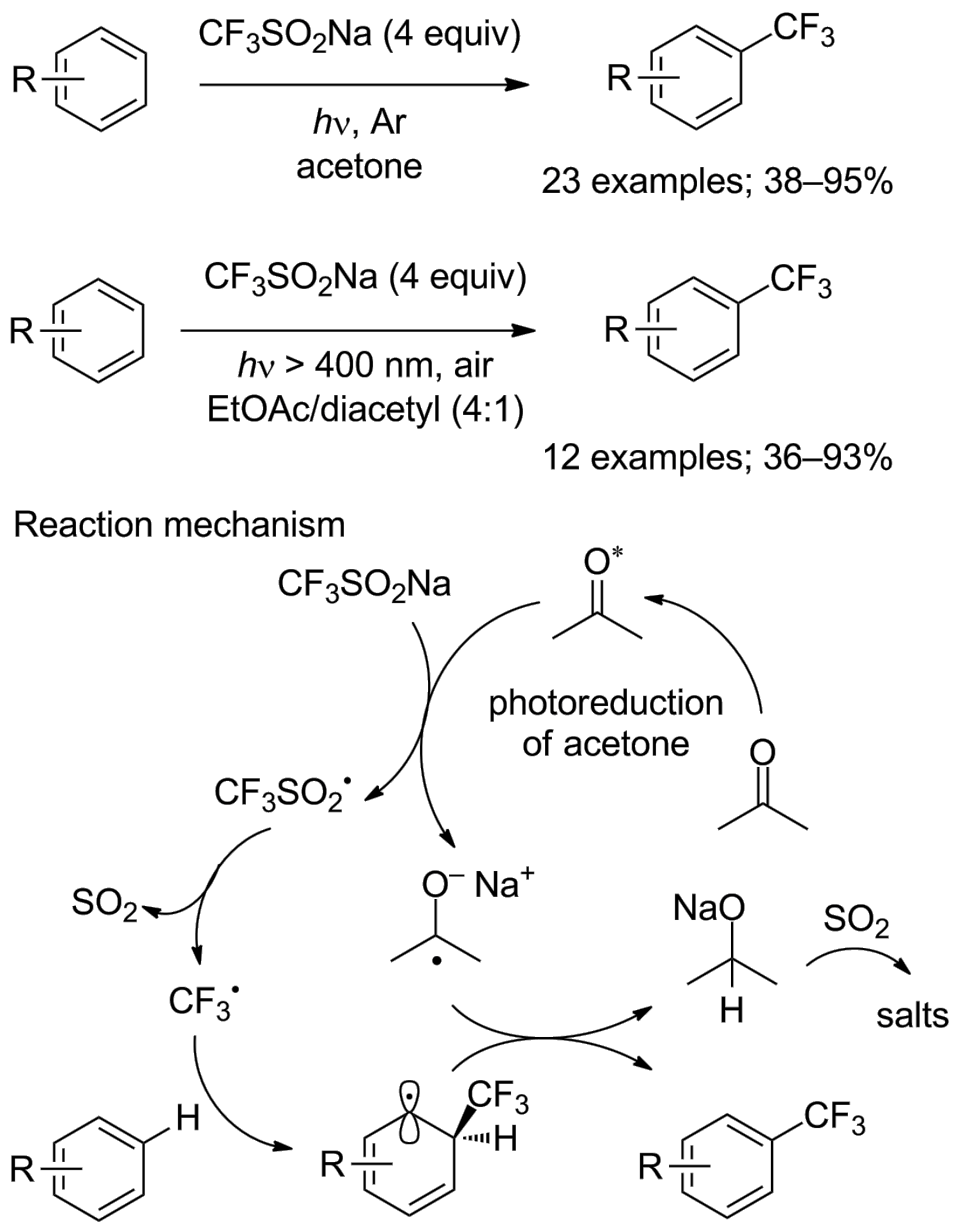
Metal- and oxidant-free photochemical trifluoromethylation of arenes.

In 2016, Li, Mi and co-workers reported a simple and clean approach for the direct trifluoromethylation of unactivated arenes and heteroarenes through a photoreduction without any metal catalyst nor oxidant. The radical initiators were as simple as acetone or diacetyl for the generation of CF_3_ radicals. Indeed, the authors demonstrated that photoexcited acetone was capable to trigger the trifluoromethylation reaction efficiently. So, acetone in this process was used as solvent and photosensitiser. The reactions were carried out under UV irradiation with either a 300 W xenon lamp (emission wavelengths between 200 and 1000 nm) or the photoreactor (λ = 254 nm). This photochemical process allowed the synthesis of trifluoromethylated arenes (Scheme 51), but also heteroarenes and nucleosides in good yields [74]. Further to this work, it should be noted that Davies, MacMillan and co-workers have designed an integrated small-scale photoreactor that enabled acceleration of this photocatalytic reaction [75].

**Scheme 52:**
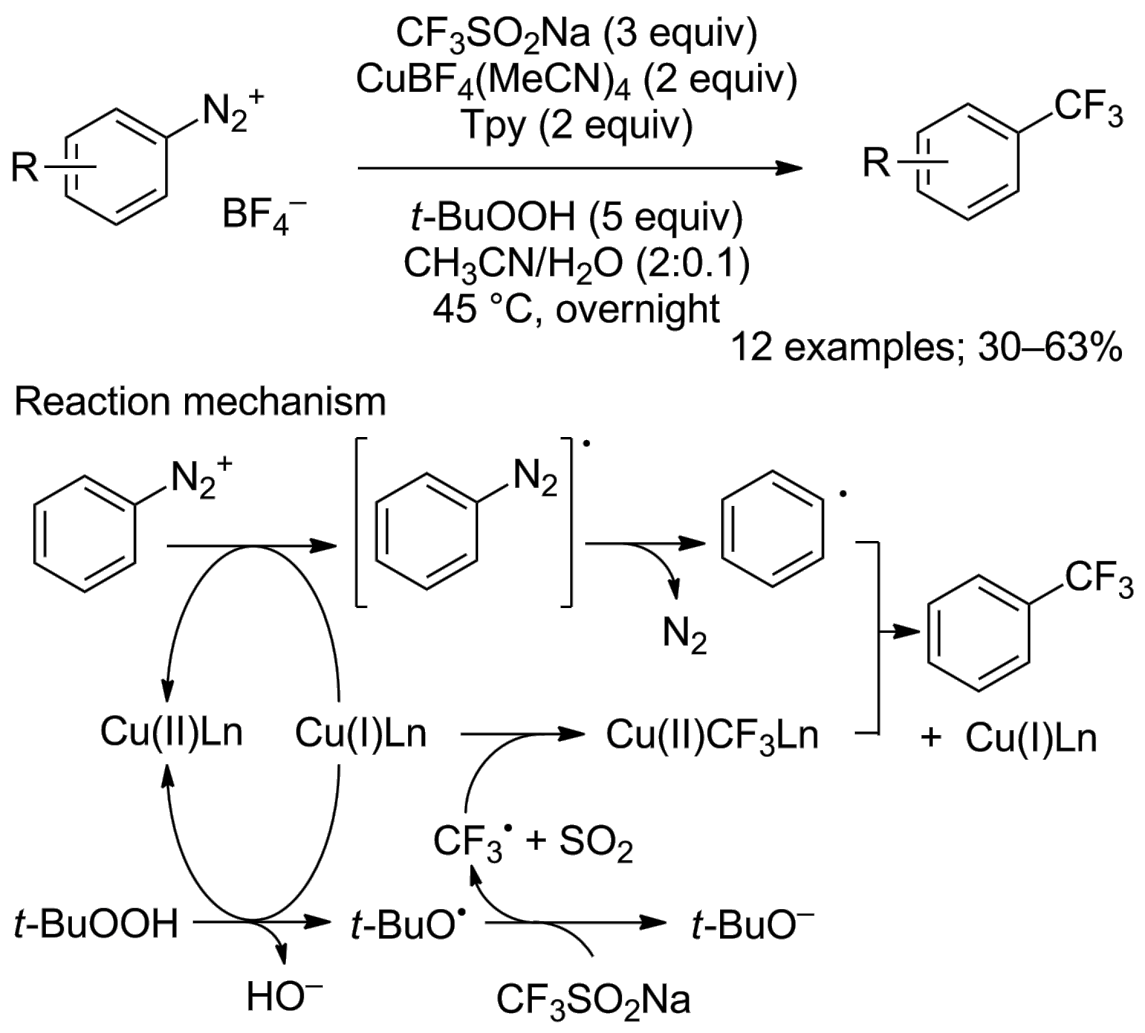
Copper-mediated trifluoromethylation of arenediazonium tetrafluoroborates.

**Trifluoromethylation of arenediazonium compounds:** Langlois’ conditions were applied in the copper-mediated Sandmeyer-type trifluoromethylation of aryldiazonium compounds. The scope of this reaction was investigated on 12 aryldiazonium compounds. The mild reaction conditions allowed the tolerance of various groups such as ester, aryl, nitrile, amine, ketone, nitro, sulfonate and bromo (Scheme 52). In this process, the CF_3_ radical was stabilised by the presence of an excess amount of CuBF_4_(MeCN)_4_ and the tridentate ligand 2,2’;6’,2"-terpyridine (tpy) [76]. Notably, a change in the copper source caused the predominant trifluoromethanesulfonylation of the substrate (introduction of the SO_2_CF_3_ group, vide infra). A one-pot version starting from anilines was recently developed [77].

**Scheme 53:**
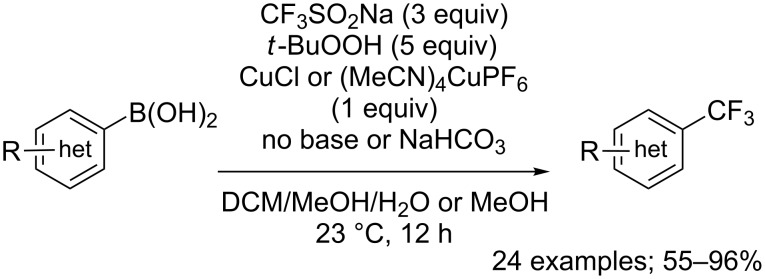
Oxidative trifluoromethylation of aryl- and heteroarylboronic acids.

**Trifluoromethylation of aryl-, vinyl-, alkynylboronic acids:** In 2012, Sanford and co-workers reported, for the first time, the copper-mediated radical trifluoromethylation of aryl- and heteroarylboronic acids using CF_3_SO_2_Na and TBHP as an oxidant. The substrate scope was evaluated on 24 aryl and heteroaryl boronic acids. The process was compatible with various functional groups. Arenes bearing electron-donating groups reacted in excellent yields under the CuCl-mediated conditions. However, arenes with electron-withdrawing substituents necessitated (MeCN)_4_CuPF_6_ and a base, NaHCO_3_, to allow the trifluoromethylation to proceed in good yields (Scheme 53) [78].

**Scheme 54:**
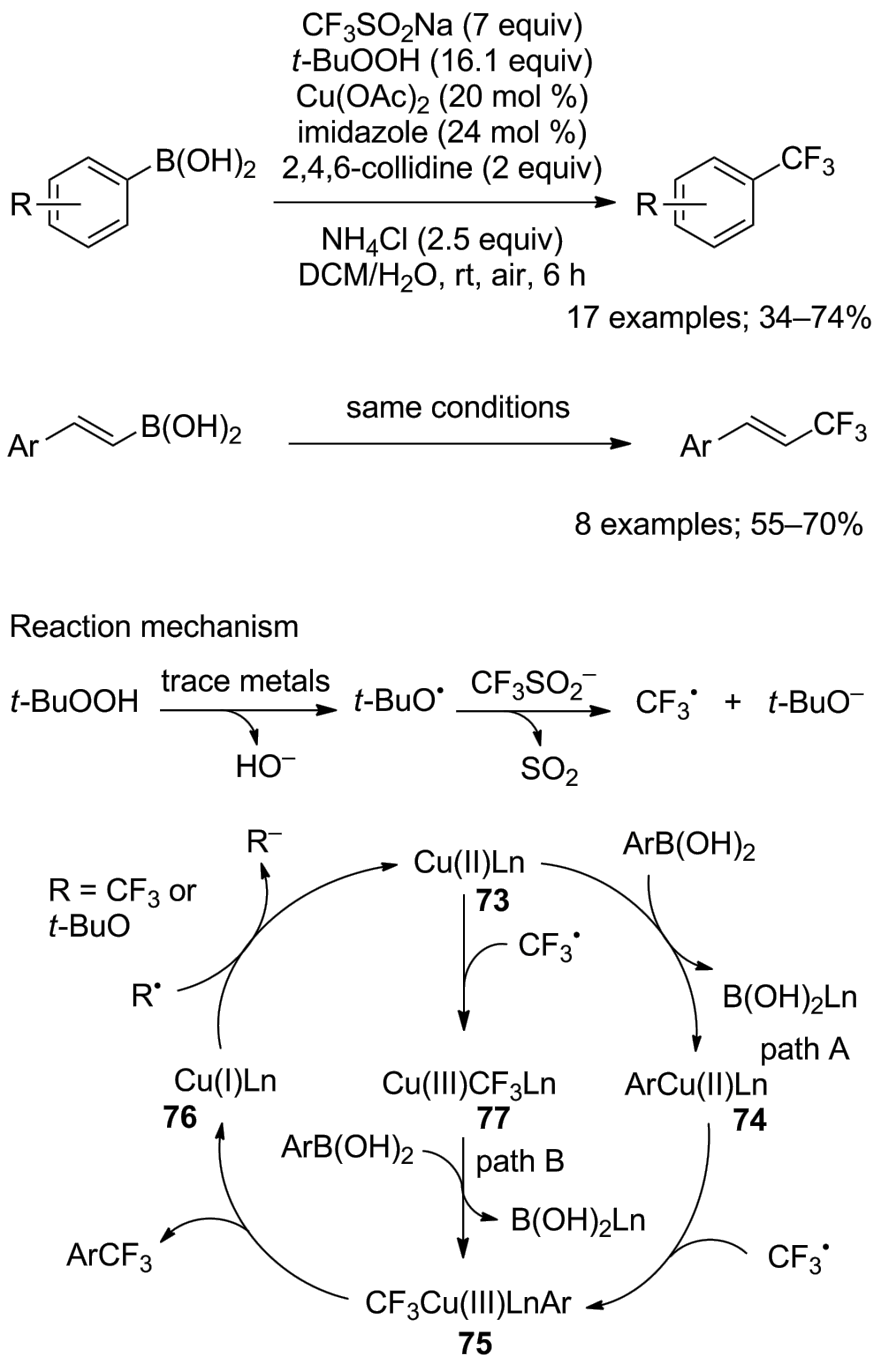
Oxidative trifluoromethylation of aryl- and vinylboronic acids.

In the continuity of Sanford’s work, Beller’s group reported the synthesis of trifluoromethylated arenes from arylboronic acids as well as trifluoromethylated vinylarenes [79]. The substrate scope was realised on 17 arylboronic acids and 8 vinylboronic acids (Scheme 54). The protocol was robust and tolerated various functional groups. However, large excesses of both CF_3_SO_2_Na and TBHP were required. In this process, a ligand, 2,4,6-collidine, was used in order to increase the yield of the transformation. For the styrenylboronic acids, electron-withdrawing and electron-donating substituents on the aryl ring were compatible with the reaction conditions. Based on experimental observations, the authors proposed two possible mechanisms for this trifluoromethylation (Scheme 54). In path A, transmetallation of the boronic acid with the active Cu(II) species **73** gave the arylcopper(II) complex **74**, which reacted with CF_3_^•^ to afford the arylcopper(III) complex **75**. Next, a reductive elimination gave the trifluoromethylated product with release of the Cu(I) complex **76** that was re-oxidised to the active copper(II) catalyst **73** to close the cycle. In path B, the copper(II) complex **73** reacted with CF_3_^•^ to form the copper(III) complex **77**, which after transmetallation with the boronicacid gave the same intermediate **75**.

**Scheme 55:**
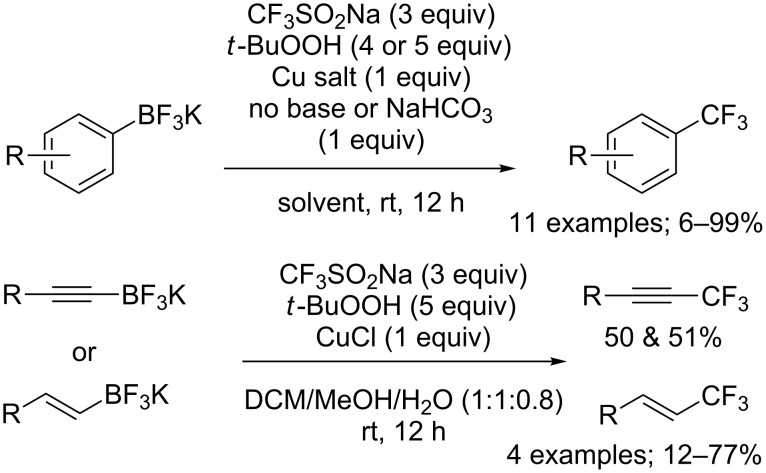
Oxidative trifluoromethylation of unsaturated potassium organotrifluoroborates.

**Trifluoromethylation of potassium organotrifluoroborates:** In 2013, Molander, Rombouts and co-workers simply applied the reaction conditions described by Sanford for boronic acids to a series of unsaturated potassium organotrifluoroborates (Scheme 55) [80]. In the case of aryl- and heteroaryltrifluoroborates, electron-rich substrates were efficiently trifluoromethylated although the increase of the steric hindrance caused a decrease in yields. On the other hand, the yields obtained with electron-poor potassium organotrifluoroborates were lower. In the same paper, the preparation of trifluoromethyl-substituted alkynes and alkenes from alkynyl- and alkenyltrifluoroborates was demonstrated albeit in moderate yields. The yields of the trifluoromethylation of alkenyltrifluoroborates strongly depended on the substitution of the olefin (Scheme 55).

**Scheme 56:**
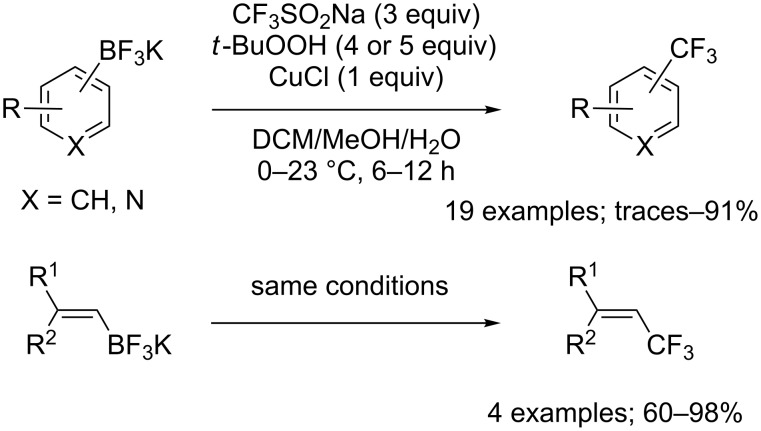
Oxidative trifluoromethylation of (hetero)aryl- and vinyltrifluoroborates.

Simultaneously, Dubbaka and co-workers reported the trifluoromethylation of aryl-, heteroaryl-, and vinyltrifluoroborates with CF_3_SO_2_Na (Scheme 56) [81]. The authors used basically the same protocol as Sanford and Molander but observed that a more diluted reaction medium gave improved reaction yields.

**Scheme 57:**
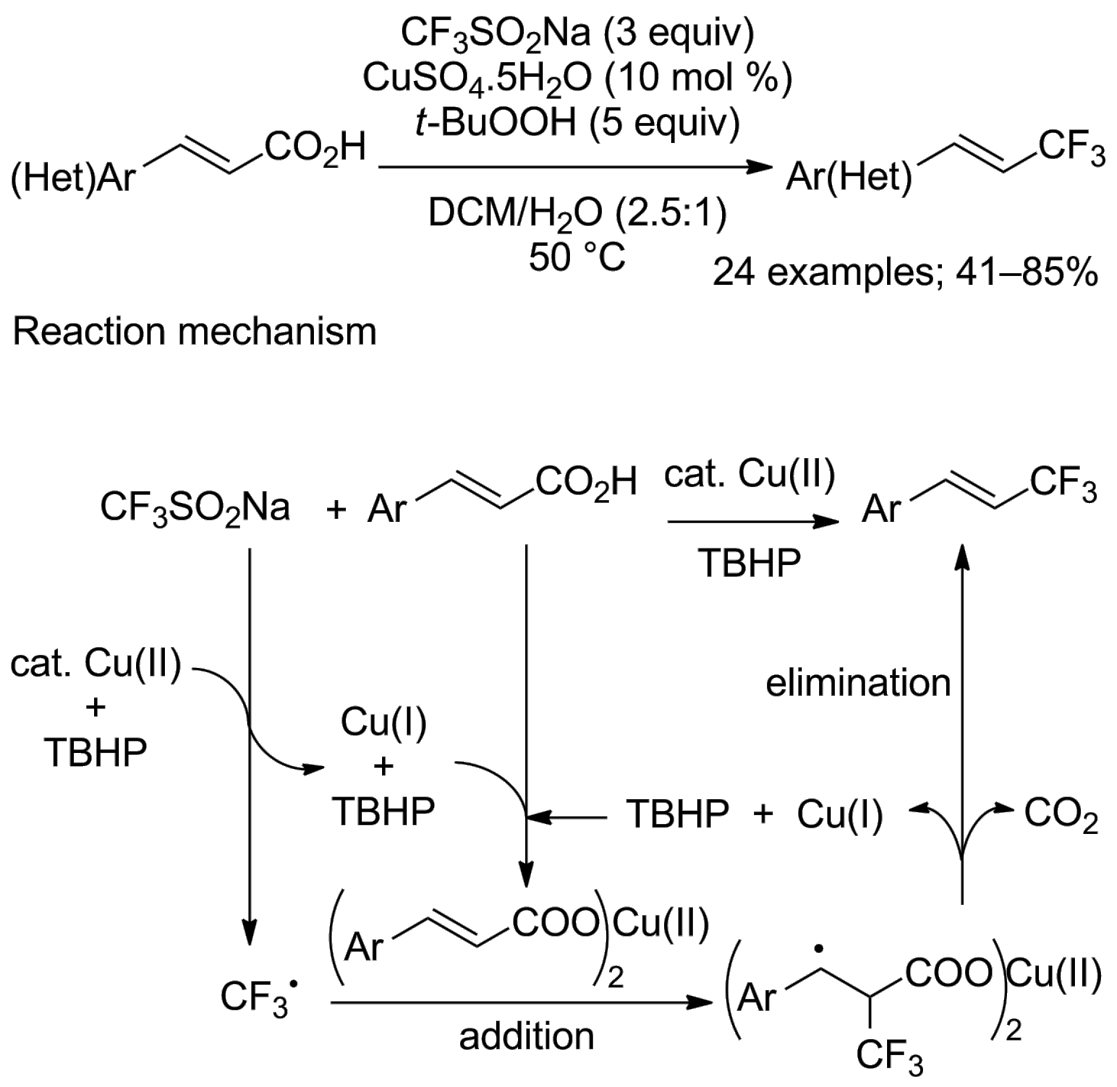
Copper−catalysed decarboxylative trifluoromethylation of cinnamic acids.

**Trifluoromethylation of alkenes by decarboxylation:** Liu and co-workers were the first to describe the copper-catalysed decarboxylative trifluoromethylation of α,β-unsaturated carboxylic acids in the presence of CF_3_SO_2_Na and TBHP [82]. Various (hetero)arenes were compatible with these reaction conditions (Scheme 57); nevertheless, cinnamic acids bearing electron-donating groups afforded the corresponding products in better yields. The stereoselectivity was moderate to high from 76:24 to 99:1. Interestingly, aryls bearing a nitro or a chloro substituent gave the corresponding α-trifluoromethyl ketones. This process was interesting for the construction of C_vinyl_–CF_3_ bonds via a radical addition–elimination (Scheme 57).

**Scheme 58:**
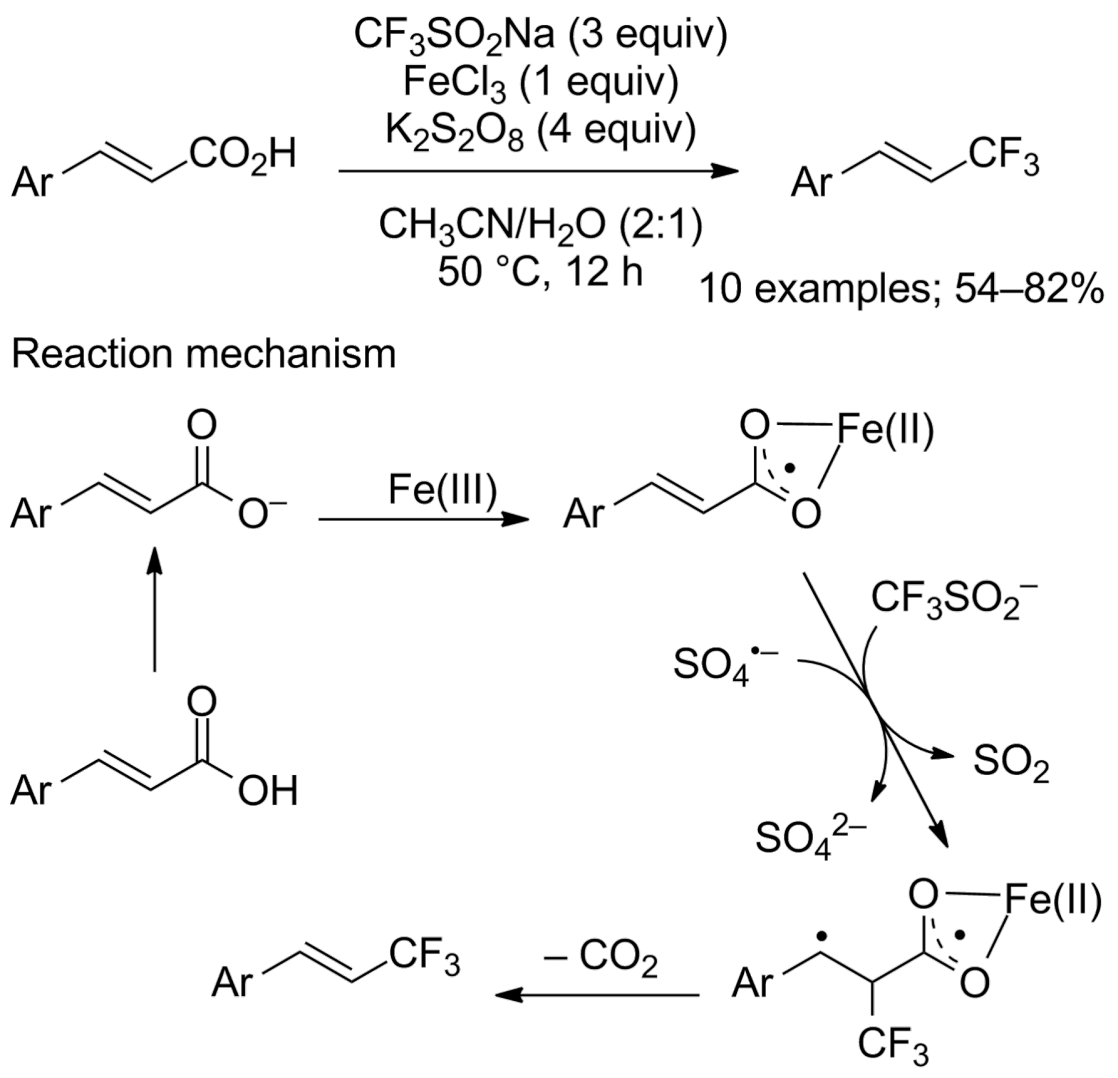
Iron-mediated decarboxylative trifluoromethylation of α,β-unsaturated carboxylic acids.

Soon after, Maiti and co-workers reported an iron-mediated trifluoromethylation of α,β-unsaturated carboxylic acids with CF_3_SO_2_Na, iron(III) chloride and potassium persulfate as oxidant [83]. The substrate scope was evaluated on 10 cinnamic acids (Scheme 58). Under these conditions, both electron-rich or electron-poor arenes gave the vinylic CF_3_ products in good yields and with excellent stereoselectivities. A mechanism was proposed starting from the generation of iron carboxylate and the reaction with the CF_3_ radical followed by extrusion of CO_2_ and radical coupling (Scheme 58).

**Scheme 59:**
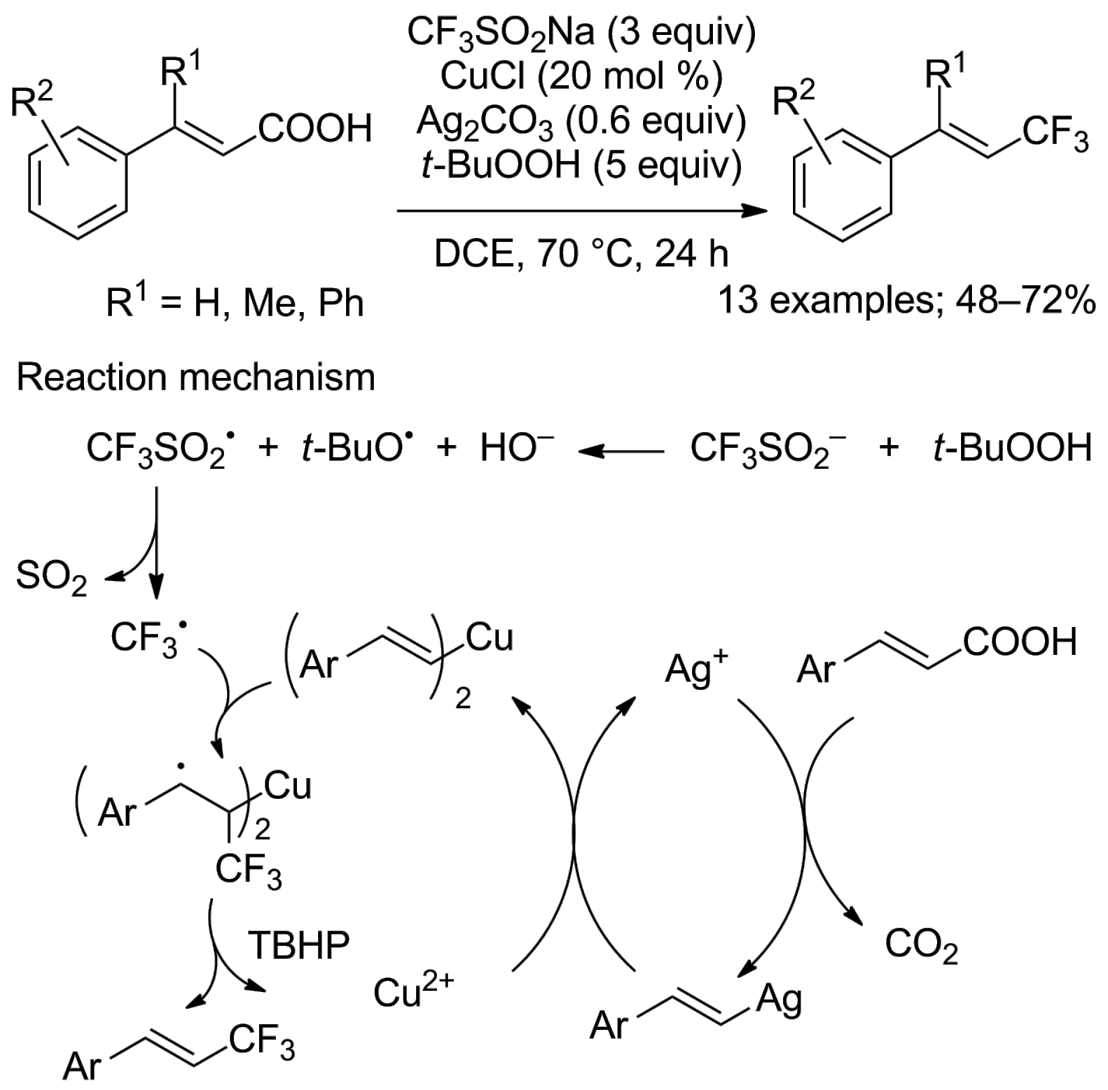
Cu/Ag-catalysed decarboxylative trifluoromethylation of cinnamic acids.

Two major drawbacks appeared in the above-mentioned works: halo-substituted aryl derivatives failed to give the expected products and α,β-unsaturated carboxylic acids substituted at the β-position were not studied. Accordingly, Li, Duan and co-workers reported a copper/silver-catalysed decarboxylative trifluoromethylation of α,β-unsaturated carboxylic acids with CF_3_SO_2_Na that tolerated various substrates bearing halogens as well as α,β-unsaturated carboxylic acids substituted at the β-position (Scheme 59) [84]. It was even noticed that β-methyl or β-phenyl-substituted cinnamic acids gave better yields compared to unsubstituted cinnamic acid. All products were obtained with excellent stereoselectivity. To gain insight about the mechanism, the authors used TEMPO as a radical scavenger and concluded that CF_3_ radical was involved in this reaction (Scheme 59).

**Scheme 60:**
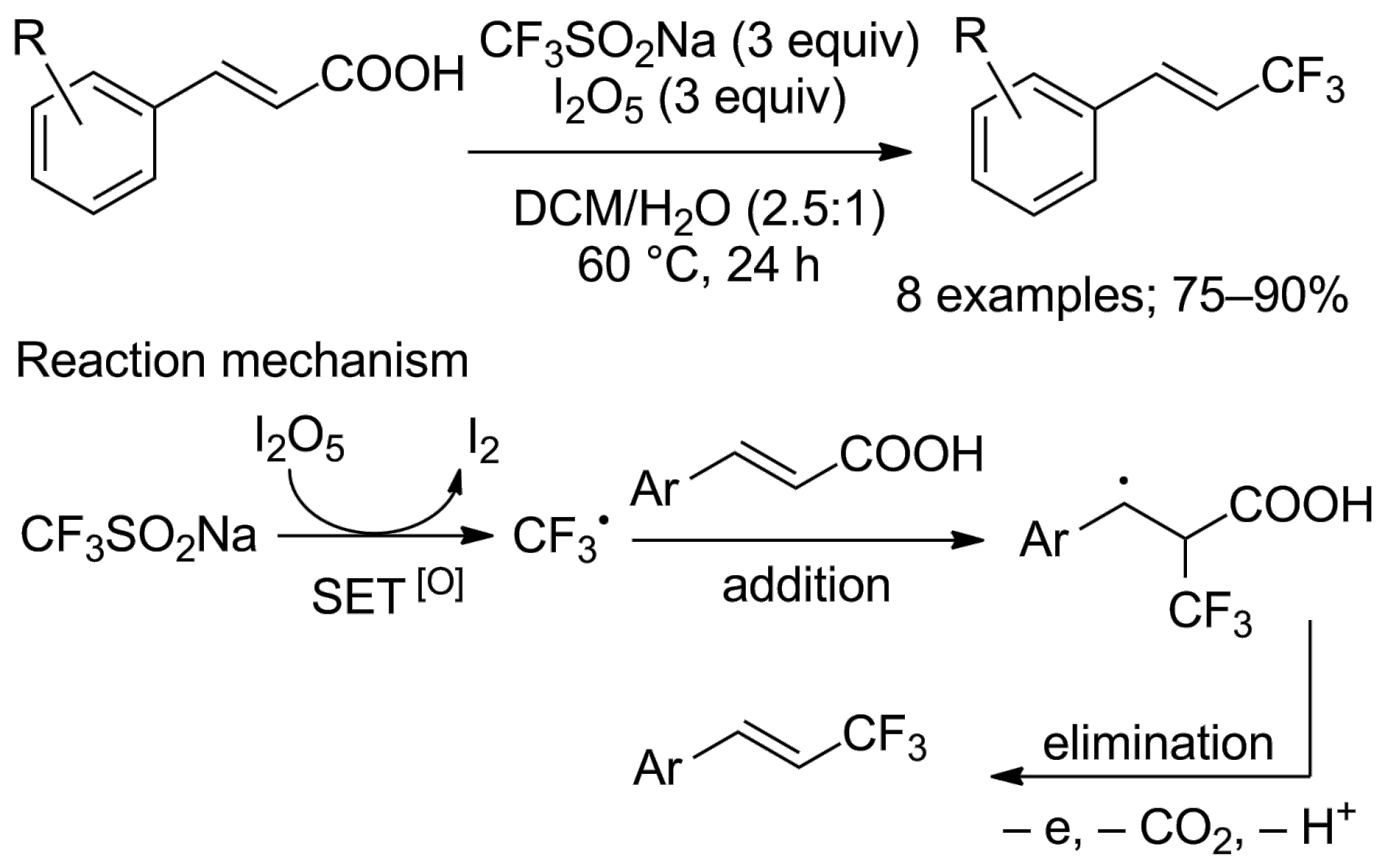
I_2_O_5_-Promoted decarboxylative trifluoromethylation of cinnamic acids.

A metal-free protocol for the decarboxylative trifluoromethylation of cinnamic acids with CF_3_SO_2_Na was reported by Shang, Liu and co-workers using iodine pentoxide (I_2_O_5_) as oxidant. The substrate scope was evaluated on 9 α,β-unsaturated carboxylic acids substituted by electron-rich aryls (Scheme 60) [85]. The mild conditions gave the trifluoromethylated products in excellent yields with high stereoselectivities. Nevertheless, the reaction conditions did not allow the formation of products with electron-withdrawing substituents on the aromatic ring, such as the nitro group. The case of halogen-substituted cinnamic acids was not studied. Finally, mechanistic studies conducted by electron-spin resonance and by spin trapping technology suggested a free-radical addition– elimination process (Scheme 60).

**Scheme 61:**
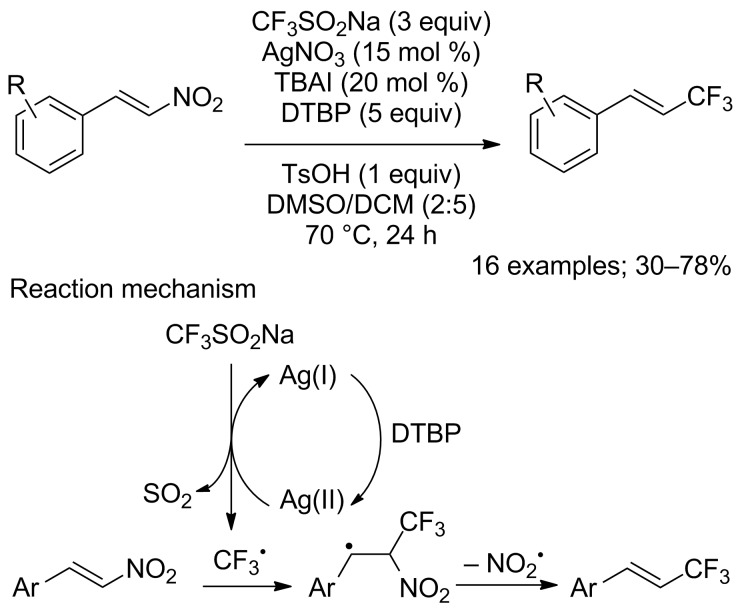
Silver(I)-catalysed denitrative trifluoromethylation of β-nitrostyrenes.

**Trifluoromethylation of alkenes by denitration:** Another route to the C_vinyl_–CF_3_ bond is the denitrative trifluoromethylation of β-nitrostyrenes. More generally, this transformation has attracted much attention as a useful method for the construction of C_vinyl_–R moieties. In 2016, Li, Duan and co-workers reported this trifluoromethylation by means of CF_3_SO_2_Na catalysed by silver nitrate in the presence of a large excess of di-*tert*-butyl peroxide (DTBP) as the oxidant and tetrabutylammonium iodide (TBAI) as a phase-transfer catalyst (Scheme 61) [86]. The substrate scope was evaluated on 16 β-nitrostyrenes and substrates bearing electron-donating groups as well as halides afforded the corresponding trifluoromethylstyrenes in moderate to good yields. However, the substrates with electron-withdrawing groups (CN, NO_2_) led to trifluoromethylated products in poor yields. This transformation was highly stereoselective, only (*E*)-isomers of the products were obtained. Experiments in the presence of 1,1-diphenylethylene as a radical scavenger led the authors to propose a radical process for the trifluoromethylation as described in Scheme 61.

**Scheme 62:**
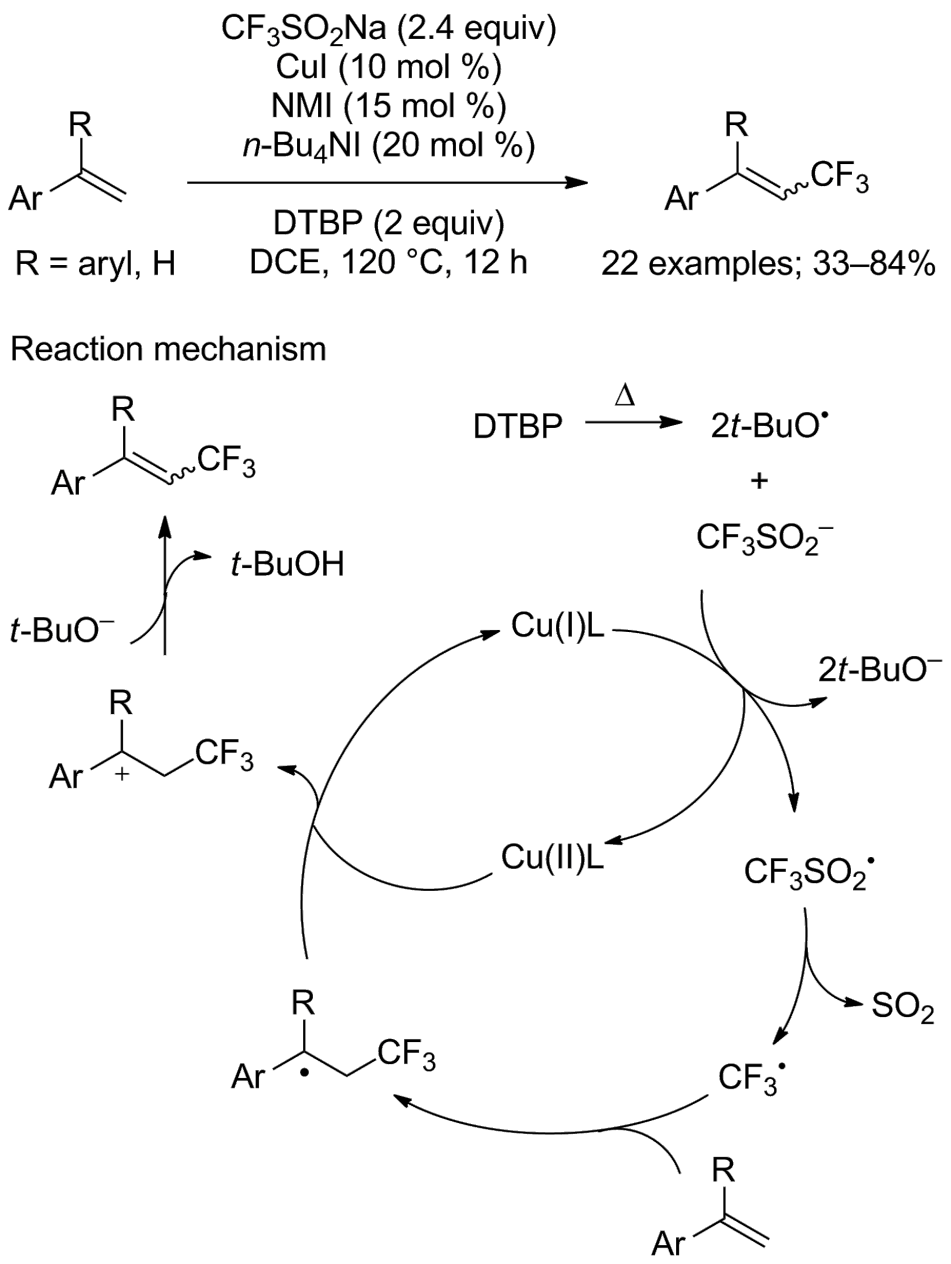
Copper-catalysed direct trifluoromethylation of styrene derivatives.

**Trifluoromethylation of styrene derivatives:** An obviously simple route for the synthesis of trifluoromethylated styrene derivatives is the direct C–H trifluoromethylation of alkenes. Very recently, Shen, Loh and co-workers reported a copper-catalysed direct trifluoromethylation of the vinylic C_sp2_–H bond of styrene derivatives with CF_3_SO_2_Na, di-*tert*-butyl peroxide, 10 mol % of copper(I) iodide, 1-methylimidazole (NMI) as ligand to copper and tetrabutylammonium iodide (TBAI) as additive (Scheme 62) [87]. The mild reaction conditions allowed a wide variety of functional groups to be tolerated and afforded a series of trifluoromethylated styrenes in moderate to good yields and with excellent stereoselectivity. However, aliphatic alkenes or styrenes bearing electron-withdrawing groups were not suitable for this reaction. Based on control experiments, the authors proposed a radical pathway for this reaction (Scheme 62).

**Scheme 63:**
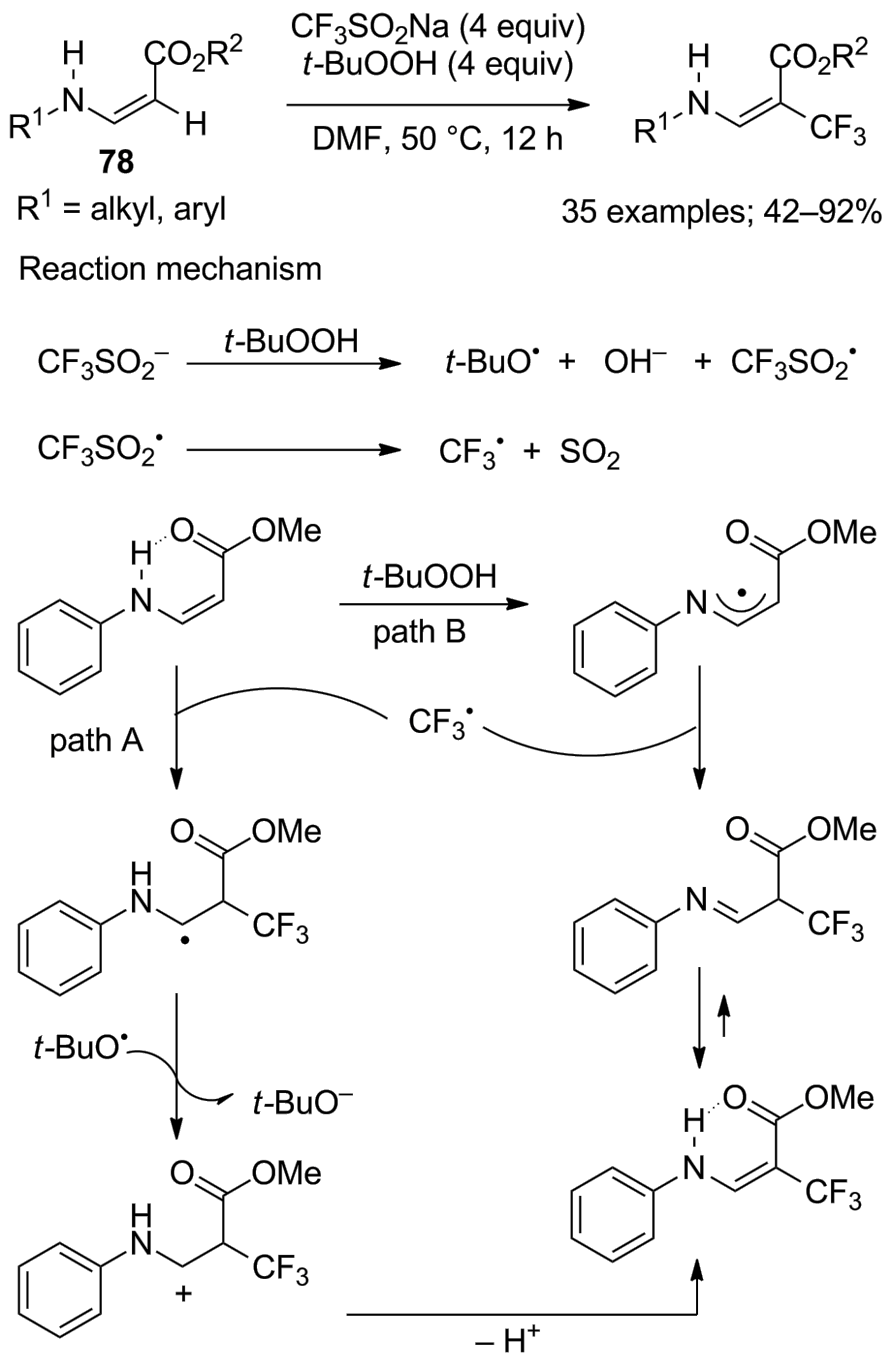
Transition-metal-free synthesis of β-trifluoromethylated enamines.

**Trifluoromethylation of enamines:** Highly functionalised alkenes represented by (*Z*)-methyl-3-(phenylamino)acrylates **78** were subjected to Baran’s conditions for the synthesis of β-trifluoromethylated enamines. Indeed, Jiang, Wu and co-workers reported mild, transition-metal-free conditions, insensitive to air and water, for the synthesis of a wide range of CF_3_-enamines using CF_3_SO_2_Na and TBHP as initiator and oxidant (Scheme 63) [88]. Moderate to good yields were obtained and only (*E*)-isomers were observed. The authors carried out several experiments such as the use of TEMPO or 2,6-di-*tert*-butyl-4-methylphenol (BHT) as scavengers in order to investigate the mechanism of this reaction (Scheme 63).

**Scheme 64:**
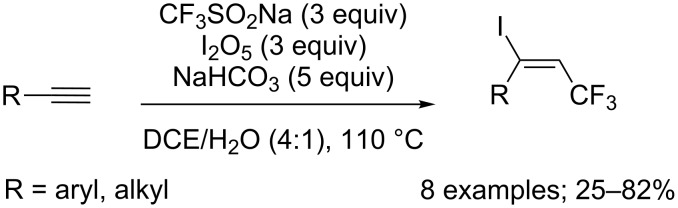
I_2_O_5_-mediated iodotrifluoromethylation of alkynes.

**Trifluoromethylation of alkynes:** Together with the iodotrifluoromethylation of alkenes leading to C_sp3_–CF_3_ products (see Scheme 22), the Liu group extended the free-radical iodotrifluoromethylation to alkynes with the combination of CF_3_SO_2_Na/I_2_O_5_ and assistance of NaHCO_3_ (Scheme 64) [42]. The substrate scope was carried out on various aryl-substituted alkynes and one propargylic ester; (*E*)-CF_3_ alkenyl iodides were obtained stereoselectively in moderate to high yields via a free-radical process.

**Scheme 65:**
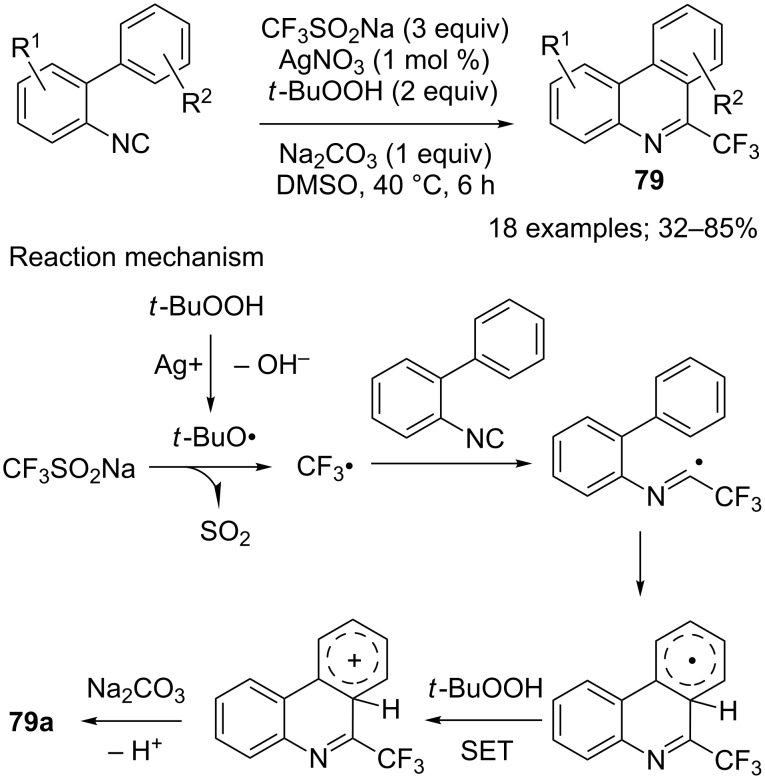
Silver-catalysed tandem trifluoromethylation/cyclisation of aryl isonitriles.

**Trifluoromethylation of isonitriles:** The synthesis of trifluoromethylated phenanthridines **79** from aryl isonitriles has been the recent subject of several investigations as potential structural unit in pharmaceuticals. In this context, CF_3_SO_2_Na was used by Zhang and co-workers in a silver-catalysed tandem trifluoromethylation and cyclisation of aryl isonitriles (Scheme 65) [89]. A wide variety of aryl isocyanides were transformed into the corresponding phenanthridines in moderate to good yields; some regioisomers were obtained depending on the biphenyl substituents. Here again, a radical pathway was established for this transformation (Scheme 65).

**Scheme 66:**
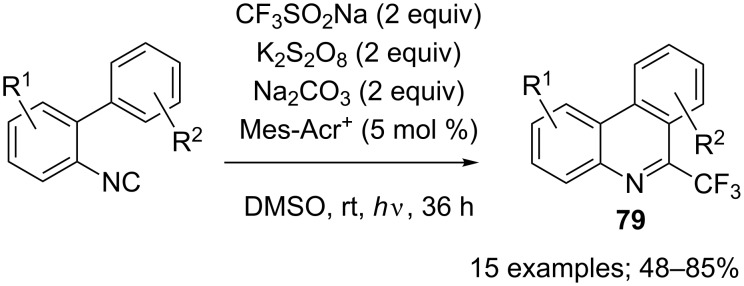
Photoredox trifluoromethylation of 2-isocyanobiphenyls.

Simultaneously, Lu and co-workers reported a transition-metal-free synthesis of the trifluoromethylphenanthridine **79a** in 58% yield using the system CF_3_SO_2_Na/K_2_S_2_O_8_/K_2_CO_3_ in H_2_O/CH_3_CN at 80 °C [90]. In addition, Maruoka and co-workers used the system CF_3_SO_2_Na/PIFA/AcONa in AcOEt at room temperature for 1.5 h to get **79a** in 74% yield [50]. Mid 2017, Ao, Liu and co-workers exploited the reaction conditions developed previously for the photoredox trifluoromethylation of vinyl azides (see Scheme 8) in the synthesis of fluorinated phenanthridines **79**. The yields were moderate to good and regioselectivity issues appeared depending on the biphenyl substituents (Scheme 66) [91].

#### C_sp_–CF_3_ bond-forming reactions

**Scheme 67:**
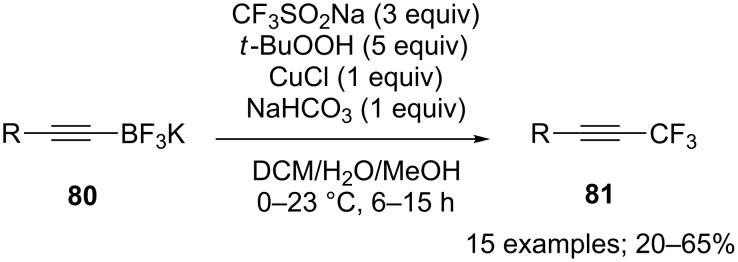
Trifluoromethylation of potassium alkynyltrifluoroborates with CF_3_SO_2_Na.

For the synthesis of trifluoromethylacetylenes **81**, a copper-mediated trifluoromethylation of potassium alkynyltrifluoroborates **80** with CF_3_SO_2_Na was developed by Dubbaka and co-workers (Scheme 67) [92]. The scope was large including aryl, heteroaryl, alkenyl, and aliphatic alkynyltrifluoroborates and the yields were moderate. Mechanistically, the CF_3_ radical was generated under Langlois’ conditions by means of *tert*-butyl hydroperoxide and CuCl.

#### N–CF_3_ bond-forming reactions

**Scheme 68:**
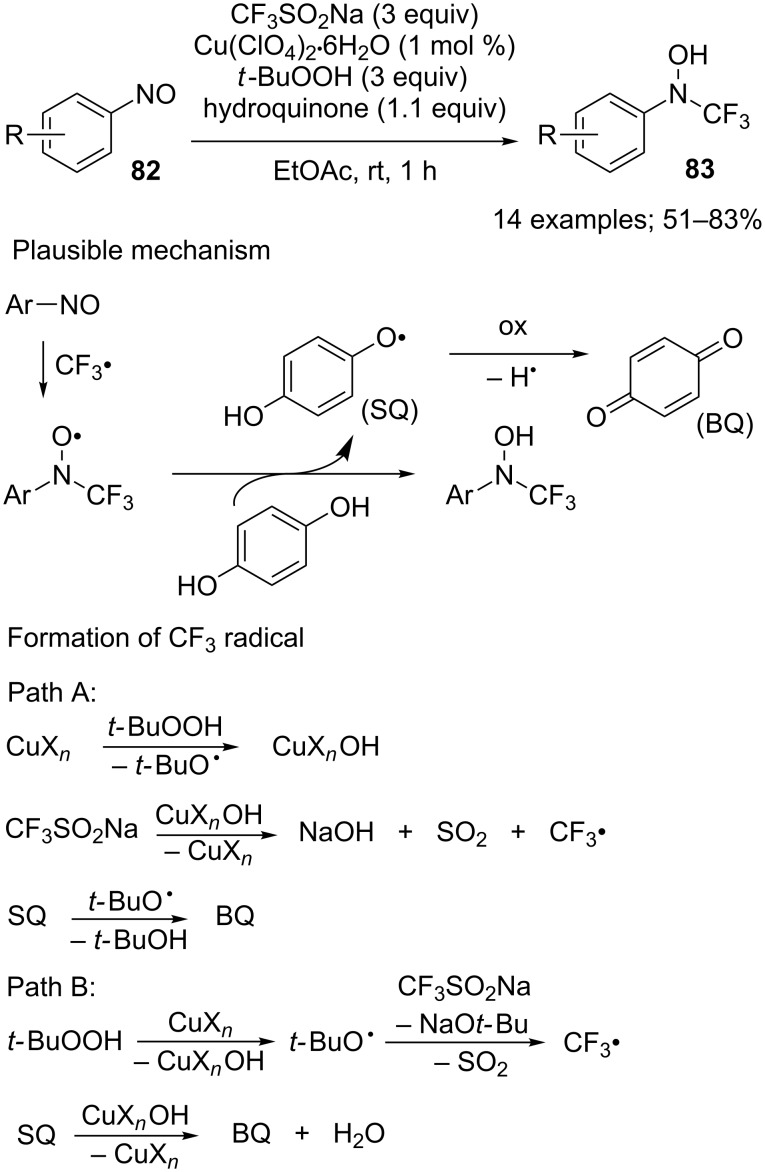
*N*-trifluoromethylation of nitrosoarenes with CF_3_SO_2_Na (SQ: semiquinone).

C-, O-, and S-trifluoromethylated compounds are common in the field of biologically active molecules unlike the N–CF_3_ motif despite the huge number of nitrogen-containing pharmaceuticals. In 2017, Selander and co-workers reported a chemoselective *N-*trifluoromethylation of nitrosoarenes **82** in the presence of CF_3_SO_2_Na, a catalytic amount of copper(II), *tert*-butyl hydroperoxide as oxidant and hydroquinone as additive [93]. No reaction was observed in the absence of the copper salt. The scope was evaluated on 14 nitrosoarenes featuring electron-poor and electron-rich groups. The reaction conditions were suitable with a wide variety of functionalised nitrosoarenes and the corresponding trifluoromethylated hydroxylamines **83** were obtained in moderate to good yields (Scheme 68). The authors proposed a radical mechanism with two pathways for the generation of the CF_3_ radical, either by copper species or by *tert-*BuO^•^ (Scheme 68). Interestingly, N–CF_3_ anilines were easily obtained after reduction of the N–O bond.

#### S–CF_3_ bond-forming reactions

**Scheme 69:**
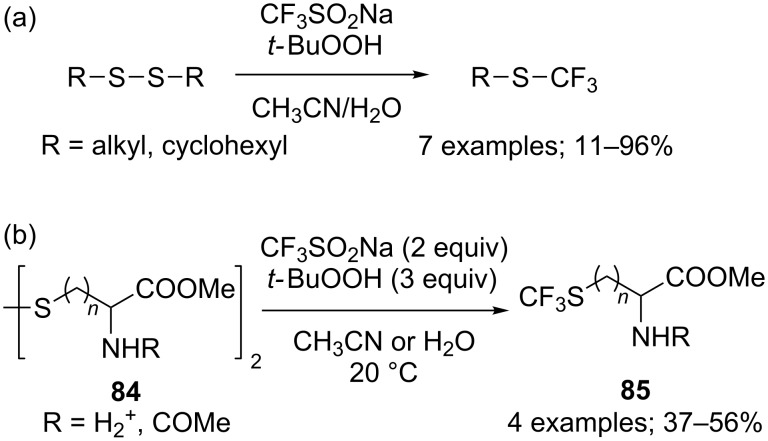
Trifluoromethylation of disulfides with CF_3_SO_2_Na.

Synthetic routes to trifluoromethylthiolated compounds are diverse, one is the S–CF_3_ bond formation (see next section for C–SCF_3_ bond formation). For this purpose, Langlois and co-workers used aliphatic and aromatic disulfides, RS–SR, which reacted with CF_3_SO_2_Na in the presence of an oxidant, *t-*BuOOH providing the best yields, to afford the trifluoromethyl thioethers (Scheme 69a) [20]. Other oxidants such as K_2_S_2_O_8_ and (NH_4_)_2_Ce(NO_3_)_6_ displayed lower reactivity and selectivity. Only one sulfenyl moiety of the disulfide was trifluoromethylated in this approach, the second being oxidised without trifluoromethylation. Aryl disulfides exhibited a lower reactivity and selectivity because side radical aryl C–H trifluoromethylation occurred competitively. This approach was applied to the synthesis of *S*-trifluoromethyl-containing amino acids **85** from dithio-amino acids **84** (Scheme 69b) [94].

**Scheme 70:**
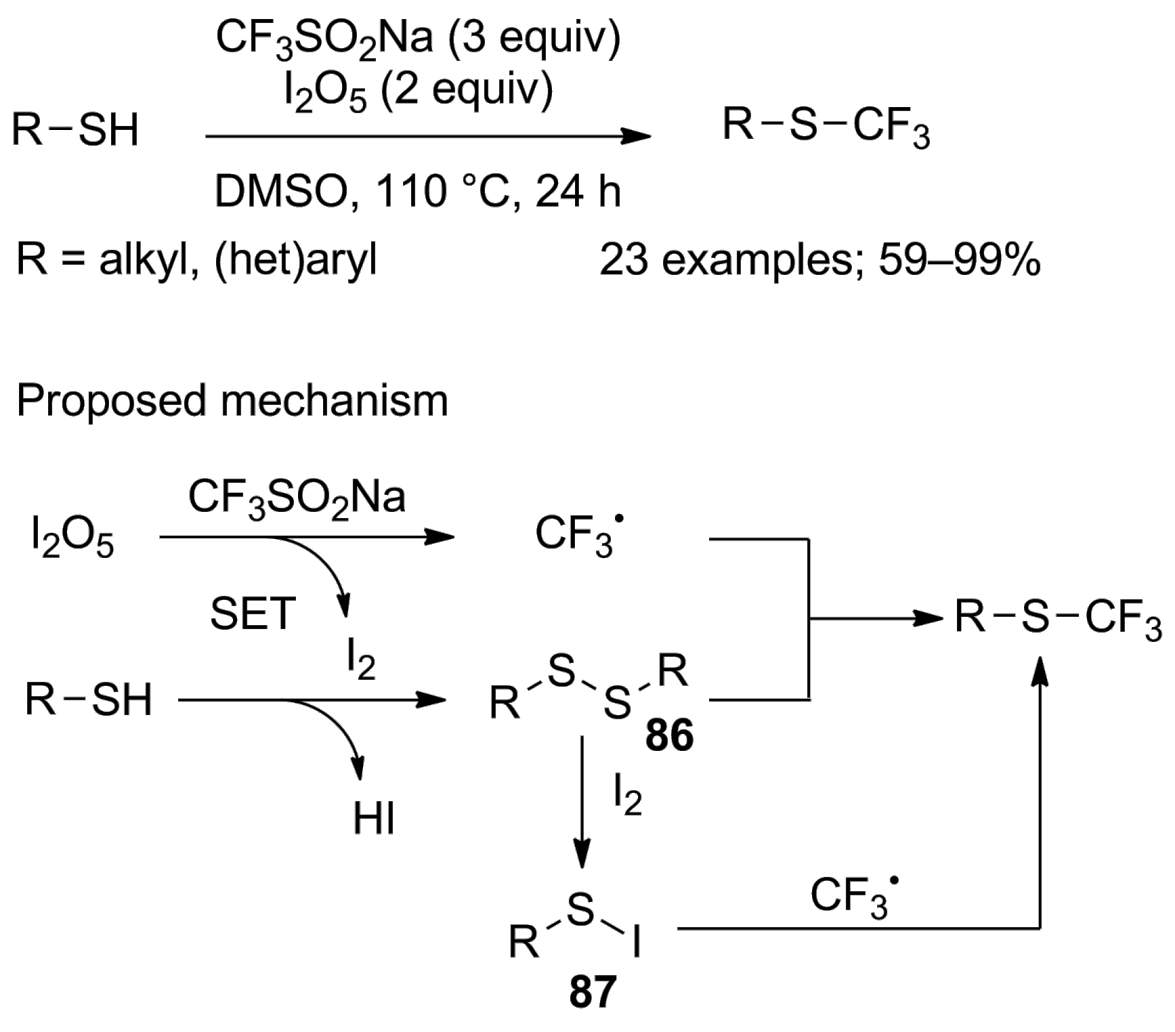
Trifluoromethylation of thiols with CF_3_SO_2_Na/I_2_O_5_.

Apart from disulfides, thiols were also used as substrates by Yi and co-workers to produce several trifluoromethyl thioethers and aliphatic trifluoromethylthiols. Their contribution was inspired by the previous work of Liu, using iodine pentoxide as an inexpensive inorganic oxidant [42] to generate the CF_3_ radical and release of iodine, which then reacted with the thiol to first form the corresponding disulfide **86** (detected as reaction intermediate) and further the sulfenyl iodide **87**. The final SCF_3_ product was the result of the reaction of CF_3_^•^ with either **86** or/and **87** (Scheme 70) [95]. The reaction performed well only at high reaction temperature (110 °C) for both thiophenols, benzylthiols and mercapto derivatives. The same research group recently reported different reaction conditions to obtain the same SCF_3_ products by means of CF_3_SO_2_Na in the presence of potassium persulfate and a catalytic amount of silver nitrate at 80 °C in acetonitrile and water [96].

### 2 Trifluoromethylsulfenylation

**Scheme 71:**
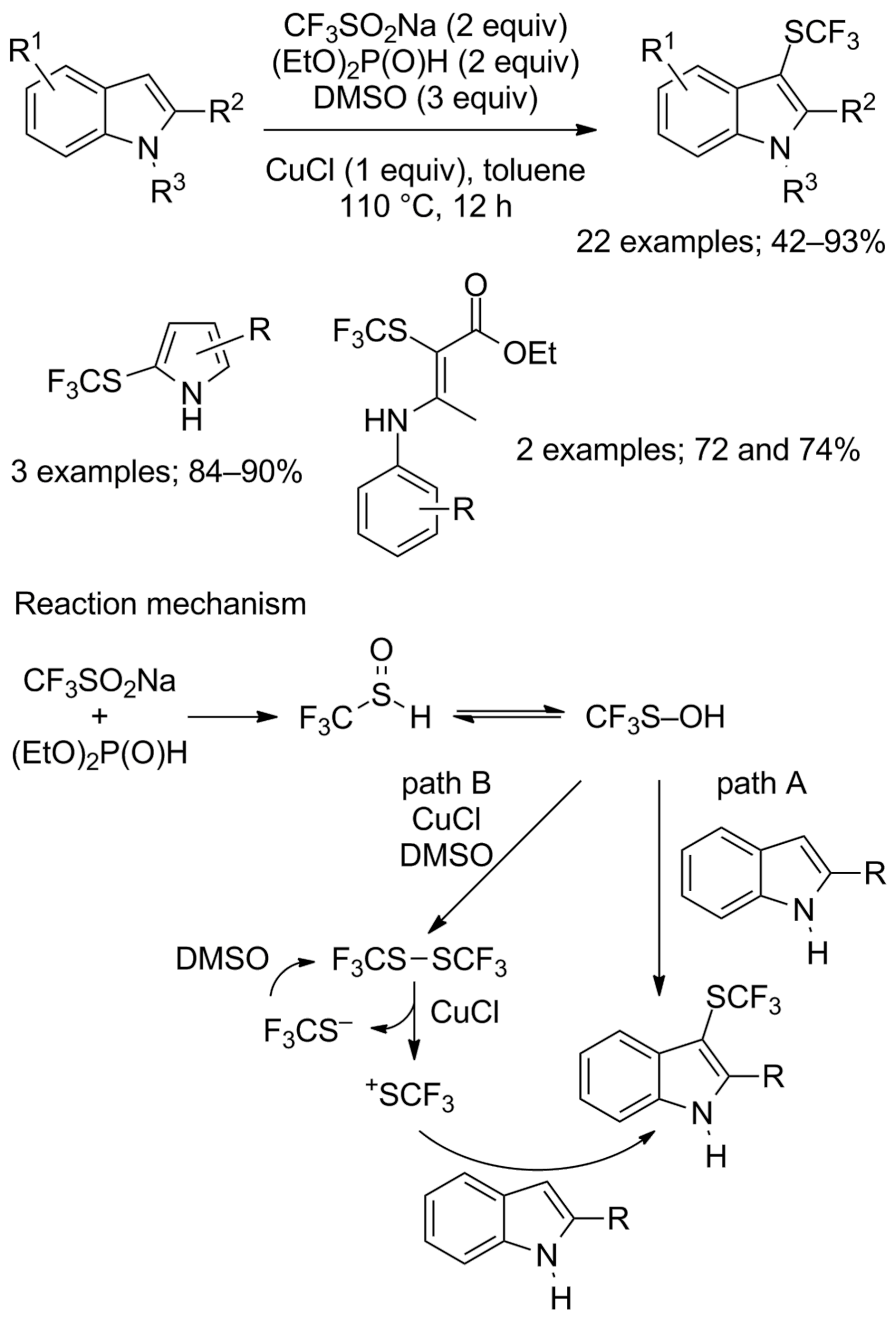
Electrophilic trifluoromethylsulfenylation by means of CF_3_SO_2_Na/(EtO)_2_P(O)H/CuCl/DMSO.

In the early development of the direct electrophilic trifluoromethylthiolation, trifluoromethanethiol (CF_3_SH), trifluoromethanesulfenyl chloride (CF_3_SCl), and bis(trifluoromethyl) disulfide (CF_3_SSCF_3_) were the only reagents available, but their gaseous and toxic nature precluded a wider use. Fortunately, in the recent years, a collection of stable and easy-to-handle reagents was designed to perform efficient trifluoromethylthiolations [97]. Less sophisticated and suitable for large scale industrial use, CF_3_SO_2_Na was first used in trifluoromethylthiolation reactions in 2015 by Yi, Zhang and co-workers who reported the direct copper-catalysed trifluoromethylthiolation of indoles, pyrroles as well as enamines in the presence of (EtO)_2_P(O)H as reductant, CuCl and DMSO in toluene at 110 °C (Scheme 71) [98]. Unlike its use in trifluoromethylation reactions, here, no extrusion of SO_2_ ocurred from CF_3_SO_2_Na thus allowing the transfer of the SCF_3_ motif. Under these reaction conditions, indoles bearing an electron-donating group gave better yields compared to those with electron-rich groups. Using the same protocol, the trifluoromethylsulfenylated pyrroles and enamines were obtained in good to excellent yields. After mechanistic studies by ^19^F NMR spectroscopy, the authors proposed that bis(trifluoromethyl) disulfide, CF_3_SSCF_3_, was generated in situ (Scheme 71, path B). The route through path A and CF_3_SOH was also envisaged but should lead only to the formation of small amount of product.

**Scheme 72:**
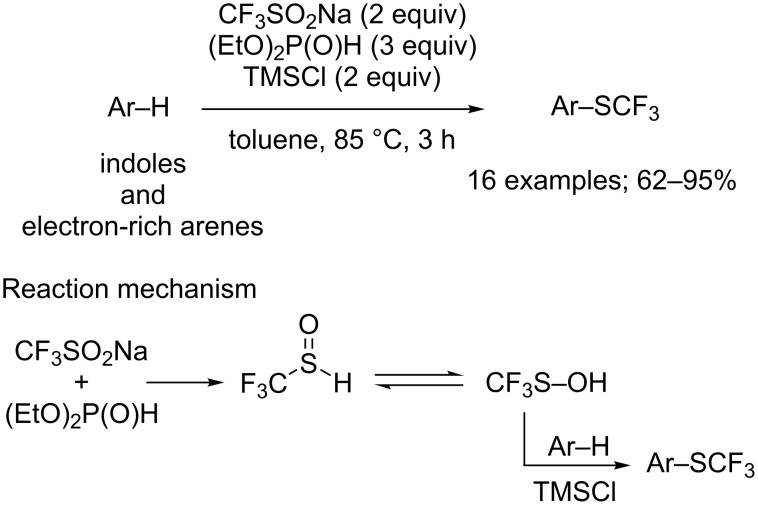
Electrophilic trifluoromethylsulfenylation by means of CF_3_SO_2_Na/(EtO)_2_P(O)H/TMSCl.

The same authors further demonstrated that the trifluoromethylsulfenylation can be conducted under metal-free conditions by replacing CuCl with trimethylsilyl chloride (TMSCl) to generate the cation CF_3_S^+^ in the presence of (EtO)_2_P(O)H [99]. Again, the electrophilic trifluoromethylsulfenylation of indoles was studied but also the trifluoromethylthiolation of electron-rich arenes (Scheme 72). Using this protocol, trifluoromethylsulfenylated compounds were obtained in moderate to excellent yields. Compared to the CuCl-mediated approach, this method directly converted CF_3_SOH into CF_3_S^+^ by reaction with TMSCl (Scheme 72).

**Scheme 73:**
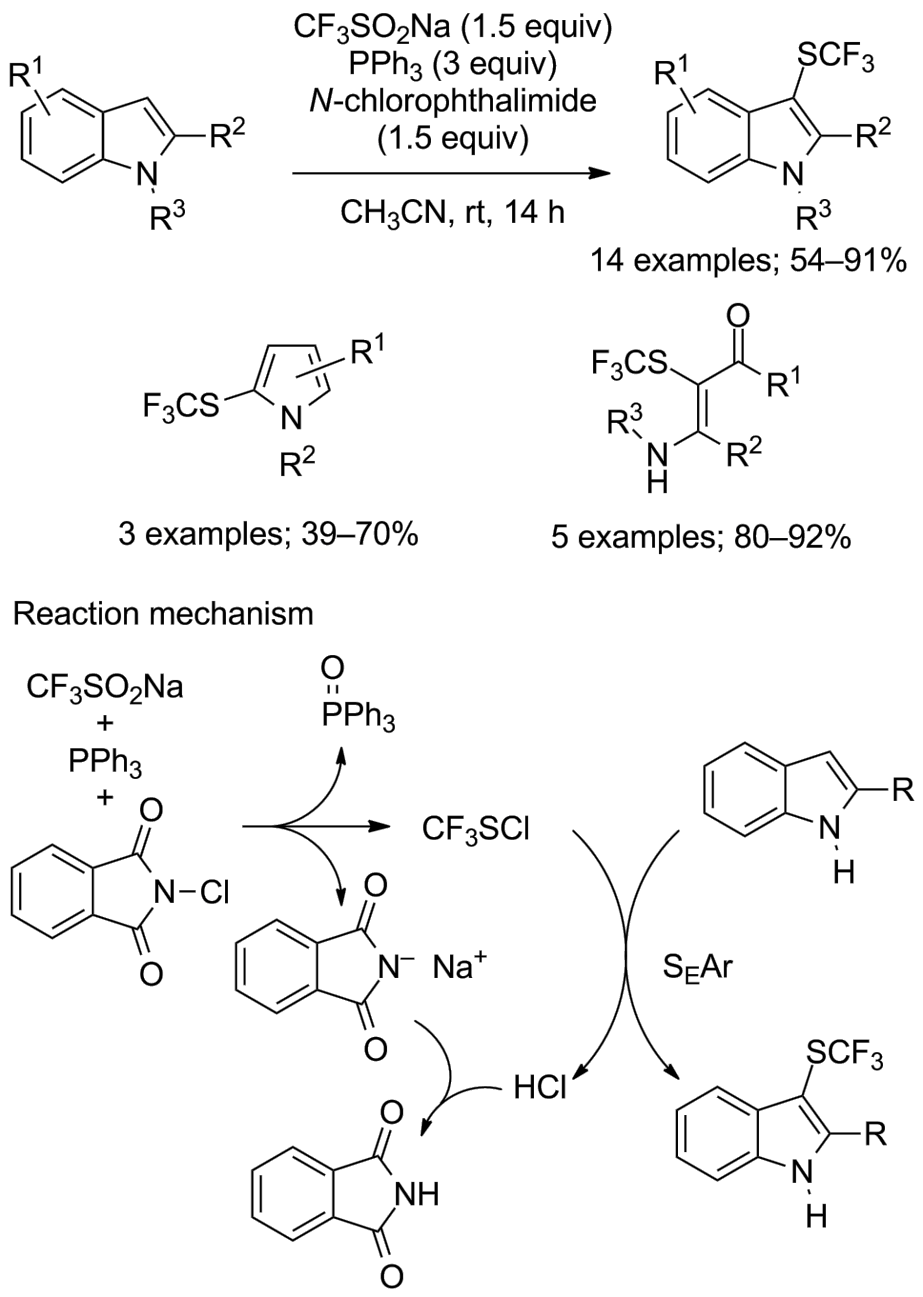
Electrophilic trifluoromethylsulfenylation by means of CF_3_SO_2_Na/PPh_3_/*N*-chlorophthalimide.

Based on this above-mentioned work and simultaneously to the metal-free approach, Cai and co-workers reported the direct trifluoromethylsulfenylation of indoles, pyrroles and enamines, with CF_3_SO_2_Na, triphenylphosphine and *N*-chlorophthalimide without added metal and at room temperature (Scheme 73) [100]. In general, the substrates were successfully trifluoromethylsulfenylated in moderate to excellent yields by in situ generated CF_3_SCl as suggested by ^19^F NMR studies and previous literature reports.

**Scheme 74:**
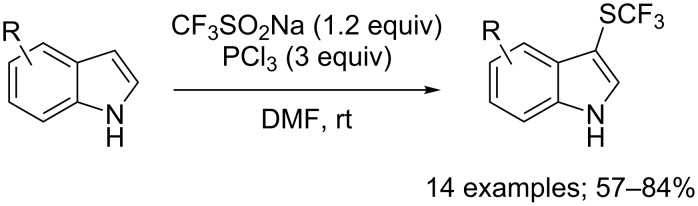
Electrophilic trifluoromethylsulfenylation by means of CF_3_SO_2_Na/PCl_3_.

Different phosphorus reductive reagents were evaluated by Liu, Lin and co-workers. It was found that phosphorus trichloride (PCl_3_) allowed to react with CF_3_SO_2_Na and a series of indoles afforded the corresponding 3-CF_3_S derivatives selectively in good to excellent yields (Scheme 74) [101]. Unlike the work of Cai using PPh_3_ and *N*-chlorophthalimide, here, PCl_3_ was used both as a reducing and chlorinating reagent. ^19^F NMR investigation of reaction intermediates showed the signal of CF_3_SCl, species which was interpreted as the key intermediate in the reaction system. Noteworthy, switching from PCl_3_ to P(O)Cl_3_ allowed the synthesis of the trifluoromethylsulfinyl derivatives instead of the trifluoromethylsulfenyl ones (see next section).

**Scheme 75:**
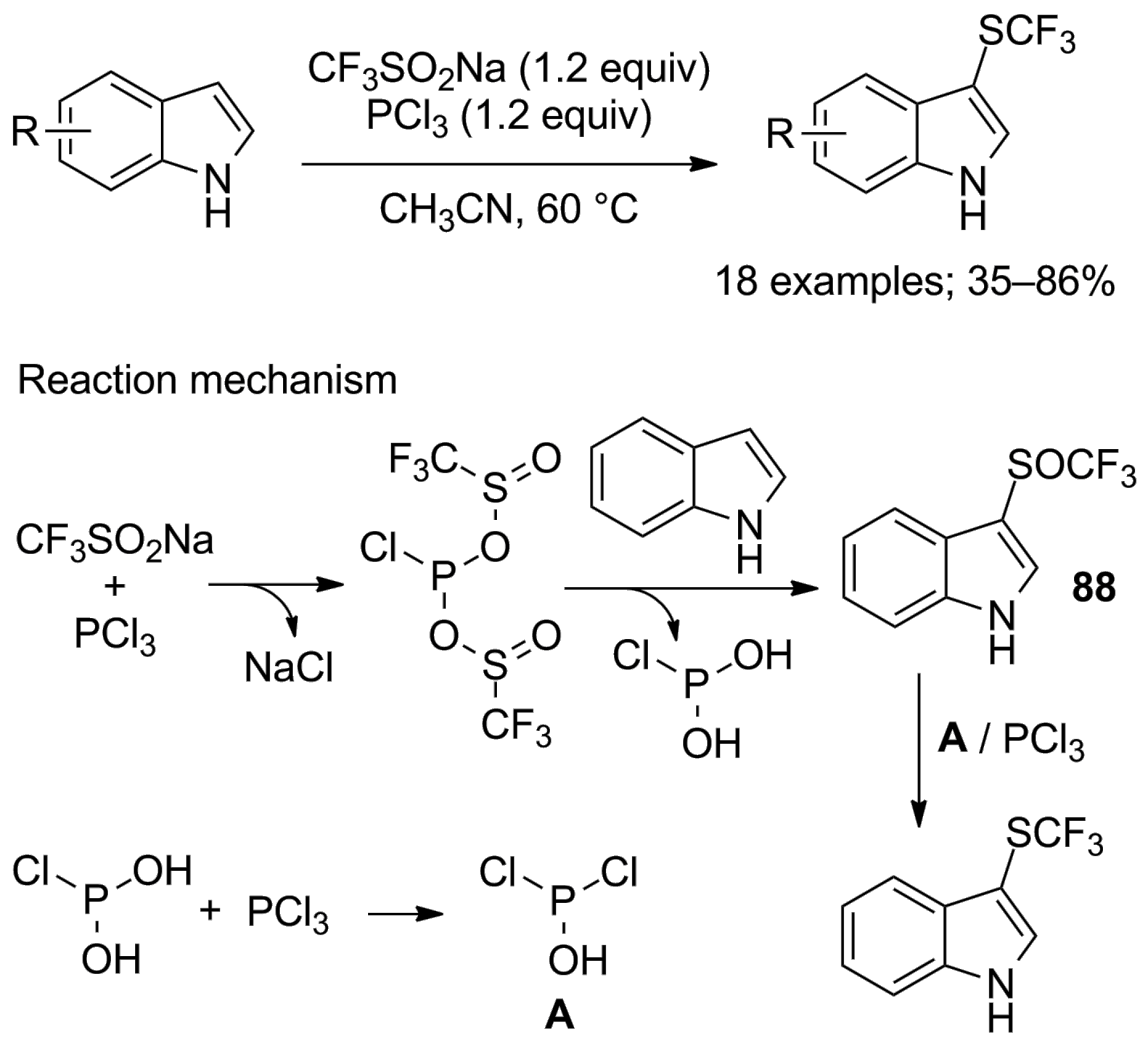
Electrophilic trifluoromethylsulfenylation by means of CF_3_SO_2_Na/PCl_3_.

At the same time, Zhao, Lu and co-workers described similar observations. The trifluoromethylsulfenylation of indoles and electron-rich aromatics was realised with CF_3_SO_2_Na in the presence of PCl_3_ (1.2 instead of 3 equivalents) in acetonitrile at 60 °C (Scheme 75) [102]. The authors proposed a reaction mechanism that involved the 3-trifluoromethylsulfinyl intermediate **88** (Scheme 75). Consequently, a decrease of the amount of PCl_3_ and the temperature was proposed to interrupt the reaction and to obtain **88** selectively (see next section).

**Scheme 76:**
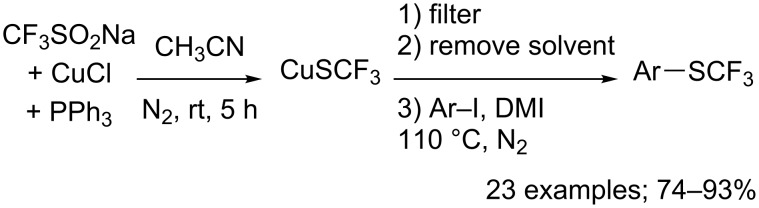
Trifluoromethylsulfenylation of aryl iodides with in situ generated CuSCF_3_ (DMI: 1,3-dimethyl-2-imidazolidinone).

Aryl iodides served as substrates in a programmable regioselective trifluoromethylsulfenylation by direct coupling with CuSCF_3_ generated form CF_3_SO_2_Na in the presence of copper(I) chloride and triphenylphosphine. Indeed, Yang, Vicic and co-workers described the deoxygenative reduction of CF_3_SO_2_Na to form the ligand-free CuSCF_3_ intermediate that reacted with a diverse array of aryl iodides in high yields (Scheme 76) [103]. In addition, CuSCF_3_ was reacted with various ligands to furnish air-stable [LCu(SCF_3_)] complexes as trifluoromethylsulfenylating agents.

### 3 Trifluoromethylsulfinylation

**Scheme 77:**
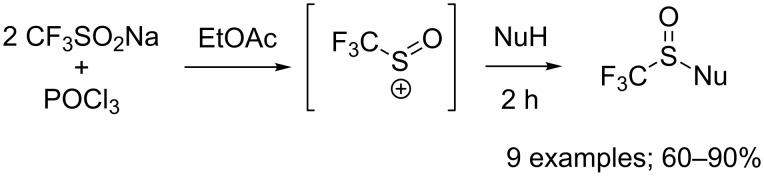
Pioneering trifluoromethylsulfinylation of N, O, and C-nucleophiles.

In 1999, Langlois and co-workers used the system CF_3_SO_2_Na/POCl_3_ as an efficient and inexpensive equivalent of the CF_3_S(O)^+^ cation for the trifluoromethylsulfinylation of various amines, alcohols as well as carbon nucleophiles (Scheme 77) [104]. In the case of primary amines, the addition of one equivalent of diisopropylethylamine to the reaction medium was necessary to trap acidic phosphoric species coming from the reaction and to reach higher product yields. *N*-Methylpyrrole was used as an example of C-nucleophile affording the corresponding S(O)CF_3_ product in 60% yield.

**Scheme 78:**
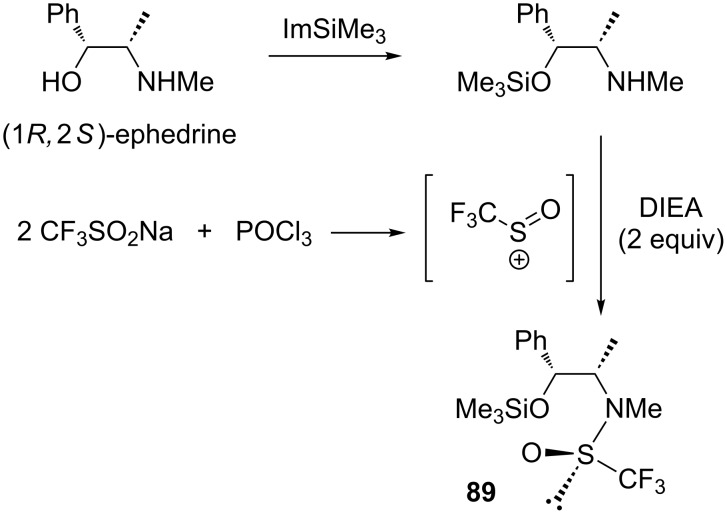
Trifluoromethylsulfinylation of (1*R*,2*S*)-ephedrine (Im: imidazole; DIEA: *N*,*N*-diisopropylethylamine).

A few years later, the same group reported the synthesis of the trifluoromethanesulfinamide **89** derived from (1*R*,2*S*)-ephedrine using the process described above (Scheme 78). Compound **89** was used as an efficient trifluoromethylating agent for a wide range of non-enolisable and enolisable carbonyl compounds [105].

Other *N*-aryl- and *N*-alkyltrifluoromethanesulfinamides have been prepared from the corresponding anilines or aliphatic amines with CF_3_SO_2_Na and phosphoryl chloride for further reactions with arynes [106] or in the synthesis of trifluoromethylsulfonimidates [107].

**Scheme 79:**
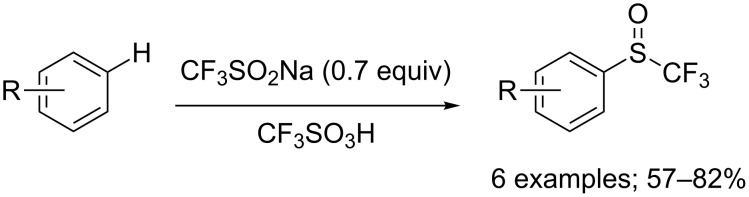
Trifluoromethylsulfinylation of substituted benzenes with CF_3_SO_2_Na/CF_3_SO_3_H.

Another direct trifluoromethylsulfinylation method was described by Wakselman’s group in 2001 with CF_3_SO_2_Na in the presence of triflic acid (Scheme 79) [108]. This method was applied to simple aryls featuring a halogen atom, a OCF_3_ group or an acetanilide function. In this reaction, *ortho*/*para* regioisomers were formed in ratios from 25:75 to 3:97. With regard to the mechanism, the sulfinate was protonated to generate the highly electrophilic CF_3_S(OH)_2_^+^ species that underwent an S_E_Ar with arenes. A further development of the method consisted in the replacement of triflic acid with triflic anhydride in dichloromethane, and this strategy has been applied to the synthesis of trifluoromethylsulfonium salts, known as Umemoto’s reagents [109–111].

**Scheme 80:**
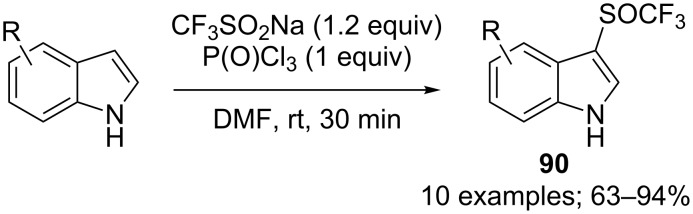
Trifluoromethylsulfinylation of indoles with CF_3_SO_2_Na/P(O)Cl_3_.

After these initial works, it is only in 2017 that new results using CF_3_SO_2_Na appeared in the field. As briefly mentioned in the preceding section, Liu, Lin and co-workers evaluated different phosphorus reductive reagents and found that PCl_3_ (3 equiv) afforded the corresponding 3-CF_3_S derivatives whereas P(O)Cl_3_ (1 equiv) allowed to get selectively the trifluoromethylsulfinyl derivatives **90** (Scheme 80) [101].

**Scheme 81:**
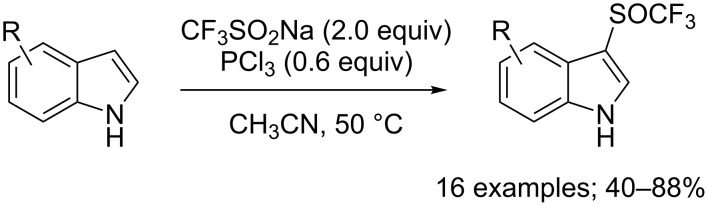
Trifluoromethylsulfinylation of indoles with CF_3_SO_2_Na/PCl_3_.

With the same objective of partial reduction of CF_3_SO_2_Na with phosphorus reagents for the selective trifluoromethylsulfinylation, Zhao, Lu and co-workers noticed that a decrease of the amount of PCl_3_ and a slight lowering of the temperature were beneficial to the desired trifluoromethylsulfinylation (Scheme 81) [102].

### 4 Trifluoromethylsulfonylation

The trifluoromethylsulfonyl motif (SO_2_CF_3_, Tf, triflyl) is highly electron-withdrawing (SO_2_CF_3_, σ_m_ = 0.79, σ_p_ = 0.93) and trifluoromethylsulfones (or triflones), in which the trifluoromethylsulfonyl group is attached to a carbon atom, are moderatly lipophilic (SO_2_CF_3_, π = 0.55 versus CF_3_, π = 0.88). Therefore, the SO_2_CF_3_ group has been frequently employed in catalysts and ligands, as subunit of bioactive compounds, or as building block for advanced functional materials. Trifluoromethylsulfones have been prepared by several approaches [5], in particular by means of CF_3_SO_2_Na as compiled hereafter.

#### C_sp3_–SO_2_CF_3_ bond-forming reactions

**Scheme 82:**
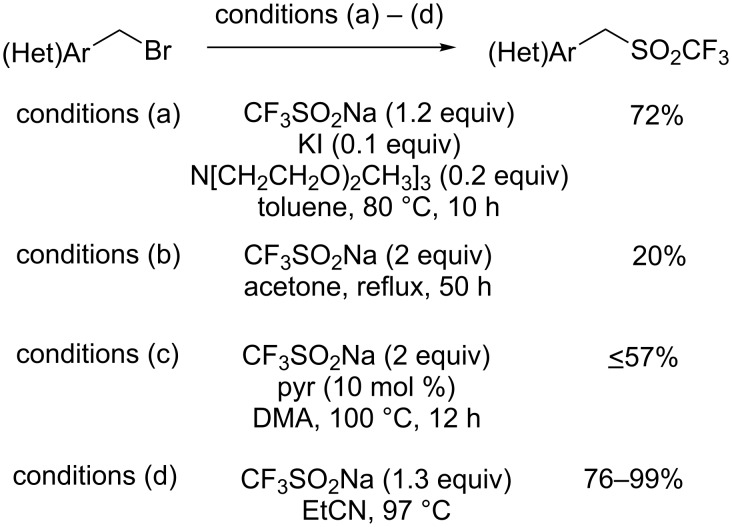
Formation of triflones from benzyl bromides (DMA: dimethylacetamide).

The trifluoromethanesulfinate, triflinate anion, possesses a low nucleophilicity thus affecting its potential in substitution reactions. Mainly primary bromides and some secondary ones (α-bromo ketones and esters) could be converted into trifluoromethylsulfones. From benzyl bromides, phase-transfer conditions [112] and/or high temperatures [113–115] were required to obtain the triflones in low to high yields (Scheme 82). Using benzyl chloride in propionitrile at reflux led to 90% yield of the corresponding triflone [115]. Methyl iodide was also used as substrate for the formation of methyl trifluoromethylsulfone [116]. The displacement of a tosyl group by CF_3_SO_2_Na in the presence of *n*-Bu_4_NI in THF worked with 60 % yield [117].

**Scheme 83:**
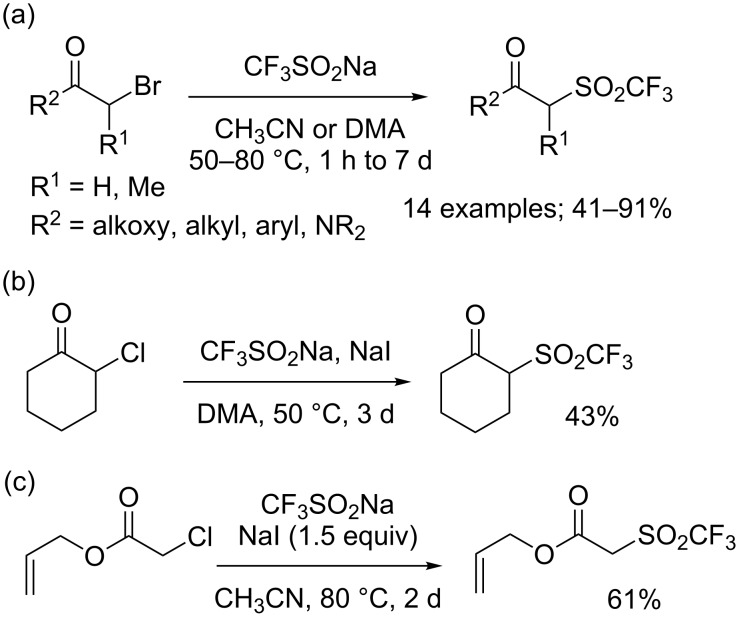
Formation of α-trifluoromethylsulfonyl ketones, esters, and amides.

α-Trifluoromethylsulfonyl ketones, esters, and amides were prepared by displacement of the corresponding bromide (Scheme 83a) [112,118] or chloride (Scheme 83b,c) [118–119] under various conditions. Long reaction times and high temperatures were often required.

**Scheme 84:**
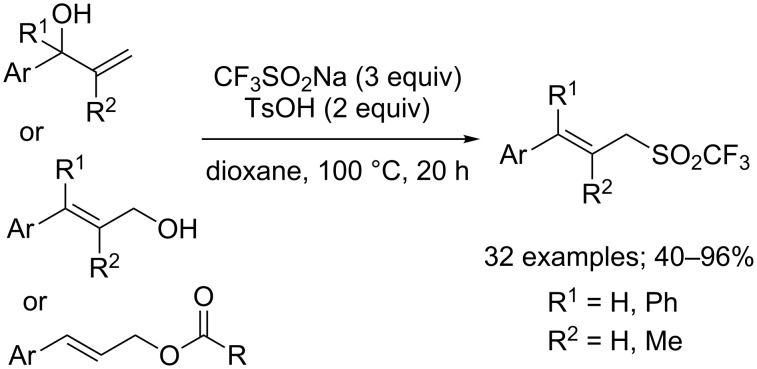
Allylic trifluoromethanesulfonylation of aromatic allylic alcohols.

Allylic trifluoromethylsulfones could be prepared from aromatic allylic alcohols or esters and CF_3_SO_2_Na in the presence of *p*-toluenesulfonic acid at 100 °C in dioxane. The reaction was highly regioselective, worked well with primary, secondary and tertiary allylic alcohols, and tolerated a wide range of functions (Scheme 84) [120]. The reaction proceeded through an S_N_1-type mechanism by the formation of a cationic Π-allyl intermediate by means of *p*-toluenesulfonic acid.

#### C_sp2_–SO_2_CF_3_ bond-forming reactions

**Scheme 85:**
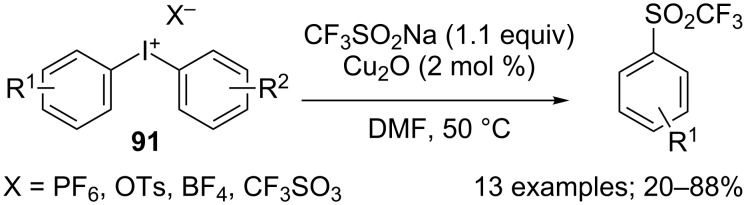
Copper-catalysed couplings of aryl iodonium salts with CF_3_SO_2_Na.

**Aryl triflones:** The synthesis of aryl trifluoromethylsulfones was investigated by Shekhar and co-workers in 2013. First, Pd- and Cu-catalysed coupling reactions with iodobenzene or arylboronic acids failed because of the poor nucleophilicity of CF_3_SO_2_Na [121]. It was next found that aryl iodonium salts reacted with CF_3_SO_2_Na in the presence of copper catalysts. In particular cuprous oxide in DMF gave the triflones in moderate to high yields from a variety of symmetrical and unsymmetrical (het)aryl iodonium salts **91** bearing various functional groups and counteranions (Scheme 85) [121]. The reaction was sulfonyl-retentive and was likely to proceed via a nonradical pathway, probably involving Cu(I)/Cu(III) intermediates.

Another example of trifluoromethanesulfonylation of a diaryl iodonium salt was reported by Li and co-workers as a single case, among several other nucleophiles, using 10 mol % of copper(II) triflate in dichloroethane at 80 °C [122]. The hypervalent iodine species could also be generated in situ [123].

**Scheme 86:**
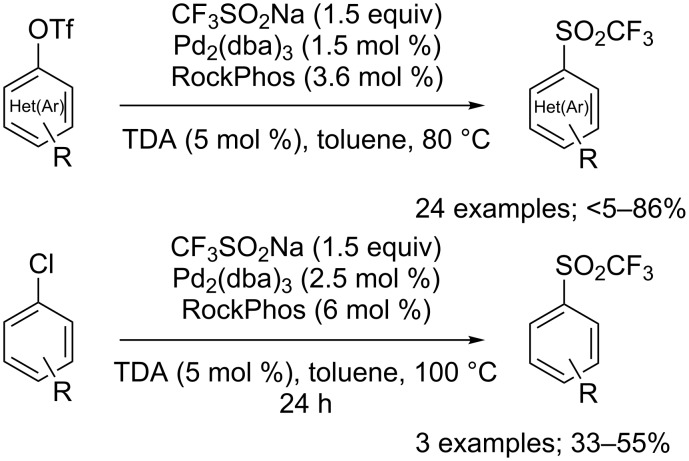
Palladium-catalysed trifluoromethanesulfonylation of aryl triflates and chlorides with CF_3_SO_2_Na.

In 2016, Shekhar’s group finally succeeded in the trifluoromethanesulfonylation of the more readily available (het)aryl triflates and some aryl chlorides using a combination of Pd_2_(dba)_3_ and the bulky RockPhos phosphine ligand in toluene and tris(3,6-dioxaheptyl)amine (TDA) as phase-transfer catalyst to facilitate the reaction with the sparingly soluble CF_3_SO_2_Na (Scheme 86) [124].

**Scheme 87:**
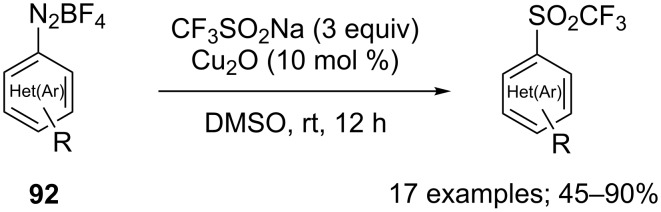
Copper-catalysed coupling of arenediazonium tetrafluoroborates with CF_3_SO_2_Na.

Arenediazonium tetrafluoroborates **92** were also suitable substrates for the formation of triflones. Xu, Qing and a co-worker demonstrated that CF_3_SO_2_Na produced triflones when engaged in a Cu-catalysed reaction in DMSO (Scheme 87) [76]. The reaction was sulfonyl-retentive whereas in the presence of the oxidant *tert*-butyl hydroperoxide the chemoselective trifluoromethylation was obtained (see Scheme 52).

**Scheme 88:**
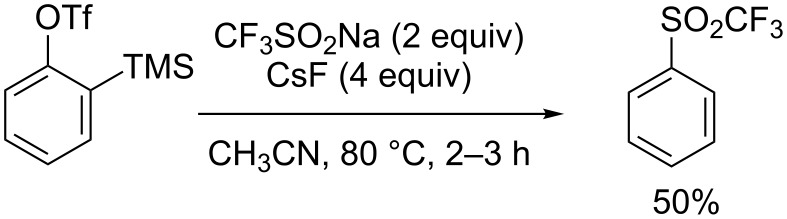
Synthesis of phenyltriflone via coupling of benzyne with CF_3_SO_2_Na.

Finally, a metal-free protocol was reported by Singh and co-workers, in which in situ generated benzyne was reacted with CF_3_SO_2_Na (Scheme 88) [125].

**Scheme 89:**
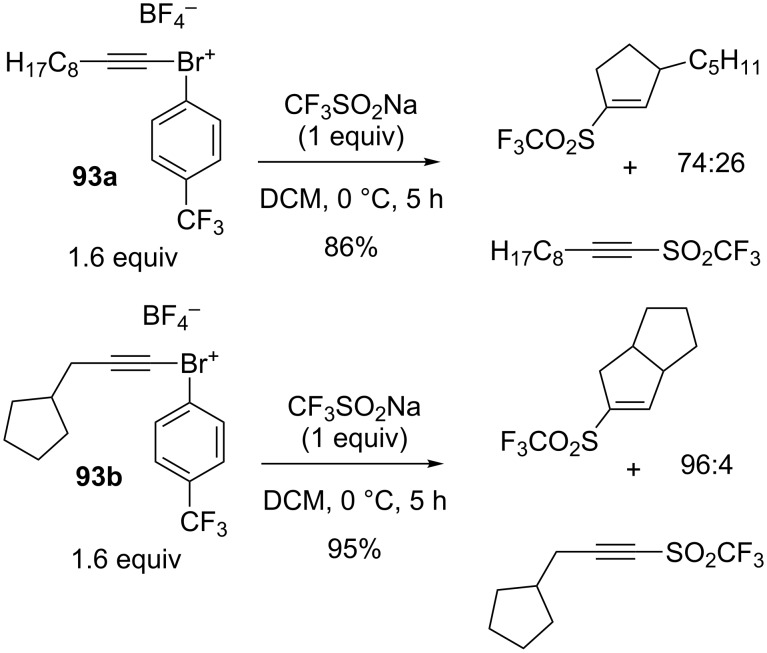
Synthesis of 1-trifluoromethanesulfonylcyclopentenes from 1-alkynyl-λ^3^-bromanes and CF_3_SO_2_Na.

**Vinyl triflones:** The synthesis of two vinyl trifluoromethylsulfones was reported in 2004 by Ochiai and co-workers by treatment of λ^3^-bromane **93** with CF_3_SO_2_Na in DCM at 0 °C (Scheme 89) [126]. The reaction also produced 1-alkynyltriflones as minor products. The 1-alkynyl-λ^3^-bromanes acted as Michael acceptors for the sulfinate anion and underwent tandem Michael addition–carbene insertion reactions to yield the 1-trifluoromethanesulfonylcyclopentenes.

**Scheme 90:**
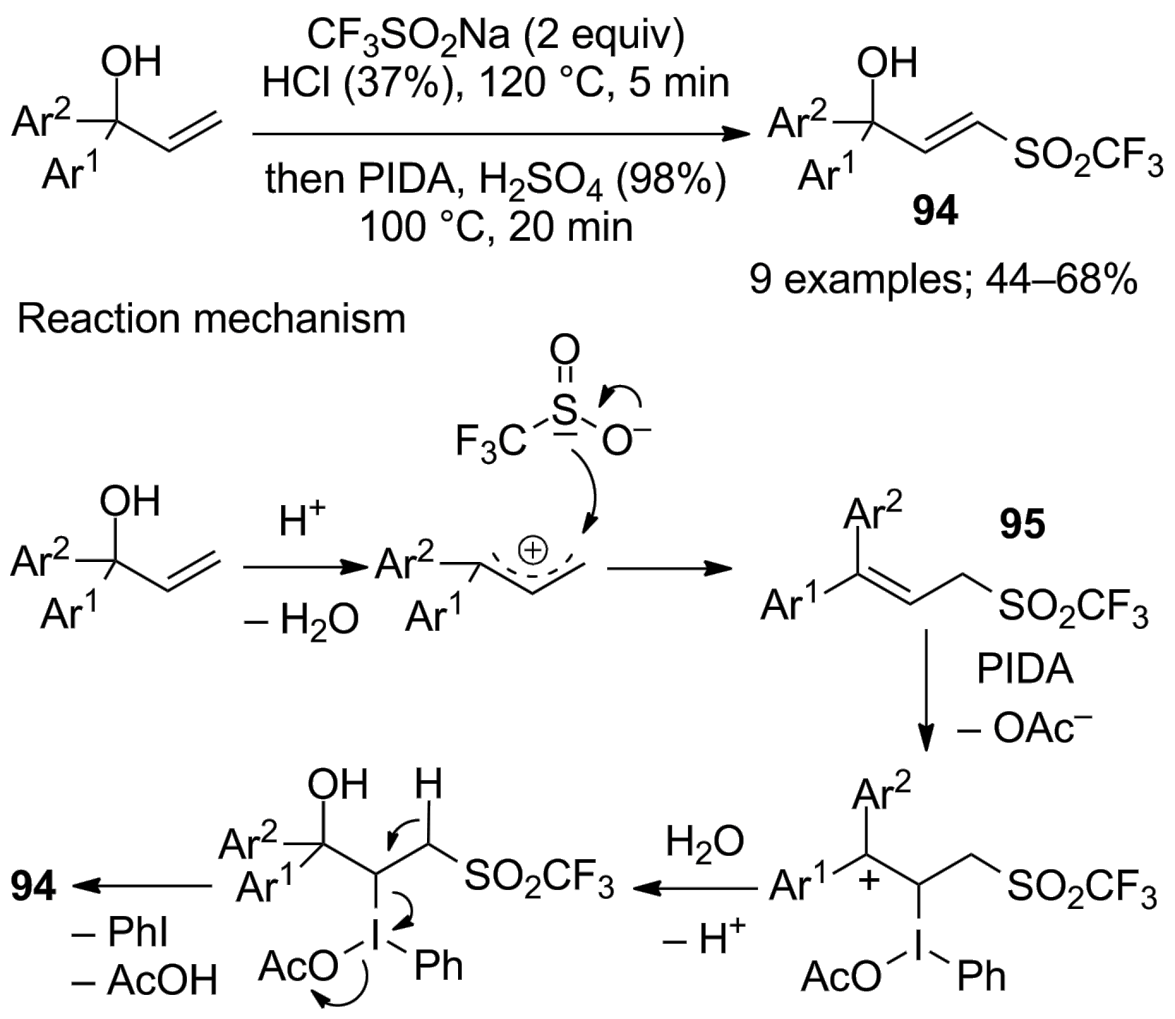
One-pot synthesis of functionalised vinyl triflones.

Vinyl triflones with exclusive *E*-selectivity could also be prepared from allylic alcohols and CF_3_SO_2_Na under metal-free conditions as reported by Xu, Ji and co-workers (Scheme 90) [127]. Under acidic conditions, the π-allylic carbocation reacted with the sulfinyl anion leading to allylsulfone **95**, which underwent an electrophilic addition of PIDA and capture of the benzylic carbocation by water. Then, a concerted proton elimination and C–I bond cleavage occurred to give the triflylated allylic alcohol **94**.

**Scheme 91:**
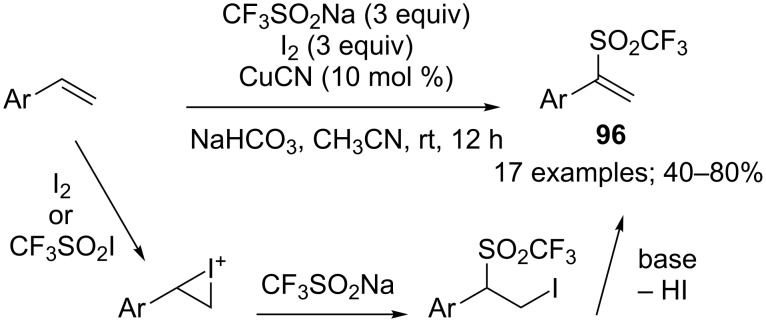
Regioselective synthesis of vinyltriflones from styrenes.

Huang, Xu and co-workers demonstrated that styrenes, but also vinylbenzofuran and vinylthiophene, reacted with CF_3_SO_2_Na in the presence of iodine and CuCN to afford regioselectively the internal vinyl triflones **96** (Scheme 91) [128]. A cationic reaction mechanism was proposed by electrophilic addition of I_2_ (or the in situ generated CF_3_SO_2_I) to the olefin. The highly strained iodonium bridge subsequently reacted with the sulfinyl anion to afford the iodotriflylated product, which eliminated HI to give the vinyl triflone. The role of CuCN was not precised at this stage.

#### C_sp_–SO_2_CF_3_ bond-forming reactions

**Scheme 92:**
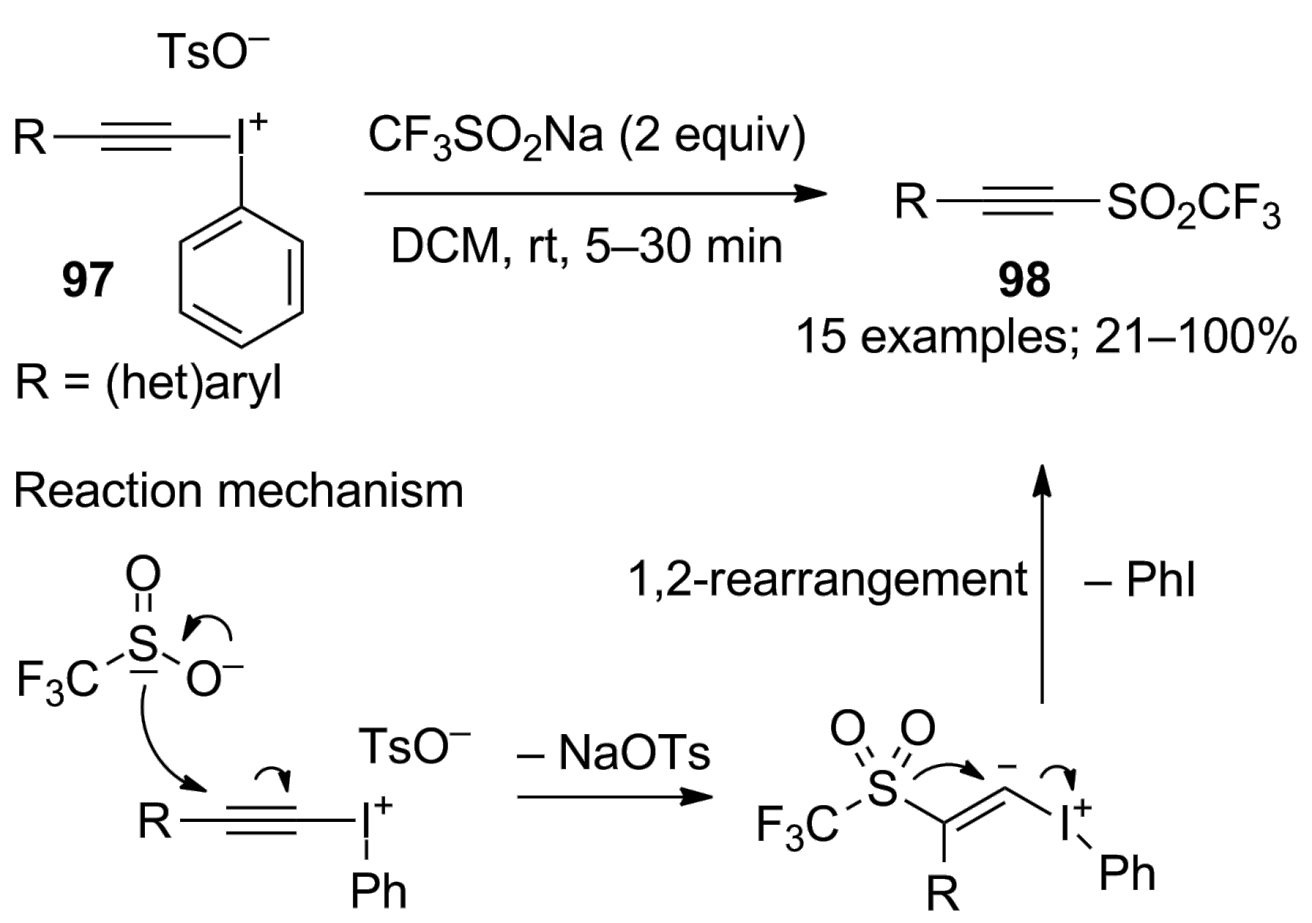
Trifluoromethanesulfonylation of alkynyl(phenyl) iodonium tosylates by CF_3_SO_2_Na.

Apart from the side products described in Scheme 89, a variety of acetylenic triflones **98** was proposed by Zhang and co-workers. Alkynyl(phenyl) iodonium tosylates **97** reacted with CF_3_SO_2_Na in a transition metal-free protocol to furnish the acetylenic triflones under mild conditions (Scheme 92) [129]. The authors suggested that alkylidene carbene intermediates might be formed followed by a 1,2-rearrangement to yield the triflones. The reaction from ethynylbenzene and CF_3_SO_2_Na in the presence of Koser’s reagent allowed to obtain the corresponding triflone albeit in a 33% yield.

#### S–SO_2_CF_3_ and Se–SO_2_CF_3_ bond-forming reactions

**Scheme 93:**
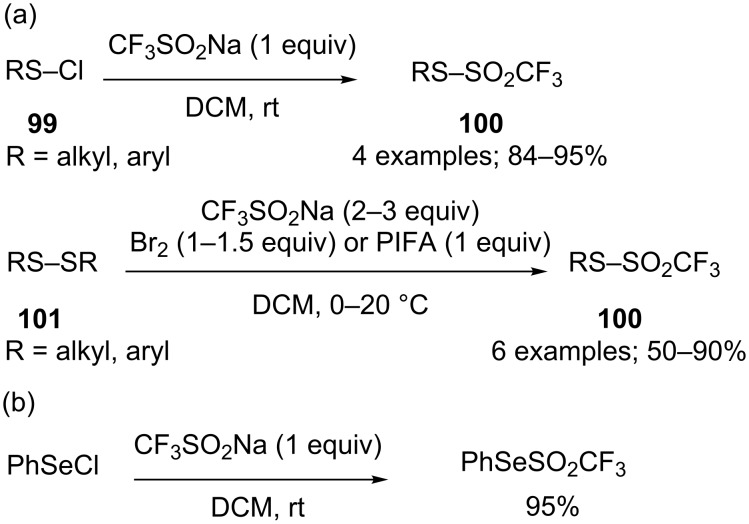
Synthesis of thio- and selenotrifluoromethanesulfonates.

Langlois’ group prepared thiotrifluoromethanesulfonates **100** from sulfenyl chlorides **99** or disulfides **101** by two methods: first, sulfenyl chlorides were reacted with CF_3_SO_2_Na in DCM at room temperature [130]; second, a wider scope was obtained by reaction of disulfides with CF_3_SO_2_Na under oxidative conditions with either bromine [130] or PIFA [131] (Scheme 93a). These methodologies were extended to phenylselenyl chloride and diphenyl diselenide (Scheme 93b).

## Conclusion

Sodium trifluoromethanesulfinate was used for the first time in direct radical trifluoromethylation under oxidative conditions by the Langlois group in 1991. Since that time its chemistry has evolved sporadically till 2011 and then immensely thanks to Baran’s contribution. It enjoyed a rapid growth not only as donor of the trifluoromethyl group but also as a trifluoromethylsulfenylating agent, for the transfer of the whole SCF_3_ motif, and as trifluoromethanesulfinylating agent for S(O)CF_3_ transfer. CF_3_SO_2_Na is an easy-to-handle, inexpensive reagent that has became versatile for the construction of sophisticated fluorinated products in the pharmaceutical chemistry. It is the authors’ hope that this review will contribute to stimulate further research toward new reactions and applications for this reagent that could include the formation of new bonds with other heteroatoms, novel tandem reactions, and the exploration of asymmetric reactions, so far absent with CF_3_SO_2_Na, to cite but a few examples.
